# Efficacy of Probiotics Compared With Pharmacological Treatments for Maintenance Therapy for Functional Constipation in Children: A Systematic Review and Network Meta-analysis

**DOI:** 10.1093/nutrit/nuae119

**Published:** 2024-09-30

**Authors:** Rebecca G Harris, Elizabeth P Neale, Marijka Batterham

**Affiliations:** School of Medical Indigenous and Health Science, Faculty of Science, Medicine, and Health, University of Wollongong, Wollongong, New South Wales 2522, Australia; Illawarra Health and Medical Research Institute, Wollongong, New South Wales 2522, Australia; School of Medical Indigenous and Health Science, Faculty of Science, Medicine, and Health, University of Wollongong, Wollongong, New South Wales 2522, Australia; Illawarra Health and Medical Research Institute, Wollongong, New South Wales 2522, Australia; Illawarra Health and Medical Research Institute, Wollongong, New South Wales 2522, Australia; Statistical Consulting Centre, School of Mathematics and Applied Statistics, Faculty of Engineering and Information Sciences, University of Wollongong, Wollongong, New South Wales 2522, Australia

**Keywords:** children, functional constipation, network meta-analysis, laxatives, probiotics, randomized controlled trials, systematic review, maintenance therapy

## Abstract

**Context:**

There has been an increase in randomized controlled trials (RCTs) comparing probiotics with various maintenance therapies, such as polyethylene glycol, lactulose, and mineral oil, to treat functional constipation in children.

**Objective:**

The aim was to compare probiotics with all other oral maintenance therapies for functional constipation in children and rank all treatments in terms of effectiveness in a network meta-analysis.

**Methods:**

RCTs were identified through systematically searching the MEDLINE, Scopus, EMBASE, and Cochrane Library databases, trial registries, and forward and backward citation searching. Within-study risk of bias was assessed using the Cochrane Risk of Bias 2 tool, and confidence in the estimates was assessed using the CINeMA (Confidence in Network Meta-Analysis) framework. Random-effects network meta-analyses were conducted.

**Results:**

Data were pooled from 41 and 29 RCTs for network meta-analysis of defecation frequency and treatment success, respectively. Probiotics did not significantly increase the number of bowel movements per week when compared with any conventional treatment or placebo. A combination of mineral oil and probiotics was the most effective treatment for increasing defecation frequency (mean difference: 3.13; 95% confidence interval [CI]: 0.64, 5.63). The most effective treatments for increasing the risk of treatment success as compared with placebo were mineral oil (relative risk [RR]: 2.41; 95% CI: 1.53, 3.81) and a combined treatment of polyethylene glycol and lactulose (RR: 2.45; 95% CI: 1.21, 4.97). Confidence in the estimates ranged from very low to moderate.

**Conclusion:**

Currently, there is no evidence to suggest that probiotics should be used as a standalone treatment for functional constipation in children. More high-quality studies are needed to evaluate different strains of probiotics and their potential benefit as an additional treatment component to conventional treatments. Mineral oil and polyethylene glycol were the most effective treatments to increase defecation frequency and treatment success rates and should remain the first line of treatment for children with functional constipation.

**Systematic Review Registration:**

PROSPERO registration no.

CRD42022360977 (https://www.crd.york.ac.uk/prospero/display_record.php?RecordID=360977).

## INTRODUCTION

Constipation in children is a widespread pediatric healthcare problem, affecting up to 30% of children and adolescents worldwide.^1^^,^^2^ In the absence of an underlying organic cause, children with chronic constipation are diagnosed with functional constipation (FC), which makes up the majority of constipation cases in this age group. The major cause of FC is understood to be voluntary withholding behavior, due to fear of defecation after a previous painful bowel movement. This leads to a vicious cycle of hardened and impacted stools that are difficult to pass, furthering fear and withholding behavior.

Immediate treatment for FC involves dis-impaction of the hardened stool. Maintenance therapy is then required to prevent future withholding behavior and fecal impaction. There are various pharmacological treatment options to improve stool frequency during the maintenance period. Current guidelines recommend polyethylene glycol (PEG) as the first line of treatment and lactulose as a second choice when PEG is unavailable or if the child is allergic to PEG.^3–5^ These treatments are classed as osmotic laxatives and increase passive water flow into the intestinal lumen, making it easier for stool to pass through the intestinal tract.^5^^,^^6^ Since FC does not have an organic cause, it is often the case that children treated with conventional pharmacological therapies continue to experience symptoms of FC.

Probiotics have emerged as a potential treatment when pharmacological treatments are not effective in successfully treating patients. Probiotics are live microorganisms, which, when administered in adequate amounts, may provide health benefits for the host.^7^ It has been proposed that the substances produced by certain beneficial bacteria could improve colonic motility by softening the stool, decreasing inflammation, and increasing the secretion of water.^8^^,^^9^ Therefore, increasing the amount of bacteria by taking an oral probiotic could increase the amount of these substances and improve symptoms of FC. Previous reviews have compared probiotic consumption with different treatments, including placebo, osmotic laxatives, magnesium oxide, and mineral oil, and found that there was insufficient evidence to conclude whether probiotics are efficacious in successfully treating FC or increasing defecation frequency in children with FC.^10^^,^^11^ These reviews consisted of conducting underpowered, separate, pairwise meta-analyses for each comparison of probiotics to currently available treatments. Therefore, probiotics have not been compared with all available treatments in a single network meta-analysis model.

A network meta-analysis is able to summarize evidence across a network of available treatments. This methodology utilizes both direct comparisons observed in the research and indirect evidence, which is generated when treatments are compared with a common comparator but not directly within the same trial.^12^ It would be of benefit for clinicians for probiotics to be compared with other maintenance therapies in a network meta-analysis. This would enable each treatment to be ranked from the highest to the lowest probability of being the most effective, providing valuable information for clinicians to determine which treatment to administer to their patients. This systematic review and network meta-analysis aimed to compare probiotics with oral pharmacological maintenance therapies to improve defecation frequency and treatment success rates in children with FC.

## METHODS

This review was carried out following the guidelines from the Cochrane Handbook for Systematic Reviews of Interventions, version 6.3,^13^ and reported according to the PRISMA (Preferred Reporting Items for Systematic reviews and Meta-Analyses) for Network Meta-analyses statement^14^ (Table S1). The methods, including the eligibility criteria, search strategy, extraction process, and analysis, were prespecified and documented in a protocol that was published in PROSPERO as CRD42022360977 (available at: https://www.crd.york.ac.uk/prospero/display_record.php?RecordID=360977).

### Eligibility criteria

Studies were eligible if they met the predefined eligibility criteria according to the PICOS (Population, Intervention, Comparator, Outcome, Study design) framework outlined in Table 1.^15–19^ Since this systematic review contains a network meta-analysis, studies were eligible if they compared a combination of any of the eligible treatments. This means that the comparison arm of the randomized controlled trial (RCT) may contain an active treatment rather than a placebo.

**Table 1.** PICOS Criteria for Inclusion of Studies

Parameter	Criterion
P (Population)	RCTs of interest were those that were confined to children (aged <18 y) diagnosed with FC according to the Rome II, III, or IV criteria^15–19^ or variations of these as defined by the authors. RCTs including patients with an organic cause for constipation or with a history of colorectal surgery were thus not eligible.
I (Intervention)	RCTs eligible for inclusion were those that evaluated any of the following treatments: • mineral oil • lactulose • magnesium hydroxide • sorbitol • PEG • senna syrup • bisacodyl tablets • probiotics Maintenance therapies considered were those that are orally administered and commonly used as maintenance therapy after dis-impaction in children. Laxatives that are rectally administered (enemas), bulk-forming laxatives, or laxatives used for the treatment of constipation caused by chronic opioid use were not included. All probiotic (single or mixture) strains, doses, treatment regimens, and means of administration (tablets, capsules, powder, or fortified foods) were considered. Synbiotics, which are a mixture of pre- and probiotics, were also included.^18^^,^^19^
C (Comparator)	Any treatment arm described above or placebo.
O (Outcome)	Primary outcomes of interest were the differences between treatment groups for key outcomes used to ascertain improvement of symptoms. These include defecation frequency as a continuous outcome (BMs/wk) and treatment success as a categorical outcome (the number of participants who no longer met the criteria of having FC). Adverse events were evaluated qualitatively as a secondary outcome by considering the difference in rates and the types of events experienced for all treatment groups.
S (Study design)	Only RCTs were eligible for inclusion in this systematic review. Studies that used nonrandom or quasi-random methods to assign patients to treatment groups (eg, alternate allocation) were not eligible. Studies published only as an abstract (eg, conference proceedings) were also ineligible.
Language	No restrictions on language of the article.
Year	No restrictions on year of publication.

*Abbreviations*: BM, bowel movement; FC, functional constipation; PEG, polyethylene glycol; RCT, randomized controlled trial.

### Search strategy and study selection

To identify relevant articles, the MEDLINE (via the PubMed interface), Scopus, EMBASE (via the Ovid interface), and Cochrane Library databases were systematically searched (Table S2). All searches were conducted without date or language restrictions from inception until June 18, 2022. Handsearching was conducted using backward (manually) and forward (with Google Scholar) citation checks to identify any additional relevant papers, by examining the reference lists of previous systematic reviews and all relevant papers selected from the search of the bibliographic databases and any subsequent publications that cited these papers. Finally, the World Health Organization (WHO) International Clinical Trial Registry Platform (https://www.who.int/ictrp/search/en/) was searched, which offers a single point of access to trials included in individual registries worldwide, to identify completed and any planned or ongoing RCTs.

The titles and abstracts of all identified references were screened independently by 2 reviewers (R.G.H. and E.P.N.) using Covidence (Veritas Health Innovation, Melbourne),^20^ a web-based collaboration software platform. Copies of the full texts were obtained if the studies seemed relevant and were imported into the Covidence platform. R.G.H. and E.P.N. read the full texts and assessed their content against the eligibility criteria independently. In cases of disagreement, consensus was reached through discussion involving a third author (M.B.).

### Data extraction

A data-extraction form was used to record information on the studies’ relevant characteristics and results. Relevant characteristics retrieved included the following: the number of patients randomized to each interventions, the type of intervention and comparator, duration of the treatment, duration of constipation symptoms, patients’ age range, mean age of the patients, percentage of female patients, the primary and secondary outcomes investigated, any co-interventions allowed, and whether their uptake was monitored and reported, and sources of funding (including role in RCT conduct and reporting) and declarations of conflicts of interest of the primary researchers.

From each study, and for each intervention group separately, the sample size (*n*), the mean level, and the SD or, if provided instead, the SE or 95% confidence interval (CI) of defecation frequency before and after treatment were extracted. If studies reported only mean changes from baseline and the respective SD (or SE) for each group, these were extracted and used in further analyses. A mixture of change-from-baseline and follow-up scores (and respective SDs or SEs) can be pooled directly in meta-analysis of (unstandardized) mean differences (MDs) because, in an RCT, and in the absence of imbalances at baseline, MDs based on changes from baseline can be assumed to be addressing exactly the same underlying intervention effects and will have approximately the same magnitude as analyses based on follow-up scores.^13^ When only SEs were provided, these were converted to SDs prior to data synthesis. The numbers of patients in each intervention arm at follow-up who achieved treatment success were also extracted. When outcome measures were reported at multiple time points, data extracted were those that most closely aligned with the most commonly reported time point (4 weeks). Whenever the data provided in published reports were incomplete or unclear, the authors were contacted to obtain further details.

The data extracted and used in network meta-analysis for both outcomes of defecation frequency and treatment success are presented in the supplementary material (Table S3). The R code used to run analyses is also presented in the supplementary material (Table S4).

### Statistical analyses: data synthesis and meta-analysis

Network meta-analyses were conducted with R software (version 4.2.3; www.r-project.org; R Foundation for Statistical Computing, Vienna, Austria) using the netmeta package.^21^ Random-effects network meta-analysis models were used for the MD in defecation frequency (bowel movements [BMs]/wk) and relative risk (RR) of treatment success. This was done for each outcome separately using probiotics as the reference treatment group. Forest plots were used to succinctly display only the comparisons between probiotics and all other treatments. League tables were used to present effect sizes for all comparisons generated from the network meta-analyses and the effect sizes generated using only direct evidence. Treatments were ranked according to the probability that they are the most effective treatment by estimating *P* scores for each treatment.^22^  *P* scores are the frequentist analogue to SUCRA from a Bayesian network meta-analysis.^23^ An additive network meta-analysis was also conducted, which assumes that the effect of a combined treatment is the sum of the individual treatment components and that common components cancel out in comparisons. Conventional treatments were also directly compared with a combined treatment group of the conventional treatment and probiotics. These effect estimates were obtained by changing the reference group of the network meta-analyses to the conventional treatment of interest. This was done for PEG, mineral oil, lactulose, and magnesium hydroxide, as these were the only comparisons reported. Note that this does not run a new network meta-analysis model but simply changes the reference group so that the effects of the combined treatment are displayed relative to the standalone treatment. These effects are of the same magnitude as those displayed in the league tables, but RRs may be inverted and MDs may have an opposite sign when the league table compares the effects of the standalone treatment relative to the combined treatment group (ie, the reference group is switched).

We also assessed the transitivity assumption, which implies that the treatment effects observed through direct evidence would be the same in other trials where the treatment effects were unobserved because they compared different treatments. This was done by comparing characteristics that can affect the impact of the treatment on the outcome stratified by treatment group. This was conducted for the subset of trials that reported on defecation frequency and treatment success separately. The main characteristics of interest were BMs per week at baseline, duration of constipation, length of follow-up, sample-size percentage of participants who are female, and publication year.

### Assessment of the certainty of the evidence

We then used the CINeMA (Confidence in Network Meta-Analysis) framework^24^ to assess the confidence in the treatment effects. The CINeMA framework encompasses 6 domains that affect the confidence in the results, including within-study bias, reporting bias, indirectness, imprecision, heterogeneity, and incoherence. The CINeMA web application^25^ (https://cinema.ispm.unibe.ch/) was used to incorporate information for each domain and give a rating of “high,” “moderate,” “low,” or “very low” for both direct and indirect evidence of each comparison in the network. Evidence for each comparison started at “high” and was downgraded 2 levels to “low” if there was a judgment of major concerns in 1 of the domains. A judgment of some concerns in 1 domain led to a downgrade of 1 level to “moderate.” When there were multiple domains with “some concerns” or “major concerns,” these were considered jointly rather than in isolation because concerns in 1 domain are often related to the concerns in other domains and downgrading multiple times for related concerns is not advised.^24^ A dataset containing relevant data needed for analysis as well as the ratings for within-study bias and indirectness assessments for each study was uploaded to the CINeMA web application. This was done separately for the dataset of defecation frequency and treatment success.

To assess within-study risk of bias (RoB) of each included study the Cochrane Collaboration's RoB 2 tool for assessing RoB in RCTs was used.^26^ Specifically, the potential bias in each domain of the tool (Domain 1: randomization, Domain 2: deviation from intended interventions, Domain 3: missing outcome data, Domain 4: measurement of the outcome, and Domain 5: selection of reported results) were rated as either “low risk,” “high risk,” or “some concerns.” This was achieved by first answering the relevant signaling questions for each domain and then using the algorithms provided in the tool to determine a rating for each domain and for the RCT as a whole. The user of the CINeMA web application is required to select either the average, majority, or highest overall ratings within each comparison for use in assessment of the overall confidence rating of each comparison. The average of the within-study bias ratings across each comparison was used.

Reporting bias for each comparison was assessed by visual inspection of comparison-adjusted funnel plots for publication bias. It also considered whether it was likely for studies included in the review but not in meta-analysis as well as trial protocols with nonpublished results to have missing evidence based on the results.

Indirectness was assessed as “low,” “moderate,” or “high” for each study based on relevance to the research question. Studies that had a follow-up longer than 8 weeks or a number of BMs per week greater than 3 had the level of directness downgraded by 1 level.

Judgments for imprecision, heterogeneity, and inconsistency are generated automatically in CINeMA based on a clinically important effect size specified by the user. An MD of 1 BM/wk and a RR of 1.5 were selected as clinically important effect sizes for defecation frequency and treatment success, respectively. Statistical tests were conducted using the netmeta package in R to evaluate consistency. Local inconsistency in the network meta-analysis models was assessed by comparing the direct and indirect evidence with the Separate Indirect from Direct Design Evidence (SIDDE) test using the netsplit command. A design-by-treatment interaction model was used to further evaluate global consistency in the networks using the decompose design function.

## RESULTS

### Study characteristics

**Figure 1.**
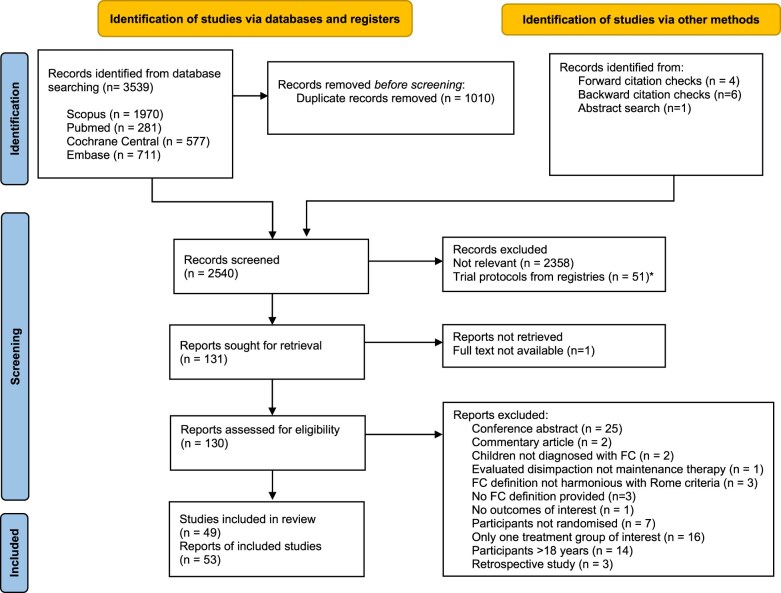
Flow Diagram Describing the Study Selection Process. *Trial protocols published in online registries identified by the search were matched to published articles of the corresponding RCTs where available. Abbreviations: FC, functional constipation; RCT, randomized controlled trial

After removal of duplicates, 2529 unique records were identified through database searching (Figure 1). Eleven additional articles were identified through forward citation checks, leading to 2540 unique reports. Trial protocols identified were, where available, matched to published articles (Table S5). The titles and abstracts were screened and 131 were deemed to be potentially eligible. However, only 130 full texts were assessed because the full text of 1 RCT was not available (*n* = 1).^27^ During full-text assessment, 76 reports were excluded. The detailed reasons for exclusion for each record are described in Table S6. After full-text screening, 49 eligible RCTs^28–76^ described in 53 reports^28–80^ were included in the present review.

The characteristics of the 49 included RCTs are described in Table 2.^28–76^ A total of 5042 children were analyzed in network meta-analysis, and the average sample size at follow-up was 100.8 participants (range: 31–594). The follow-up at which outcomes were reported ranged from 2 to 24 weeks (median = 4 weeks).

**Table 2.** Characteristics of RCTs Included in the Present Review

**Study (year) [country]**	No. randomized to treatments 1/2/3; age range; and percentage male participants	Diagnosis of functional constipation criteria	Treatment 1, dosage	Treatment 2, dosage	Treatment 3, dosage	**Follow-up** ^a^	Study outcomes	Other co-interventions allowed, and monitored?	Treatment success definition
Voskuijl et al (2004)^28^ [Netherlands]	100 (50/50); 0.5-18 y; 55.0% male	2-4 of the following symptoms for 3 mo: <3 BMs/wk, fecal incontinence >1/wk, large stool mass, palpable rectal mass	PEG powder in sachet, dosage based on age: 0.5-6 y: 2.95 g · d^–1^; >6 y: 5.9 g · d^–1^	Lactulose powder in sachet, dosage based on age: 0.5-6 y: 6 g · d^–1^; >6 y: 12 g · d^–1^	—	**• 8 wk** **•** 26 wk	Primary: **•** Treatment success **•** Defecation frequency **•** Fecal incontinence Secondary: **•** Stool consistency **•** Adverse events	All patients received toilet training advice	≥3 BMs/wk, ≤ 1 FI episode every 2 wk
Banaszkiewicz and Szajewska (2005)^29^ [Poland]	84 (43/41); 2–16 y; % male not reported	<3 BMs/wk for ≥12 wk	Capsules with *L rhamnosus* GG, 2 × 10^9^ CFU/d + lactulose 1 mL · kg^–1^ · d^–1^ (in 2 divided doses)	Lactulose, 1 mL · kg^–1^ · d^–1^ (in 2 divided doses) + placebo	—	**• 4 wk** **•** 8 wk **•** 12 wk	Primary: **•** Treatment success Secondary: **•** Defecation frequency **•** Fecal incontinence **•** Stool consistency **•** Straining **•** Adverse events	All patients received toilet training instructions; whether the use of other laxatives was permitted or not *during* the 12-wk therapy period was not mentioned or reported	≥3 BMs/wk, no FI episodes
Dupont et al (2005)^30^ [France]	98 (53/45); 0.5-3 y; 53.1% male	For children 6-12 mo of age: <1 BM/day; for children 13 mo–3 y of age: <3 BMs/wk	PEG 4000 in sachet, 4 g · d^–1^	Lactulose in sachet, 3.33 g · d^–1^	—	1 mo	**•** Defecation frequency **•** Stool consistency **•** Bloating **•** Flatulence **•** Abdominal pain **•** Vomiting **•** Nausea **•** Laboratory blood tests (protein, serum albumin, serum iron, plasma electrolytes, serum vitamins)	If the maximum dose was not successful, patients could receive up to 2 enemas, 48 h apart. No other laxatives or therapies that could affect gastrointestinal results were allowed. The treatment dose could be reduced if the child produced liquid stools.	Outcome not reported
Urganci et al (2005)^31^ [Turkey]	40 (20/20); 2-12 y; 55.0% male	Rome III	Liquid paraffin, 1 mL · kg^–1^ · d^–1^	Lactulose, 1 mL · kg^–1^ · d^–1^	—	**•** 4 wk **• 8 wk** ^b^	**•** Treatment success **•** Defecation frequency **•** Defecation frequency **•** Optimal dose **•** Compliance rate	All patients received advice about diet (from a nutritionist), daily fiber intake, and toilet training.	>3 BMs/wk + improvement in stool consistency
Loening-Baucke et al (2006)^32^ [USA]	79 (39/40); 4-18 y; 82.3% male	Duration of ≥8 wk and ≥2 of <3 BMs/wk, >1 fecal incontinence episode/wk, large stool mass, withholding behavior	PEG powder, 0.7 · kg^–1^ · d^–1^	MOM, 2 mL · kg^–1^ · d^–1^	—	**• 1 mo** **•** 3 mo **•** 6 mo **•** 12 mo	Primary: **•** Treatment success Secondary: **•** Defecation frequency **•** Fecal incontinence **•** Resolution of abdominal pain **•** Tolerability **•** Adverse events	Parents received written instructions on how to adjust the dosage of PEG or MOM as required.	≥3 BMs/wk + ≤2 FI episodes + no abdominal pain
Bu et al (2007)^33^ [Taiwan]	45 (18/18/9); <10 y; 51.1% male	<3 BMs/wk for >2 mo + ≥1 of the following symptoms: anal fissures with bleeding, FI, or passage of large and hard stool	*L casei rhammosus* (Lcr35) (Anti-biophilus four 250-mg capsules (in 2 divided doses), 8 × 10^8^ CFU/d	MgO, 10 mg · kg^–1^ · d^–1^ (in 2 divided doses)	Placebo	**•** 1 wk **•** 2 wk **•** 3 wk **• 4 wk**	**•** Treatment success **•** Defecation frequency **•** Stool consistency **•** Fecal incontinence **•** Abdominal pain **•** Use of lactulose or glycerin enema **•** Change in appetite **•** Adverse events	All patients asked to discontinue any laxatives before study start, and to avoid any other probiotics, yogurt, or beverages containing probiotics 2 wk before and during therapy. Use of lactulose (if no defecation >3 d) or glycerine enema (if no defecation >5 d) allowed and monitored.	≥3 BMs/wk, without FI episodes in the 4th (last) week
Farahmand (2007)^34^ [Iran]	247 (127/120); 2-12 y; 51.4% male	Rome II	Liquid paraffin at 1-2 mL · kg^–1^ · d^–1^ (in 2 divided doses)	Lactulose 1-2 mL · kg^–1^ · d^–1^ (in 2 divided doses)	—	**•** 4 wk **• 8 wk** ^b^	**•** Defecation frequency **•** FI **•** Treatment success **•** Adverse events	Given instructions to increase their daily fiber intake to an amount of grams equal to their age plus 10.	≥3 BMs/wk + ≤1 FI episode every 2 wk
Thomson et al (2007)^35^ [UK]	51 (27/24); 2-11 y; 43.1% male	Rome III, but must fulfill <3 BMs/wk criterion	PEG powder in sachet, dosage varied based on age and time during intervention^c^	Placebo	—	1 wk	**•** Defecation frequency **•** Fecal incontinence **•** Stool consistency **•** Pain during defecation **•** Straining during defecation **•** Presence of hard stools **•** Abdominal pain during defecation **•** Adverse events	No information given	Outcome not reported
Hannah et al (2008)^36^ [Indonesia]	43 (23/20); 0.5–14 y; 51.2% male	Rome II (adapted)	Placebo, saccharin 500 mg · d^–1^	Synbiotic mixture,^d^ 1 × 10^9^ CFU/d + 5 g FOS	—	1 wk	**•** Treatment success **•** Defecation frequency **•** Stool retention **•** Fecal incontinence **•** Painful defecation **•** Large amount of stool **•** Stool consistency **•** Time to first defecation after treatment initiation **•** Adverse events	Dietary counseling (increase intake of fiber) provided to all patients. Fecal dis-impaction with enema allowed in case of disturbing side effects or no defecation within 1 wk (although none of these occurred).	Reversal of functional constipation diagnosis^e^
Nurko et al (2008)^37^ [USA]	103 (26/24); 4-16 y; 67.0 % male	<3 spontaneous BMs/wk and ≥1 of the following: straining during defecation, hard stools sensation of incomplete evacuation, production of large bowel movements that may obstruct the toilet, or painful defecation	PEG 3350 powder (MiraLax, Braintree Laboratories, Inc; Braintree, MA), 10 mL · kg^–1^ · d^–1^ with maximum 450 mL · d^–1^	Placebo	—	2 wk	**•** Treatment success **•** Defecation frequency **•** Fecal incontinence **•** Stool consistency	All children received toilet training during week 2 of treatment.	≥3 BMs/wk
Karami et al (2009)^38^ [Iran]	103 (48/55); 1-15 y; 51.5% male	Defecation frequency <2/wk, fecal incontinence ≥2/mo, hard fecal consistency and palpable fecal impaction in abdomen or rectum	PEG powder in sachet, 0.8 g · kg^–1^ · d^–1^	Liquid paraffin 1 cc · kg^–1^ · d^–1^ (in 2 divided doses)	—	**•** 1 wk **•** 2 wk **•** 3 wk **• 4 wk** **•** 2 mo **•** 3 mo **•** 4 mo	**•** Defecation frequency **•** Fecal incontinence **•** Pain during defecation **•** Bleeding during defecation **•** Stool consistency	All patients received toilet training and dietary advice.	Outcome not reported
Ratanamongkol et al (2009)^39^ [Thailand]	94 (47/47); 1-4 y; 40.5% male	Rome III	PEG 4000 powder without electrolytes in sachet, 0.5 g · kg^–1^ · d^–1^	MOM, 5 mL · kg^–1^ · d^–1^	—	4 wk	**•** Treatment success **•** Defecation frequency **•** Compliance rate **•** Adverse events	Parents received information on adjusting the dose of the treatment every 3 d as required.	≥3 BMs/wk + ≤ 1 FI episode/mo + no painful defecation
Coccorullo et al (2010)^40^ [Italy]	44 (22/22); >0.5 y; 54.5% male	Rome III	*Lactobacillus reuteri* DSM 17938 in an oil suspension, 1 × 10^8^ CFU/d (in 5 drops)	Placebo	—	8 wk	**•** Defecation frequency **•** Stool consistency **•** Inconsolable crying episodes **•** Treatment success **•** Adverse events	Use of laxatives not allowed during therapy period; glycerine suppository could be used only if no defecation for >5 d. Monitoring unclear (adherence not reported).	≥3 BMs/wk
Khodadad and Sabbaghian (2010)^41^ [Iran]	102 (37/33/32); 4–12 y; 47.4% male	Rome III	Synbiotic mixture (Protexin restore)^d^, 1 × 10^9^ CFU · d^–1^	Synbiotic mixture (Protexin restore),^d^ 1 × 10^9^ CFU · d^–1^, + mineral oil, 1.5 mL· kg^–1^ · d^–1^	Liquid paraffin, 1.5 mL · kg^–1^ · d^–1^ + placebo	4 wk	Primary: **•** Defecation frequency **•** Stool consistency **•** Fecal incontinence **•** Abdominal pain **•** Painful defecation Secondary: **•** Treatment success **•** Adverse events	Dietary and toilet training advice given to all patients. Patients were not allowed to use any other kind of constipation medication during the study period. Monitoring unclear (adherence not reported).	≥3 BMs/wk, ≤2 FI episodes/mo and no abdominal pain
Gomes et al (2011)^42^ [Brazil]	38 (17/21); 1-15 y; 605% male	Rome III	PEG, 0.5 g · kg^–1^ · d^–1^	MOM, 1 mL · kg^–1^ · d^–1^	—	**•** 15 d **• 2 mo** **•** 4 mo **•** 6 mo	**•** Defecation frequency **•** Stool consistency **•** Fecal incontinence **•** Abdominal pain **•** Straining during defecation **•** Tolerability of treatment	Not specified (unclear extent of use of other laxatives, if allowed, and potential differences in these between groups)	Outcome not reported
Guerra et al (2011)^43^ [Brazil]	60 (30/30); 5–15 y; 20.3% male	Rome III	*B longum* in goat yogurt, 1 × 10^9^ CFU · d^–1^ + yogurt starter cultures (*L delbrueckii* subsp *bulgaricus* and *S thermophilus*)	Placebo (goat yogurt, 10 mL without probiotic cultures)	—	10 wk (crossover after 5 wk, no wash-out period)	**•** Defecation frequency **•** Stool consistency **•** Abdominal pain **•** Defecation pain **•** Adverse events	Patients asked to avoid consumption of other fermented dairy products or yogurts during study. Monitoring unclear (adherence not reported, also for other commonly used laxatives).	Outcome not reported
Rafati et al (2011)^44^ [Iran]	160 (80/80); 2-12 y; 53.2% male	≥3 mo with following symptoms: <3 BMs/wk, >1 fecal incontinence episode/wk, palpable fecal mass	PEG 3350 in powder form, 1-1.5 g · kg^–1^ · d^–1^	Liquid paraffin, 1-1.5 g ·kg^–1^ · d^–1^	—	**•** 1 wk **•** 2 wk **• 1 mo** **•** 2 mo **•** 3 mo **•** 4 mo	**•** Defecation frequency **•** Clinical efficacy **•** Adverse events	Diet and toilet training advice given to all patients.	Outcome not reported
Tabbers et al (2011)^45^ [Netherlands and Poland]	159 (79/80); 3–16 y; 52.2% male	Rome III, but all should fulfill defecation frequency <3 BMs/wk criterion	Yogurt (Activia 125-g pot) with *B lactis* DN-173010, ≥4.25 × 10^9^ CFU/pot, 2 pots · d^–1^ *+* 1.2 × 10^9^ CFU yogurt starter cultures (*L delbrueckii* subsp *bulgaricus* and *S thermophilus*)	Placebo (yogurt, 125 g/pot without probiotic cultures, and low lactose content, <2.5 g/pot)	—	3 wk	Primary: **•** Defecation frequency Secondary: **•** Treatment success **•** Response to treatment **•** Stool consistency **•** Fecal incontinence **•** Painful defecation **•** Abdominal pain **•** Flatulence **•** Adverse events	Toilet training advice given to all patients. Intake of any other fermented dairy product was not allowed. Intake of bisacodyl allowed if no defecation >3 d (monitored and reported).	≥3 BMs/wk, without FI episodes during last 2 wk of treatment
Saneian and Mostofizadeh (2012)^46^ [Iran]	75 (25/25/25); 1-6 y; % male not reported	Rome III	PEG (School of Pharmacy, Shiraz University of Medical Sciences, Shiraz, Iran), 1 cc · kg^–1^ · d^–1^	Lactulose (Tolid Daroo, Tehran, Iran), 1 cc · kg^–1^ · d^–1^	MOM (Tolid Daroo, Tehran), 1 cc · kg^–1^ · d^–1^	**• 1 wk** **•** Monthly for 4-6 mo	**•** Treatment success **•** Defecation frequency	Not specified (unclear extent of use of other laxatives, if allowed, and potential differences in these between groups)	>3 BMs/wk without pain and bleeding, + <2 FI episodes/mo
Wang et al (2012)^47^ [China]	216 (105/111); 8-18 y; 41.7% male	<3 BMs/wk, and stool consistency type 1-3 (Bristol Stool Scale) for at least 2 wk	PEG 4000, 20 g · d^–1^	Lactulose (Duphalac), 10 g · d^–1^ for first 3 d and 6.7 g · d^–1^ for the next 11 d	—	2 wk	Primary: **•** Defecation frequency **•** Stool consistency Secondary: **•** Treatment success **•** Abdominal pain	Not specified (unclear extent of use of other laxatives, if allowed, and potential differences in these between groups)	>3 BMs/wk + 4-6 rating on BSFS
Olgaç et al (2013)^48^ [Turkey]	53 (25/28); 4-16 y; % male not reported	Rome III	Lactobacillus reuteri, 1 · 10^8^ CFU · d^–1^	Lactulose**,** 1 mL · 10^8^ · kg · d^–1^	—	4 wk	**•** Defecation frequency **•** Stool consistency **•** Rectal bleeding **•** Abdominal pain **•** Flatulence	Magnesium hydroxide used as rescue therapy in absence of defecation from 3 consecutive days.	>3 BMs/wk, 4-6 rating on BSFS, and no difficulty during defecation, abdominal pain, stool retention, rectal bleeding, and stool incontinence
Mugie et al (2014)^49^ [33 medical centers in Europe: Netherlands, Poland, Hungary, UK, Belgium]	215 (107/108); 0.5-18 y; 44.6% male	Rome III	Prucalopride succinate oral solution, 0.04 mg · kg^–1^ · d^–1^ for patients ≤50 kg Prucalopride tablet, 2 mg · kg^–1^ · d^–1^, for patients > 50 kg	Placebo	—	8 wk	**•** Defecation frequency **•** Fecal incontinence **•** Stool consistency	Other laxatives were not allowed.	≥3 BMs/wk + ≤ 1 FI episode every 2 wk during weeks 5-8 of the study
Sadeghzadeh et al (2014)^50^ [Iran]	56 (28/28); 4–12 y; 50.0% male	Rome III	Probiotic mixture (Protexin),^d^ 1 × 10^9^ CFU/d + lactulose, 1 mL · kg^–1^ · d^–1^	Lactulose, 1 mL · kg^–1^ · d^–1^ + placebo	—	4 wk	**•** Defecation frequency **•** Stool consistency **•** Abdominal pain **•** Fecal incontinence **•** Weight gain **•** Adverse events	Not specified (unclear extent of use of other laxatives, if allowed, and potential differences in these between groups)	Outcome not reported
Treepongkaruna et al (2014)^51^ [Thailand]	88 (44/44); 1-3 y; 56.8% male	Rome II, modified^f^	PEG 4000 (Forlax sachet), 8 g · d^–1^	Lactulose, 3.3 g · d^–1^	—	4 wk	**•** Defecation frequency **•** Stool consistency **•** Ease of stool passage **•** Occurrence of cramping, flatulence, anal irritation **•** Adverse events	All patients received dietary advice.	Outcome not reported
Ala et al (2015)^52^ [Iran]	200 (100/100); 1-12 y; 51.5% male	Rome III	PEG at 0.7 g · kg^–1^ · d^–1^ (in 2 divided doses)	PEG at 0.7 g · kg^–1^ · d^–1^ + lactulose at 3 cc · kg^–1^ · d^–1^ (in 2 divided doses)	—	**•** 1 mo **•** 3 mo **•** 6 mo **•** 12 mo	**•** Treatment success	Dietary advice and toilet training was provided to patients through face-to-face consultation and pamphlets	≥3 BMs/wk + ≤ 2 FI/mo + no abdominal pain
Hashemi et al (2015)^53^ [Iran]	120 (40/40*/*40); 2–16 y; % male not reported	Rome III	Synbiotic mixture (Kidilact sachet),^d^ dosages not specified + PEG, 0.7 g · kg^–1^ · d^–1^	Synbiotic mixture (Kidilact sachet),^d^ dosages not specified	PEG, 0.7 g · kg^–1^ · d^–1^ + placebo	6 wk	**•** Defecation frequency **•** Stool consistency **•** Abdominal pain **•** Painful defecation **•** Adverse events	Not specified (unclear extent of use of other laxatives, if allowed, and potential differences in these between groups)	Outcome not reported
Abediny et al (2016)^54^ [Iran]	90 (45/45); 4–12 y; % male not reported	Rome III	Synbiotic mixture (Kidilact sachet),^d^ 1–2 sachets · d^–1^ dosage not specified + PEG (Pidrolax), 0.7–1.5 g · kg^–1^ · d^–1^	PEG (Pidrolax), 0.7–1.5 g · kg^–1^ · d^–1^	—	4 wk	Primary: **•** Defecation frequency **•** Stool consistency **•** Fecal incontinence **•** Abdominal pain **•** Painful defecation Secondary: **•** Treatment success **•** Adverse events	Not specified (unclear extent of use of other laxatives, if allowed, and potential differences in these between groups)	Outcome not reported
Baştürk et al (2017)^55^ [Turkey]	155 (77/78); 4–18 y; 45.8% male	Rome III, but all should fulfill defecation frequency of <3/wk criterion	Synbiotic mixture**,**^g^ 4 × 10^9^ CFU/d + 1996.57 mg prebiotics	Placebo	—	4 wk	Primary: **•** “Complete” benefit Secondary: **•** Defecation frequency **•** Stool consistency **•** Fecal incontinence **•** Abdominal pain **•** Painful defecation **•** Rectal bleeding **•** Withholding behavior **•** Adverse events	Dietary advice (fiber intake) and toilet training given to all participants. Unclear whether the use of other laxatives was not allowed. Fleet enema was performed in patients who presented with complaints of progressive abdominal distention and pain while on treatment—this was monitored and reported.	≥3 BMs/wk, softening in the stool consistency (BSFS ≥4), ≤1 FI
Wojtyniak et al (2017)^56^ [Poland]	94 (48/46); <5 y; 44.7% male	Rome III for ≥1 mo if aged <4 y or 2 mo if aged >4 y	*L casei rhamnosus* Lcr35 in capsule with powder, 8 × 10^8^ CFU, twice daily	Placebo, 99% milk powder and 1% magnesium stearate	—	**•** 1 wk **•** 2 wk **•** 3 wk **• 4 wk**	Primary: **•** Treatment success Secondary: **•** Stool consistency **•** Defecation frequency **•** Fecal incontinence **•** Painful defecation **•** Abdominal pain **•** Flatulence **•** Need for intake of other laxatives **•** Adverse events	Patients were asked to discontinue any laxatives before start of treatment. Use of PEG allowed when no defecation for 3 d (monitored and reported).	≥3 BMs/wk, without FI episodes in the 4th (last) wk
Mahdavi et al (2017)^57^ [Iran]	79 (38/41); 2–10 y; 46.8% male	Rome III	Synbiotic mixture (Kidilact sachet),^d^ 1 × 10^9^ CFU · d^–1^ + PEG 4000 in sachet (Pidrolax), 0.6 g · kg^–1^ · d^–1^	PEG 4000 in sachet (Pidrolax), 0.6 g · kg^–1^ · d^–1^	—	**• 4 wk** **•** 8 wk **•** 12 wk	Primary: **•** Defecation frequency **•** Fecal incontinence **•** Withholding behavior **•** Stool consistency **•** Painful defecation **•** Drug acceptance **•** Adverse events Secondary: **•** Continued use of anti-constipation drugs >1/wk after 8 wk	No details provided. Clearly, use of other drugs such as antibiotics and antihistamines occurred but patients who took these were excluded from the analyses.	Outcome not reported
Russo et al (2017)^58^ [Italy]	55 (27/28); 4–12 y; 47.3% male	Rome III	Probiotic mixture (Tribif sachet),^h^ 3.5 × 10^8^ CFU + PEG 4000 in sachets (Perdigal), 0.4 g · kg^–1^ · d^–1^; up to 0.8 g · kg^–1^ · d^–1^ if no improvement observed after 3 d	PEG 4000 in sachets (Perdigal), 0.4 g · kg^–1^ · d^–1^; up to 0.8 g · kg^–1^ · d^–1^ if no improvement observed after 3 d	—	**•** 2 wk **• 4 wk** **•** 8 wk **•** 12 wk	Primary: **•** Defecation frequency **•** Stool consistency **•** Abdominal pain **•** Fecal incontinence **•** Painful defecation **•** Rectal bleeding **•** Treatment success Secondary: **•** Adverse events	Toilet advice given to all patients. The use of other laxatives was not allowed during therapy period, but enemas were permitted when no defecation for >3 d. Monitored and reported.	≥3 BMs/wk, stool consistency ≥grade 3 on BSFS, and no episodes of abdominal pain, FI, painful defecation, or rectal bleeding
Torabi et al (2017)^59^ [Iran]	160 (80/80); 2-12 y; 43.1% male	Rome III	PEG (Pidrolax liquid solution), 1 cc · kg^–1^ · d^–1^ (in 2 divided doses)	Liquid paraffin, 1 cc · kg^–1^ · d^–1^ (in 2 divided doses)	—	**•** 1 wk **•** 2 wk **•** 3 wk **• 4 wk** **•** Monthly from 2 to 6 mo	**•** Treatment success **•** Defecation frequency **•** Stool consistency **•** Fecal incontinence **•** Pain during defecation **•** Abdominal pain **•** Rectal bleeding **•** Adverse events	Not specified (unclear extent of use of other laxatives, if allowed, and potential differences in these between groups)	≥3 BMs/wk, FI <1/d, absence of pain during defecation and stony or bloody stools^i^
Cao and Liu (2018)^60^ [China]	100 (50/50); 2-6 y; 54.0% male	Rome III	Lactulose at 3.3 g · d^–1^	Placebo	—	6 wk	Primary: **•** Defecation frequency Secondary: **•** Stool consistency **•** Abdominal pain **•** Flatulence **•** Adverse events	No details provided	Outcome not reported
Wegner et al (2018)^61^ [Poland]	129 (65/64); 3–7 y; 55.8% male	Rome III	*L reuteri* DSM 17938 in tablet form, 1 × 10^8^ CFU · d^–1^*+* PEG (macrogol), 10 g · d^–1^ or higher if defecation <3 BMs/wk	PEG (macrogol), 10 g/d or higher if defecation <3 BMs/wk + placebo	—	**• 4 wk** **•** 8 wk	Primary: **•** Treatment success Secondary: **•** Defecation frequency **•** Stool consistency **•** Painful defecation **•** Fecal incontinence **•** Abdominal pain **•** Withholding behavior **•** Adverse events	Dose of PEG increased when <3 BMs/wk; rescue medication (enema) allowed only after 5 d without any BMs. Monitored and reported.	≥3 BMs/wk
Jose and Ismael (2018)^62^ [India]	60 (30/30); <18 y; 50.0% male	Rome III or clinical symptoms	Probiotic mixture (Protexin),^j^ dosage unspecified + lactulose, dosage unspecified	Lactulose, dosage unspecified + placebo	—	4 wk	**•** Defecation frequency **•** Stool consistency **•** Abdominal pain **•** Fecal incontinence **•** Weight gain **•** Adverse events	Not specified (unclear extent of use of other laxatives, if allowed, and potential differences in these between groups)	Outcome not reported
Jadrešin et al (2018)^63^ [Croatia]	33 (18/15); 2–18 y; 30.3% male	Rome III	*L reuteri* DSM 17938 in chewable tablet (450 mg), 1 × 10^8^ CFU · d^–1^ + lactulose, 1–3 mL · kg^–1^ · d^–1^	Lactulose, 1–3 mL · kg^–1^ · d^–1^ + placebo	—	12 wk	Primary: **•** Defecation frequency **•** Resolution of symptoms at the end of study Secondary: **•** Need of lactulose treatment at end of study **•** Dose of lactulose used **•** Fecal incontinence **•** Stool consistency **•** Abdominal pain **•** Adverse events	Consumption of other prebiotic or probiotic products was not allowed during the intervention period. Monitoring unclear (adherence not reported, also not for other laxatives).	Resolution of symptoms (ie, daily defecation and absence of FI and pain)
Modin et al (2018)^64^ [Denmark]	115 (58/57); 2-16 y; 55.9% male	Rome III	PEG in powder sachet, 0.8 g · kg^–1^ · d^–1^	Placebo	—	24 wk	Primary: **•** Treatment success Secondary: **•** Defecation frequency **•** Fecal incontinence **•** Abdominal pain **•** Need for rescue medication	No other laxatives were allowed	Absence of any Rome III criteria
Cassettari et al (2019)^65^ [Brazil]	80 (16/17); 5-15 y; 53.8% male	Rome IV	PEG 3350 with electrolytes, dosage not specified	Sodium picosulfate, dosage not specified	—	8 wk	**•** Treatment success **•** Stool consistency **•** Fecal incontinence **•** Straining during defecation **•** Pain during defecation **•** Blood in stool **•** Abdominal pain **•** Decreased laxative doses	All patients received toilet training advice.	≥3 BMs/wk
Jarzebicka et al (2019)^66^ [Poland]	102 (51/51); 0.5-6 y; 55.9% male	Rome III	PEG powder, based on participants body weight: ≤8 kg, 5 g · d^–1^ 8-12 kg, 10 g · d^–1^ 12-20 kg, 15 g · d^–1^ ≥20 kg, 20 g · d^–1^	Lactulose administered in liquid form, 2 mL · kg^–1^ · d^–1^	—	**•** 4 wk **•** 12 wk	**•** Treatment success **•** Defecation frequency **•** Pain during defecation **•** Hard stool **•** Passage of large fecal mass **•** Side effects	Parents were instructed on how to perform daily defecation training.	≥3 BMs/wk, improvement of ≥2 on BSFS
Kubota et al (2020)^67^ [Japan]	63 (21/20/22); 0.5-6 y; 55.0% male	Rome IV	*Lactobacillus reuteri* DSM 17938 in oil suspension, 1 × 10^8^ CFU · d^–1^ (in 10 drops) + lactose hydrate (a placebo of MgO)	*Lactobacillus reuteri* DSM 17938 in oil suspension, 1 × 10^8^ CFU · d^–1^ (in 10 drops) + MgO, 30 mg · kg^–1^ · d^–1^	**MgO**, 30 mg · kg^–1^ · d^–1^ + **lactose hydrate** (a placebo of MgO)	**•** 2 wk **• 4 wk**	**•** Defecation frequency **•** Stool consistency **•** Shannon index of gut microbiome diversity	Other treatments including laxatives, probiotics, antibiotics, fermented dairy products, and yogurt were not allowed. Glycerine suppositories were used when there was no defecation for >3 d.	Outcome not reported
Tjokronegoro et al (2020)^68^ [Indonesia]	78 (39/39); 4-10 y; 35.9% male	Rome III	Probiotic mixture^k^ (LactoB sachet), 1 · 10^9^ CFU	Placebo containing maltodextrin	—	4 wk	**•** Defecation frequency **•** Treatment success **•** Stool consistency **•** Pain during defecation **•** Fecal incontinence **•** Withholding behavior **•** Stool mass in abdomen	No other laxative or probiotic products were allowed.	A decreased constipation severity score >60% at the end of evaluation
Dheivamani et al (2021)^69^ [India]	100 (50/50); 2-12 y; 51.0% male	Rome IV	PEG 3350 in powder form at 0.7 g · kg^–1^ · d^–1^	Lactulose, 0.7 g · kg^–1^ · d^–1^	—	4 wk	Primary: **•** Defecation frequency **•** Frequency of painful bowel movements **•** Passage of large fecal mass Secondary: **•** Straining during defecation **•** Adverse events **•** Tolerability of treatment	Not specified (unclear extent of use of other laxatives, if allowed, and potential differences in these between groups)	Outcome not reported
Worona-Dibner et al (2021)^70^ [Mexico]	83 (41/42); 0.5-18 y; 47.0% male	Rome III	PEG 3350 (Contumax sachet), 0.7-2.1 g · kg^–1^ · d^–1^ Dosage increased by 5 g every third day until achieving 1-3 BMs/d	MOM (Normex), 2-6 mL · kg^–1^ · d^–1^ Dosage increased by 5 g every third day until achieving 1-3 BMs/d	—	12 mo	Primary: **•** Treatment success **•** No. of laxative doses rejected **•** Adverse events Secondary: **•** Defecation frequency **•** Fecal incontinence **•** Fecal impaction episodes **•** Laxative dose required **•** Duration of treatment required for treatment success	All patients received toilet training advice.	≥3 BMs/wk, no FI episodes, fecal impaction, abdominal pain, or need for another laxative
Benninga et al (2022)^71^ [USA, Canada]	606 (404/202); 6-17 y; 45.3% male	Rome III	Lubiprostone 12 · d^–1^ or 24 mg d^–1^ (in 2 divided doses) Dosage depended on child weight^l^	Placebo	—	12 wk	Primary: **•** Treatment success Secondary: **•** Adverse events (headache, nausea, vomiting, abdominal pain, nasopharyngitis, diarrhea)	Rescue medications were used when there was no defecation for >3 d.	Increase of ≥1 BM/wk, ≥3 BMs/wk for 9 wk
Foroughi et al (2022)^72^ [Iran]	72 (36/36); 2-12 y; 55.1% male	Rome IV	PEG, 6 g · d^–1^ **+ **probiotic mixture,^m^ 1 · 10^9^ CFU Combined in a powder in sachet (Tak Zhen Zist Co, Iran)	PEG, 6 g · d^–1^, in powder in sachet (Tak Zhen Zist Co, Iran)	—	**3 wk**	**•** Defecation frequency **•** Number of painless BMs	Diet and toilet training advice given to all patients.	Outcome not reported
Gan et al (2022)^73^ [China]	100 (47/45)^n^; 4-12 y; 50.0% male	Rome III	Probiotic mixture^o^ in a chewable tablet, 5.10^9^ CFU · d^–1^	Placebo, chewable tablet	—	4 wk	**•** Defecation frequency **•** Stool consistency **•** Microbial diversity (alpha diversity, beta diversity and microbial community analysis)	Not specified (unclear extent of use of other laxatives, if allowed, and potential differences in these between groups)	Outcome not reported
Lee et al (2022)^74^ [South Korea]	187 (50/68/69); 0.5-10 y; 46.0% male	Rome IV	*Saccharomyces boulardii* (Bioflor 250 sachet) 5 · 10^9^ CFU · d^–1^ The number of sachets for patient age was: ≤2 y, 2 sachets · d^–1^; >2 y, 3 sachets · d^–1^	*Saccharomyces boulardii* (Bioflor 250) of 5 · 10^9^ CFU · d^–1^ + syrup containing 1.34 g · mL^–1^ of lactulose (Duphalac)	Lactulose (Duphalac), 1.34 g · mL^–1^ · d^–1^	**• 2 wk** **• 6 wk** ^p^ **•** 12 wk	Primary: **•** Treatment success Secondary: **•** Defecation frequency **•** Stool consistency **•** Fecal incontinence **•** Painful defecation **•** Drug changes	No other laxatives or probiotics were allowed.	≥3 BMs/wk, no FI episodes in toilet-trained children
Mansour et al (2022)^75^ [Syria]	43 (21/22); 1-13 y; 46.5% male	Rome IV	PEG 3350, 0.8 g · kg^–1^ · d^–1^ in 2 divided doses	Lactulose, 2 mL · kg^–1^ · d^–1^	—	**• 4 wk** **•** 8 wk **• 12 wk** ^q^ **•** 16 wk **•** 20 wk	**•** Treatment success **•** Defecation frequency **•** Painful defecation **•** Fecal incontinence **•** Hard stool	Not specified (unclear extent of use of other laxatives, if allowed, and potential differences in these between groups)	≥3 BMs/wk, ≤1 FI episodes every 2 wk, improvement in stool consistency without painful defecation
Lojanatorn et al (2023)^76^ [Thailand]	50 (21/18)^r^; 1-5 y; 82.3% male	Rome IV	*B clausii*, 2 ·10^9^ spores· d^–1^ administered in 5-mL liquid suspension	Placebo, 5 mL water · d^–1^	—	**•** 2 wk **• 4 wk**	Primary: **•** Treatment success Secondary: **•** Defecation frequency **•** Stool consistency **•** Rectal enema use **•** Adverse effects	Sodium chloride enema if ≥3 d with no defecation.	≥3 BMs/wk + stool consistency ≥3 on BSFS

*Abbreviations:*  *B*, *Bacillus*; BM, bowel movement; BSFS, Bristol Stool Form Scale; CFU, colony-forming units; FI, fecal incontinence; FU, follow-up; *L*, *Lactobacillus*; MgO, magnesium oxide; MOM, magnesium hydroxide; PEG, polyethylene glycol; RCT, randomized controlled trial; *S*, *Streptococcus*.

a Follow-up points presented in bold are those from which data were extracted and used in meta-analysis.

b BMs/wk were reported in the paper as an average of the first 4 wk at the 4-wk FU and as an average of the last 4 wk at the 8-wk FU. The 8-wk FU was selected for use in meta-analysis because using data from less than 1 wk after treatment is less similar to the follow-up time points of other studies.

c For children aged 2-6 y, children took 1 sachet of PEG+Electrolytes per day for days 1-2 and then increased by 1 sachet every 2 d until day 8. For children aged 7-11 years, children took 2 sachets of PEG+Electrolytes per day for the first 4 d, 5 sachets per day for days 5-6, and 6 sachets per day for days 7-8.

d Combination of 7 probiotic strains (*L acidophilus*, *L casei*, *L rhamnosus*, *L bulgaricus*, *B breve*, *B infantis*, *S thermophilus*) and fructooligosaccharides as prebiotic.

e That is, no longer meeting the following criteria used for the diagnosis: <3 BMs/wk with no anatomic, endocrine, or metabolic disorder, or painful defecation and fecal retention despite ≥3 BMs/wk (if aged ≤4 y); any 2 of the following: <3 BMs/wk without laxatives; ≥2 FI episodes/wk; periodic passage of very large stool once every 7–30 d; and a palpable abdominal or rectal mass on physical examination (if aged >4 y).

f Either stool frequency ≤2/wk for ≥3 mo, or the presence of pebble-like hard stools, painful defecation or FI for ≥3 mo.

g Combination of *L casei*, *L rhamnosus*, *L plantarum*, *B lactis*, and fiber, polydextrose, FOS, and galacto-oligosaccharides as prebiotics.

h Combination of *B breve*, *B infantis*, and *B longum*.

i This RCT reported ‘poor response’ as <3 BMs/wk, or painful defecation, stony and bloody stools, or daily soiling and incontinence. Patients were considered to be ‘successful treated’ if they did not have a poor response.

j Combination of 7 probiotic strains (*L acidophilus*, *L casei*, *L rhamnosus*, *L bulgaricus*, *B breve*, *B infantis*, *S thermophilus*).

k *L acidophilus*, *B longum*, *S thermophilus.*

l Children weighing <50 kg were allocated to 12 mg · d^−1^. Children weighing ≥50 kg were allocated to 24 mg · d^−1^.

m Combination of *L reuteri*, *L rhamnosus*, and *Bifidobacterium infantis.*

n The number of participants in each group after randomization was not reported. The numbers reported and shown here are the number of participants in each group at follow-up.

o Combination of *L acidophilus* DDS-1R and *Bifidobacterium animalis* subsp. lactis UABla-12TM.

p Different outcomes were reported at different time points. Treatment success was only reported at 6 wk; therefore, data used in meta-analysis of different outcomes were taken from 2 different time points.

q Different outcomes were reported at different time points. Treatment success was only reported at 12 wk; therefore, data used in meta-analysis of different outcomes were taken from 2 different time points.

r The number of participants in each group after randomization was not reported. The numbers reported and shown here are the number of participants in each group after the 2-wk washout period before the intervention began.

Most trials defined FC according to the Rome III^31^^,^^35^^,^^39–43^^,^^45^^,^^46^^,^^48–50^^,^^52–64^^,^^66^^,^^68^^,^^70^^,^^71^^,^^73^ or the Rome II^34^^,^^36^^,^^51^ criteria. Seven of the 12 trials published after 2019 to the present used the updated Rome IV criteria.^65^^,^^67^^,^^69^^,^^72^^,^^74–76^ There were 9 trials that did not use the any of the Rome criteria but used specified definitions, which were deemed to be comparable enough for inclusion.^28–30^^,^^32^^,^^33^^,^^37^^,^^38^^,^^44^^,^^47^ There was a median of 2.2 BMs per week at baseline across the trials.

Polyethylene glycol was the most studied treatment, being evaluated by 24 trials and 1194 children were randomized to this treatment. An additional 315 children were randomized to a combination therapy of PEG and probiotics (*n* = 215)^53^^,^^54^^,^^57^^,^^58^^,^^61^ or PEG and lactulose (*n* = 100).^52^

### Assessment of transitivity

Assessments of study characteristics are shown in Figures S1 and S2 for the outcomes of defecation frequency and treatment success, respectively. There was no clear violation of the transitivity assumption when comparing the characteristics of the studies across interventions.

### Results from network meta-analysis for the outcome of defecation frequency

**Figure 2.**
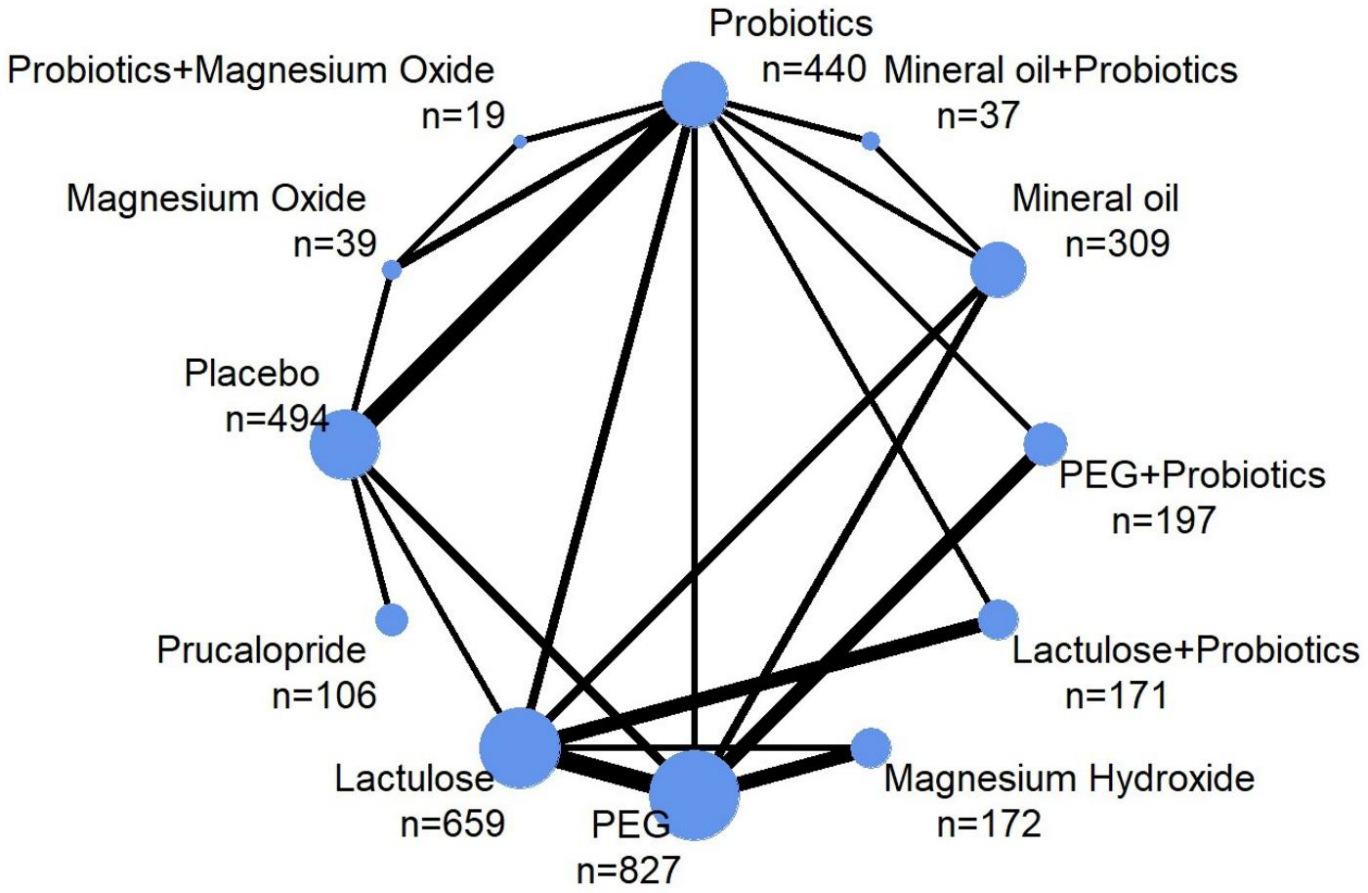
Network Plot Showing Direct Comparisons Between Treatments for the Outcome of Defecation Frequency. The thickness of lines corresponds to the number of trials comparing the linked treatments. The sizes of the nodes (blue circles) correspond to the number of participants randomized to each treatment. Abbreviation: PEG, polyethylene glycol

There were 41 RCTs of 12 different intervention arms (various combinations of 8 different active treatment components + placebo), which provided data required for meta-analysis for the outcome of defecation frequency (Figure 2). There were 17 different comparison designs from direct evidence of 53 pairwise comparisons of these interventions. A total of 3470 children with FC were included in the analysis.

### Comparison of probiotics to conventional treatments

**Figure 3.**
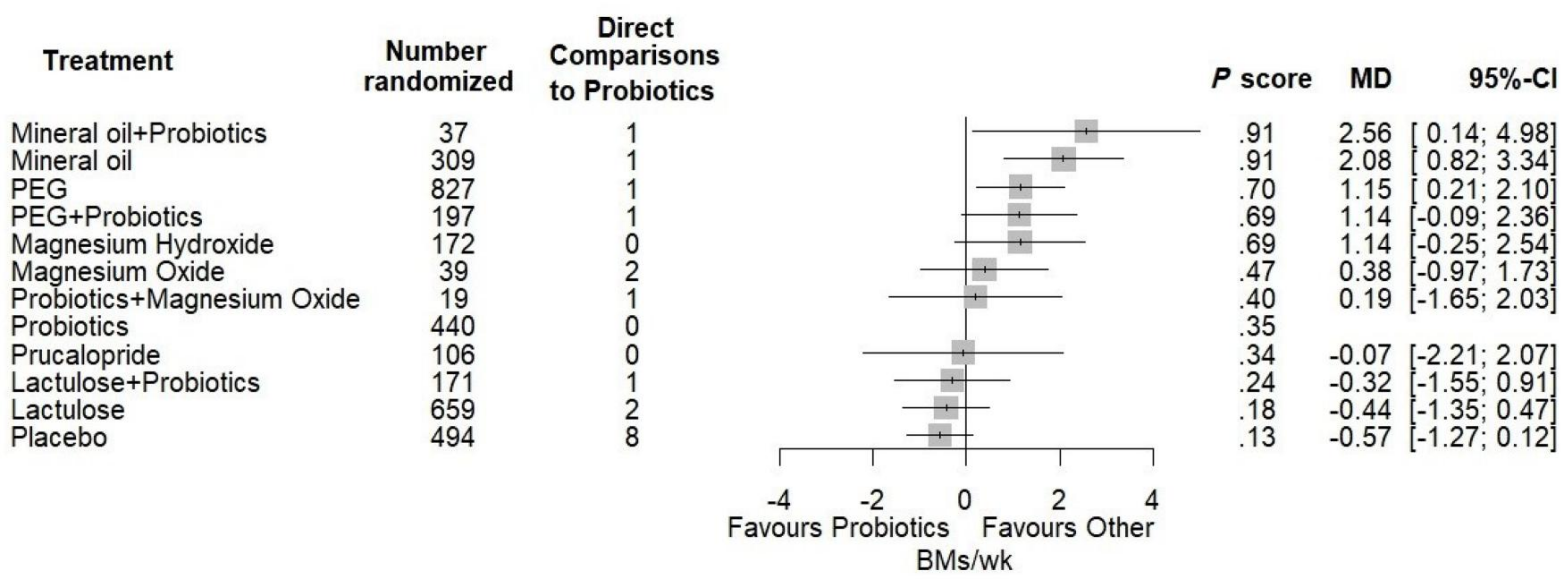
Forest Plot Depicting the Treatment Effects of Conventional Treatments and Placebo as Compared With Probiotics From Random-Effects Network Meta-analysis of Defecation Frequency. Pooled effect estimates (squares) with 95% CIs are shown for standard network meta-analysis. Treatments are ordered from highest to lowest *P* scores (probability of being the most effective treatment) from the standard network meta-analysis model. Abbreviations: BM, bowel movement; CI, confidence interval; MD, mean difference; PEG, polyethylene glycol

Figure 3 shows the comparisons of probiotics to all other treatments and placebo from the network meta-analysis of defecation frequency, ordered by the probability of being the most effective at increasing BMs per week. Probiotics did not statistically significantly increase the number of BMs/wk when compared with any conventional treatment for FC and was ranked as eighth most effective out of all 12 treatments (including placebo) based on the probability of being the most effective at increasing defecation frequency (*P* = .35). A combined treatment of mineral oil and probiotics increased BMs/wk by 2.56 (95% CI: 0.14, 4.98) as compared with probiotics as a standalone treatment and was ranked as having the highest probability of being the most effective treatment (*P* = .909). Mineral oil alone also statistically significantly increased the number of BMs per week (2.65; 95% CI: 1.31, 3.99) and was ranked as having the second highest probability of being the most effective treatment (*P* = .906).

### Conventional treatments and probiotics as compared with placebo

The effect estimates for the comparisons between all treatments in the network meta-analysis of defecation frequency, including the comparisons between conventional treatments, probiotics, and placebo, are shown in the league table (Table 3). When compared with placebo, a combined treatment of mineral oil and probiotics increased BMs/wk by 3.13 (95% CI: 0.63, 5.62) Mineral oil alone also statistically significantly increased the number of BMs/wk (2.65; 95% CI: 1.31, 3.98), followed by PEG (1.72; 95% CI: 0.72, 2.73), PEG combined with probiotics (1.71; 95% CI: 0.42, 3.00), and magnesium hydroxide (1.72; 95% CI: 0.27, 3.16). All other treatments did not show a statistically significant effect on BMs/wk and effect sizes were smaller than required to be considered clinically relevant. Notably, probiotics as a standalone treatment had a small effect on BMs per week (0.57), which was not significant (95% CI: –0.12, 1.27). When an additive component network meta-analysis model was conducted, there were no changes in the statistical significance of any treatment effects. However, the effect of probiotics as a standalone treatment was decreased to 0.27 BMs/wk (–0.19, 0.73) and mineral oil became slightly less effective as a combined treatment with probiotics (2.70; 95% CI: 1.34, 4.05).

**Table 3.** League Table for the Outcome of Defecation Frequency (Bowel Movements/Week)

Mineral oil + probiotics	0.74 [−1.85; 3.33]^b^						2.27 [−0.39; 4.93]^b^				
0.48 [−1.91; 2.88]^b^	Mineral oil	−0.43 [−1.95; 1.09]^b^					1.53 [−0.91; 3.97]^b^			4.71 [2.91; 6.50]^b^	
1.41 [−1.05; 3.87]^b^	0.93 [−0.18; 2.03]^b^	PEG	−0.08 [−1.03; 0.86]^c^	−0.10 [−1.21; 1.01]^b^			0.08 [−1.93; 2.09]^b^			1.28 [0.43; 2.13]^b^	1.74 [−0.11; 3.59]^b^
1.43 [−1.18; 4.03]^b^	0.94 [−0.47; 2.36]^b^	0.02 [−0.90; 0.94]^b^	PEG + probiotics				0.83 [−1.20; 2.86]^b^				
1.42 [−1.26; 4.09]^b^	0.93 [−0.59; 2.45]^b^	0.01 [−1.07; 1.08]^b^	−0.01 [−1.42; 1.40]^b^	Magnesium hydroxide						1.51 [−0.69; 3.71]^b^	
2.18 [−0.58; 4.94]^b^	1.70 [−0.13; 3.52]^b^	0.77 [−0.84; 2.38]^b^	0.75 [−1.04; 2.55]^b^	0.76 [−1.15; 2.68]^b^	Magnesium oxide	0.00 [−1.98; 1.98]^b^	−0.13 [−1.58; 1.32]^c^				2.15 [0.08; 4.22]^b^
2.37 [−0.67; 5.40]^b^	1.89 [−0.34; 4.11]^b^	0.96 [−1.09; 3.02]^b^	0.94 [−1.26; 3.15]^b^	0.95 [−1.35; 3.25]^b^	0.19 [−1.65; 2.03]^b^	Probiotics + magnesium oxide	−0.00 [−1.98; 1.98]^b^				
**2.56 [0.14; 4.98]** ^b^	**2.08 [0.82; 3.34]** ^b^	**1.15 [0.21; 2.10]** ^b^	1.14 [−0.09; 2.36]^b^	1.14 [−0.25; 2.54]^b^	0.38 [−0.97; 1.73]^b^	0.19 [−1.65; 2.03]^b^	Probiotics		−0.88 [−3.12; 1.36]^b^	0.07 [−1.40; 1.53]^b^	0.51 [−0.26; 1.28]^a^
2.63 [−0.57; 5.84]^b^	2.15 [−0.28; 4.58]^b^	1.22 [−1.04; 3.49]^b^	1.21 [−1.20; 3.61]^b^	1.22 [−1.27; 3.70]^b^	0.45 [−2.02; 2.93]^b^	0.26 [−2.54; 3.06]^b^	0.07 [−2.07; 2.21]^b^	Prucalopride			0.50 [−1.53; 2.53]^a^
**2.88 [0.27; 5.49]** ^b^	**2.39 [0.95; 3.84]** ^b^	**1.47 [0.32; 2.62]** ^b^	**1.45 [0.01; 2.89]** ^b^	1.46 [−0.07; 2.99]^b^	0.70 [−1.10; 2.50]^b^	0.51 [−1.70; 2.71]^b^	0.32 [−0.91; 1.55]^b^	0.24 [−2.16; 2.65]^b^	Lactulose + probiotics	0.01 [−0.94; 0.96]^b^	
**3.00 [0.54; 5.46]** ^b^	**2.52 [1.39; 3.65]** ^b^	**1.59 [0.88; 2.31]** ^b^	**1.57 [0.44; 2.71]** ^b^	**1.58 [0.36; 2.81]** ^b^	0.82 [−0.78; 2.42]^b^	0.63 [−1.41; 2.67]^b^	0.44 [−0.47; 1.35]^b^	0.37 [−1.89; 2.62]^b^	0.12 [−0.81; 1.05]^b^	Lactulose	0.30 [−1.68; 2.28]^a^
**3.13 [0.65; 5.62]** ^b^	**2.65 [1.31; 3.98]** ^b^	**1.72 [0.72; 2.73]** ^b^	**1.71 [0.42; 3.00]** ^b^	**1.72 [0.27; 3.16]** ^b^	0.95 [−0.47; 2.38]^b^	0.76 [−1.17; 2.70]^b^	0.57 [−0.12; 1.27]^b^	0.50 [−1.53; 2.53]^b^	0.26 [−1.05; 1.56]^b^	0.13 [−0.85; 1.12]^b^	Placebo

*Abbreviations*: CINeMA, Confidence in Network Meta-Analysis; PEG, polyethylene glycol.

Treatment effects shown are mean differences and 95% CIs and based on direct evidence from pairwise meta-analyses (in the effect sizes above treatment names) and mixed evidence from network meta-analyses (in the effect sizes below treatment names). Blank cells in the effect sizes above treatment names indicate that no direct evidence exists for that comparison. Treatments are ordered from lowest to highest to lowest probability of being the most effective at increasing defecation frequency from left to right. Evidence was rated as moderate certainty (all cells in blue), low certainty (all cells in yellow), or very low certainty (all cells in red) in accordance with the CINeMA framework. Statistically significant treatments effects are shown in bold.

a There is moderate certainty in the evidence of these effect sizes according to assessments from the CINeMA Framework.

b There is low certainty in the evidence of these effect sizes according to assessments from the CINeMA Framework.

c There is very low certainty in the evidence of these effect sizes according to assessments from the CINeMA Framework.

### Results from network meta-analysis for the outcome of treatment success

**Figure 4.**
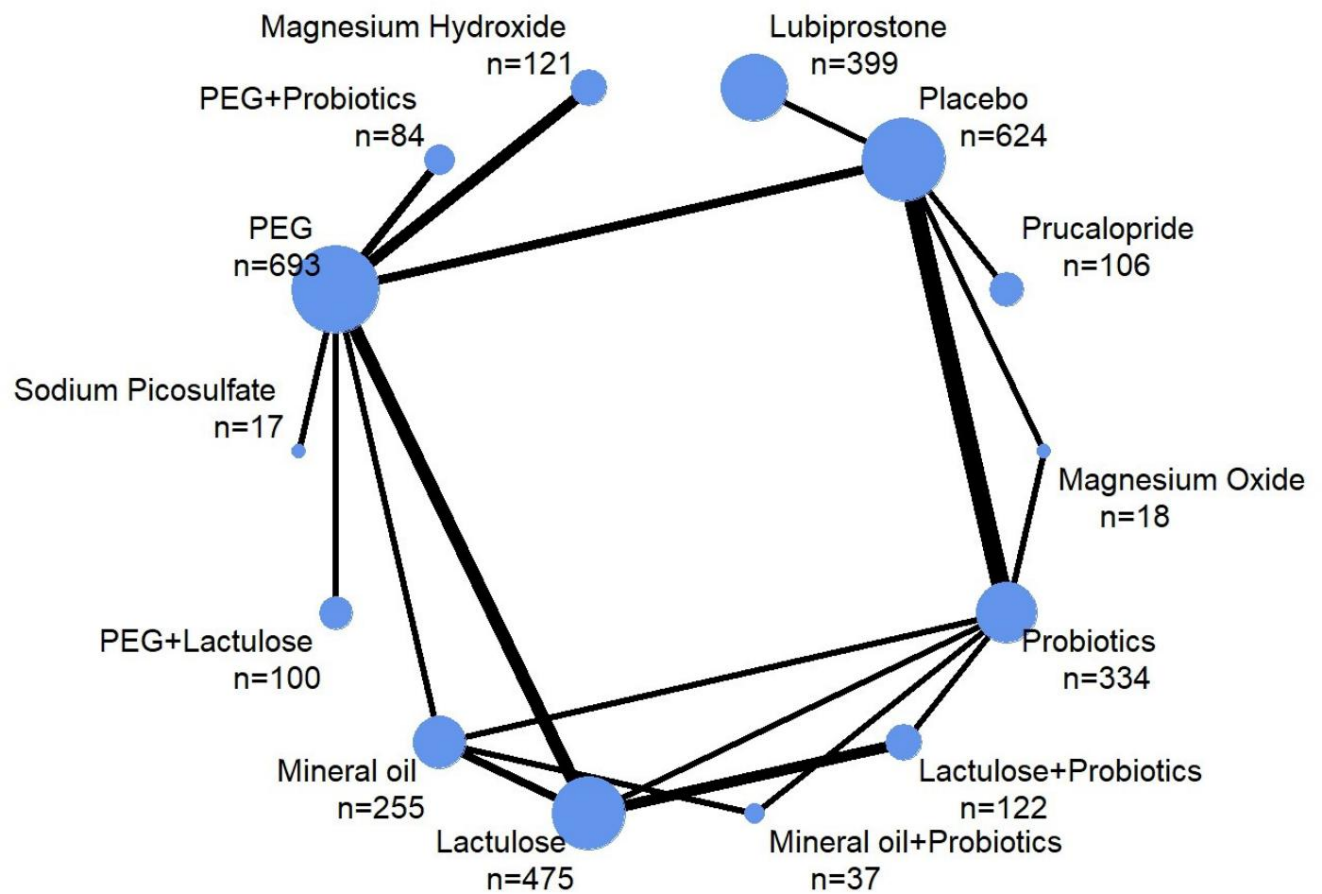
Network Plot Showing Direct Comparisons Between Treatments for the Outcome of Treatment Success. The thickness of lines corresponds to the number of trials comparing the linked treatments. The sizes of the nodes (blue circles) correspond to the number of participants randomized to each treatment. Abbreviation: PEG, polyethylene glycol

There were 29 RCTs of 14 interventions (various combinations of 9 different active treatment components + placebo), which provided data on treatment success to allow pooling of results in network meta-analysis (Figure 4). There were 15 different comparison designs from direct evidence of 35 pairwise comparisons of these interventions. A total of 3385 children with FC were included in the analysis.

### Comparison of probiotics to conventional treatments

**Figure 5.**
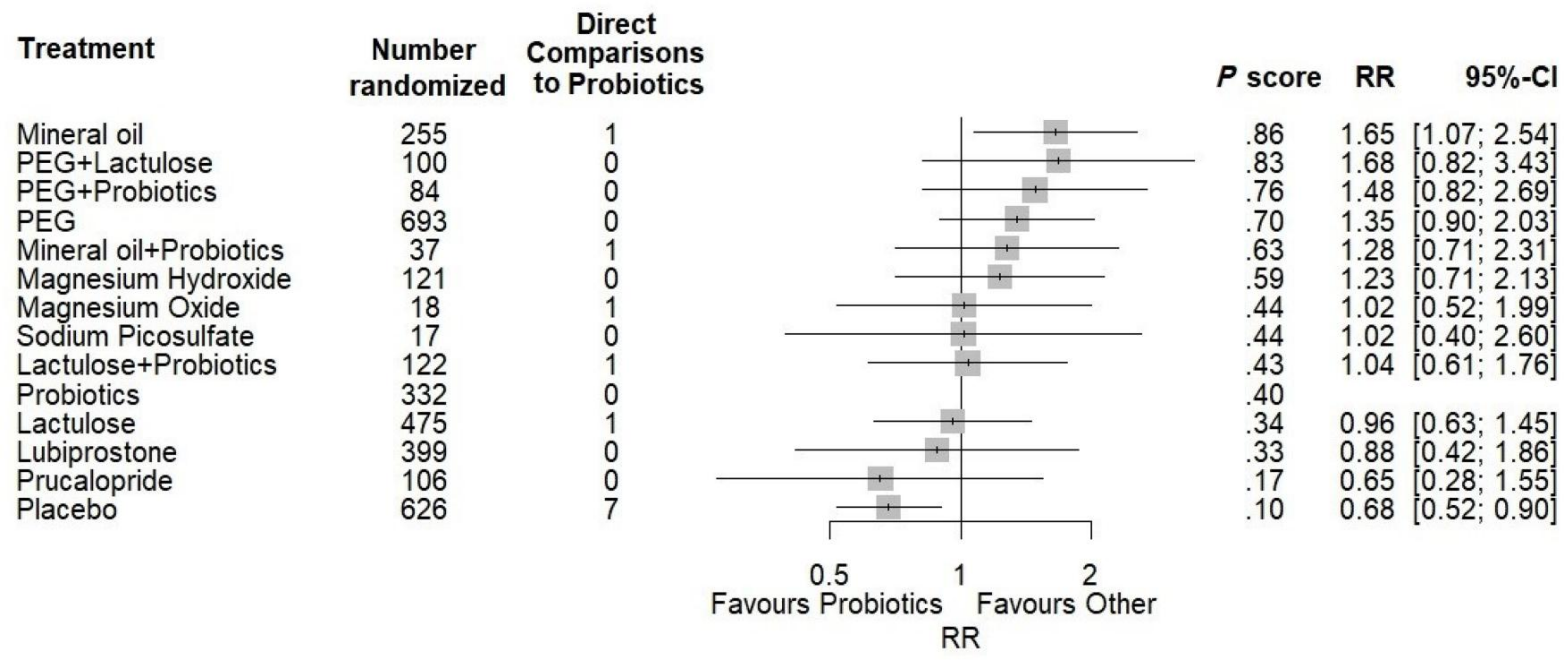
Forest Plot Depicting the Treatment Effects of Conventional Treatments and Placebo as Compared With Probiotics From Random-Effects Network Meta-analysis of Treatment Success. Pooled effect estimates (squares) with 95% CIs are shown for both standard network meta-analyses. Treatments are ordered from highest to lowest *P* scores (probability of being the most effective treatment) from the standard network meta-analysis model. Abbreviations: CI, confidence interval; MD, mean difference; PEG, polyethylene glycol; RR, relative risk

Figure 5 shows the comparisons of probiotics to all other treatments and placebo from the network meta-analysis of treatment success, ordered by probability of being the most effective at increasing treatment success. Probiotics did not statistically significantly increase the rate of treatment success when compared with any conventional treatment for FC and was ranked as 10th most effective out of all 14 treatment arms (including placebo) based on the probability of being the most effective treatment (*P* = .400). Polyethylene glycol and lactulose as a combined treatment increased the RR of treatment success as compared with probiotics (RR: 1.65; 95% CI: 1.07, 2.54) and was ranked as having the highest probability of being the most effective treatment at increasing RR of treatment success (*P* = .863). No other treatments statistically significantly increased the rate of treatment success as compared with probiotics.

### Conventional treatments and probiotics as compared with placebo

The effect estimates for the comparisons between all treatments in the network meta-analysis of treatment success, including the comparisons between conventional treatments, probiotics, and placebo, are shown in the league table (Table 4). When compared with placebo in a standard network meta-analysis, PEG with lactulose as a combined treatment had the greatest effect on treatment success (RR = 2.45; 95% CI: 1.21, 4.97). However, mineral oil had a higher probability of being the most effective treatment (*P* = .863) due to greater precision around the estimate (RR = 2.41; 95% CI: 1.53, 3.81). Polyethylene glycol was also effective at increasing the RR of success as a standalone treatment (RR = 1.97; 95% CI: 1.33, 2.93) and when combined with probiotics (RR = 2.16; 95% CI: 1.20, 3.90). The only other treatments to show a statistically significant effect on treatment success were probiotics (RR = 1.46; 95% CI: 1.11, 1.92) and magnesium hydroxide (RR = 1.80; 95% CI: 1.05, 3.09). Probiotics did not confer any benefit on defecation frequency, indicating they may be more effective at improving other outcomes used in the trials to measure treatment success, such as decreasing fecal incontinence. However, it is worth noting that these effects were reduced when an additive component network meta-analysis was conducted, and effects bordered on nonsignificance (RR = 1.21; 95% CI: 1.02, 1.43). All other treatment effects were not statistically significant, but were large enough to indicate a potential benefit, particularly for mineral oil combined with probiotics (RR = 1.87; 95% CI: 1.00, 3.50), which bordered on statistical significance.

**Table 4.** League Table for the Outcome of Treatment Success (Relative Risk)

Mineral oil			1.18 [0.62; 2.22]^a^	1.09 [0.59; 2.03]					1.17 [0.62; 2.20]^a^	2.12 [1.33; 3.38]			
0.98 [0.49; 1.98]	PEG + lactulose		1.24 [0.69; 2.24]										
1.11 [0.63; 1.98]	1.13 [0.55; 2.35]	PEG + probiotics	1.10 [0.71; 1.70]										
1.22 [0.84; 1.79]	1.24 [0.69; 2.24]	1.10 [0.71; 1.70]	PEG		1.10 [0.76; 1.58]		1.33 [0.57; 3.10]			**1.47 [1.07; 2.02]**			**1.72 [1.04; 2.86]**
1.29 [0.72; 2.30]	1.31 [0.55; 3.10]	1.16 [0.54; 2.49]	1.05 [0.56; 1.98]	Mineral oil + probiotics					1.07 [0.56; 2.02]				
1.34 [0.79; 2.27]	1.36 [0.68; 2.72]	1.20 [0.68; 2.13]	1.10 [0.76; 1.58]	1.04 [0.50; 2.17]	Magnesium hydroxide								
1.62 [0.73; 3.60]	1.65 [0.62; 4.39]	1.46 [0.59; 3.57]	1.33 [0.61; 2.90]	1.26 [0.52; 3.08]	1.21 [0.51; 2.87]	Magnesium oxide			0.93 [0.47; 1.84]				6.50 [0.92; 45.88]
1.62 [0.64; 4.11]	1.65 [0.59; 4.63]	1.46 [0.56; 3.78]	1.33 [0.57; 3.10]	1.26 [0.44; 3.64]	1.21 [0.48; 3.06]	1.00 [0.32; 3.18]	Sodium picosulfate						
1.59 [0.95; 2.67]	1.61 [0.76; 3.43]	1.43 [0.75; 2.71]	1.30 [0.81; 2.09]	1.23 [0.60; 2.54]	1.18 [0.65; 2.16]	0.98 [0.42; 2.30]	0.98 [0.37; 2.58]	Lactulose + probiotics	1.82 [0.85; 3.90]	0.97 [0.64; 1.46]			
**1.65 [1.07; 2.54]**	1.68 [0.82; 3.43]	1.48 [0.82; 2.69]	1.35 [0.90; 2.03]	1.28 [0.71; 2.31]	1.23 [0.71; 2.13]	1.02 [0.52; 1.99]	1.02 [0.40; 2.60]	1.04 [0.61; 1.76]	Probiotics	0.49 [0.23; 1.04]			1.53 [1.14; 2.05]
**1.72 [1.20; 2.46]**	1.75 [0.91; 3.35]	1.54 [0.92; 2.59]	1.41 [1.06; 1.86]	1.34 [0.71; 2.51]	1.28 [0.81; 2.04]	1.06 [0.48; 2.33]	1.06 [0.43; 2.59]	1.08 [0.73; 1.61]	1.04 [0.69; 1.58]	Lactulose			
1.87 [0.81; 4.29]	1.90 [0.70; 5.11]	1.68 [0.67; 4.16]	1.53 [0.69; 3.39]	1.45 [0.57; 3.69]	1.39 [0.58; 3.36]	1.15 [0.42; 3.13]	1.15 [0.36; 3.69]	1.18 [0.49; 2.84]	1.13 [0.54; 2.39]	1.08 [0.48; 2.45]	Lubiprostone		1.29 [0.65; 2.59]
2.52 [0.99; 6.42]^a^	2.56 [0.87; 7.55]	2.26 [0.83; 6.19]	2.06 [0.83; 5.11]	1.96 [0.70; 5.48]	1.88 [0.71; 5.00]	1.55 [0.52; 4.62]	1.55 [0.45; 5.38]	1.59 [0.60; 4.23]	1.53 [0.65; 3.61]	1.47 [0.58; 3.68]	1.35 [0.46; 3.94]	Prucalopride	0.96 [0.42; 2.16]
**2.41 [1.53; 3.81]** ^a^	**2.45 [1.21; 4.97]**	**2.16 [1.20; 3.90]**	**1.97 [1.33; 2.93]**	**1.87 [1.00; 3.50]**	**1.80 [1.05; 3.09]**	1.49 [0.72; 3.06]	1.48 [0.58; 3.79]	1.52 [0.88; 2.62]	1.46 [1.11; 1.92]	1.40 [0.92; 2.14]	1.29 [0.65; 2.59]	0.96 [0.42; 2.16]	Placebo

*Abbreviations:* CINeMA, Confidence in Network Meta-Analysis; PEG, polyethylene glycol.

Treatment effects shown were calculated as relative risks and 95% CIs and based on direct evidence (above treatment names) and mixed evidence from network meta-analyses (below treatment names). Blank cells in the upper right triangle indicate that no direct evidence exists for that comparison. Treatments are ordered from lowest to highest to lowest probability of being the most effective at increasing the risk of treatment success. Statistically significant treatments effects are shown in bold.

a There is low certainty in the evidence of these effect sizes according to assessments from the CINeMA Framework.

### Comparison between conventional therapies to a combination of conventional therapy and probiotics

Comparisons between a combined treatment of conventional treatments and an additional probiotic component to the same standalone conventional treatment from both standard network meta-analysis models for defecation frequency and treatment success are shown in the league tables (Tables 3 and 4). Table 5 shows these effects with the standalone treatment as the reference group for all comparisons.

**Table 5.** Comparison Between Conventional Therapies to a Combination of Conventional Therapy and Probiotics From Network Meta-analysis of Mean Difference in Bowel Movements/Week and Relative Risk of Treatment Success

	PEG + probiotics vs PEG	Lactulose + probiotics vs lactulose	Mineral oil + probiotics vs mineral oil	Magnesium oxide + probiotics vs magnesium oxide
Network meta-analysis of 41 studies of mean difference in BMs/wk				
Studies providing direct evidence	5	5	1	1
MD in BMs/wk	−0.017	0.125	0.483	−0.191
95% CI of MD	−0.938, 0.905	−0.790, 1.040	−1.91, 2.880	−2.021, 1.639
Network meta-analysis of 29 studies of RR of treatment success				
Studies providing direct evidence	2	3	1	
RR of treatment success	1.098	1.081	0.777	
95% CI of RR	0.711, 1.696	0.738, 1.583	0.434, 1.386	

*Abbreviations:* BM, bowel movement; CI, confidence interval; MD, mean difference; NMA, network meta-analysis; PEG, polyethylene glycol; RR, relative risk.

Effects are calculated with the standalone treatment groups as the reference group—ie, an MD >0 or RR >1 indicates that the combined treatment with probiotics performed better than the standalone treatment. Comparisons shown are generated from the same NMA models as previously reported, which differ only by specifying a different treatment as the reference group to obtain effect sizes and CIs for the comparison of interest. Alternatively, results for these comparisons can be obtained from league tables (Tables 3 and 4), where the magnitude of the effects, including 95% CIs, is the same. For effects of MDs in BMs/wk, the sign of the effect and the upper and lower bounds of the confidence limits are opposite to the effect shown in the league table (Table 3) when the reference group is swapped. For effects of RRs of treatment success, the effect sizes and 95% CI limits are equal to the inverse of the effect from the league table (Table 4) when the reference group is swapped.

### Defecation frequency

The most notable difference between the combination treatment and standalone conventional treatment was for the treatment with mineral oil. A combined treatment of mineral oil and probiotics increased the number of BMs per week by 0.48 when compared with mineral oil alone, but this was not statistically significant (95% CI: –1.91, 2.88). There were very small differences between the number of BMs/wk when comparing the standalone treatments of PEG, lactulose, and magnesium oxide with a combination treatment of these with probiotics. The combination treatment of PEG and probiotics performed very similarly to the standalone treatment of PEG (MD: –0.017; 95% CI: –0.938, 0.905). This was also the case for magnesium oxide (MD: –0.191; 95% CI: -2.021, 1.639) and lactulose (MD: 0.125; 95% CI: –0.790, 1.040).

### Treatment success

In contrast to the effect of defecation frequency, the combined treatment of mineral oil and probiotics was less effective at increasing the rate of treatment success than mineral oil alone (RR: 0.78), but this was also not statistically significant (95% CI: 0.434, 1.39). Probiotics did not increase treatment success rates when given as an additional component to PEG (RR: 1.098; 95% CI: 0.711, 1.696) or lactulose (RR: 1.081; 95% CI: 0.738, 1.583). There was no effect estimate for the comparison of treatment success for magnesium oxide with probiotics to magnesium oxide alone because only 1 study used this comparison design and did not report on this outcome. Overall, the treatment effects comparing conventional treatments and the same treatments administered in combination with probiotics are similar for the outcomes of defecation frequency or treatment success. Probiotics do not seem to be efficacious at increasing the treatment effects of conventional treatments in children with FC.

### Adverse events

The number of adverse events within each trial is summarized in Table S7. Overall, adverse events were balanced between groups, suggesting safety.

### Risk of bias

An overview of the RoB assessments for each domain in all included studies is presented in the supplementary material (Figure S3). Only 4 RCTs received an overall RoB rating of “low” for at least 1 outcome of interest.^45^^,^^49^^,^^51^^,^^63^ There were 11 RCTs that received a rating of “some concerns for all relevant outcomes,”^30^^,^^35^^,^^36^^,^^40^^,^^43^^,^^56^^,^^60^^,^^61^^,^^64^^,^^68^^,^^71^ while the remaining were rated as “high risk.” The RCT conducted by Tabbers et al^45^ was rated as “low risk” for the outcome of defecation frequency but “some concerns” for the outcome of treatment success due to differences in missing outcome data, and therefore contributed to the total counts of both. The remaining studies were rated as “high risk.”^28^^,^^29^^,^^31–34^^,^^37–39^^,^^41^^,^^42^^,^^44^^,^^46–48^^,^^50^^,^^52–55^^,^^57–59^^,^^62^^,^^65–67^^,^^69^^,^^70^^,^^72–76^

It was common for studies to not report the method of allocation concealment, which is an important component of domain 1, leading to a “some concerns” rating in this domain. There were only 3 RCTs that were rated as “high risk” for this domain because there was no method of randomization described and baseline differences in defecation frequency suggested that the randomization process may not have been optimal.^62^^,^^72^^,^^74^

When assessing domain 2, many studies received a high RoB because authors tended to exclude participants who did not adhere to their assigned intervention after randomization had occurred.^28^^,^^30^^,^^32^^,^^50^^,^^57^^,^^58^^,^^62^^,^^64^^,^^67^^,^^73^^,^^74^ Furthermore, many studies did not blind either the participants and their caretakers or the study investigators. A nonblinded study design does not immediately warrant a high RoB rating in this domain; however, it contributes to a higher RoB rating in this domain if there were deviations that arose due to the trial context or if there was no information provided about whether these deviations occurred.

Overall, missing data were not common in the trials due to the relatively short follow-up (usually 4 weeks). Sixteen trials were rated “high risk”^28^^,^^29^^,^^33^^,^^37–39^^,^^50^^,^^52–54^^,^^66^^,^^67^^,^^69^^,^^72–74^, 7 trials were rated as having “some concerns,”^35^^,^^45^^,^^46^^,^^48^^,^^56^^,^^60^^,^^64^ and the remaining trials were rated “low risk.”

Stool frequency, and by extension treatment success, was almost exclusively measured through the use of stool diaries in the RCTs, where participants record their symptoms on a daily basis to ensure accuracy. This was considered to be a reliable form of measurement and these studies were rated as “low risk” when participants using this method were blinded.^28–30^^,^^33^^,^^35–37^^,^^40^^,^^41^^,^^43^^,^^45^^,^^49^^,^^51^^,^^56^^,^^61^^,^^63^^,^^64^^,^^67^^,^^68^^,^^71^^,^^76^ However, there were 26 trials that were not blinded or did not provide information on how blinding was achieved and were rated as “high risk” in this domain because knowledge of the treatment group could affect assessment of the outcome because there is a level of judgment involved.^31^^,^^32^^,^^34^^,^^38^^,^^39^^,^^42^^,^^44^^,^^46–48^^,^^50^^,^^52^^,^^54^^,^^55^^,^^57–59^^,^^62^^,^^65^^,^^66^^,^^69^^,^^70^^,^^72–75^

When assessing domain 5, many trials, particularly those that were older and published before prespecifying a protocol was common, did not state whether the trial adhered to a protocol^28^^,^^29^^,^^31–34^^,^^37^^,^^38^^,^^46^^,^^50–52^^,^^79^ or did not provide sufficient information to locate the protocol^44^^,^^58^ required to compare the analysis provided with the analysis plan. Also notable is that some trials had a protocol that was registered retrospectively.^41^^,^^53^^,^^57^^,^^59^ In both of these instances, results could have been selectively reported based on their magnitude or statistical significance.

Five trials had a registered protocol, but had discrepancies between the intended prespecified methods and what was reported in the paper^57^^,^^63^^,^^66^ or the protocol was not specific enough to rule out that selective reporting had taken place.^64^^,^^69^ One of these was assessed as having “some concerns” rather than “high risk” because the discrepancies related to outcomes other than treatment success or defecation frequency.^66^ Only 7 studies^45^^,^^49^^,^^56^^,^^61^^,^^64^^,^^67^^,^^74^ had a preregistered protocol and adhered to the prespecified methods therein, leading to a low RoB rating.

### Confidence and consistency in the networks

In the design-by-treatment chi-square test to detect global inconsistency for the network of defecation frequency, the assumption of coherence was met (χ^2^ = 13.17; *P* = .357). The results of the local test of inconsistency using the SIDDE test are shown in Table S8. Inconsistency was identified between direct and indirect evidence for the comparisons of mineral oil to lactulose (*P* = .002), magnesium oxide to probiotics (*P* = .058), and mineral oil to PEG (*P* = .011). In other words, there was disagreement between the direct evidence observed in the trials comparing these treatments and the indirect evidence generated by the network meta-analysis model. These differences could have been brought about by between-study differences in participant characteristics that affect the outcomes of interest. These characteristics include defecation frequency at baseline, age of the participants, and length of follow-up after treatment. Many of the other direct comparisons in the network were based on few studies, and therefore may have been underpowered to detect inconsistency.

In the design-by-treatment chi-square test to detect global inconsistency for the network of treatment success, the assumption of coherence was met (χ^2^ = 11.05, *P* = .050). The comparison between direct and indirect evidence by comparison group for the network of treatment success is shown in Table S9. Inconsistency was identified between direct and indirect evidence for the comparisons of lactulose to lactulose combined with probiotics (*P* = .016), lactulose to probiotics (*P* = .018), and probiotics to lactulose combined with probiotics (*P* = .047).

The ratings for each domain of the CINeMA framework to assess confidence in the treatment effects are shown in Figures S4 and S5. The overall confidence in the effects ranged from low to very low, as shown in the league tables of defecation frequency (Table 3) and treatment success (Table 4). Therefore, future research on the topic is likely to change the overall pooled effect of the treatment. Visual inspection of comparison-adjusted funnel plots did not suggest publication bias (Figures S6 and S7). However, it was suspected that there was RoB due to missing evidence in the comparison of PEG to PEG combined with probiotics.

The low certainty in the treatment effects was largely driven by the high levels of within-study bias of the RCTs and exacerbated by inconsistency between direct and indirect evidence.

## DISCUSSION

This network meta-analysis is novel in comparing the effectiveness of oral maintenance therapies on defecation frequency and treatment success in children with FC. There were 3470 children from 41 RCTs analyzed for the outcome of defecation frequency and 3385 children from 29 RCTs analyzed for the outcome of treatment success. This analysis found that PEG, a combination of PEG and probiotics, mineral oil, a combination of mineral oil with probiotics, and magnesium hydroxide were the only treatments to show a statistically significant increase in defecation frequency when compared with placebo. The same treatments, in addition to a combined treatment of PEG and lactulose, were more effective than placebo at improving treatment success.

Probiotics have recently emerged as a treatment of interest for FC in both adults and children. In the present review, there were 23 RCTs^29^^,^^33^^,^^36^^,^^40^^,^^41^^,^^43^^,^^45^^,^^48^^,^^50^^,^^53^^,^^55–58^^,^^61–63^^,^^67^^,^^68^^,^^72–74^^,^^76^ that reported on either defecation frequency or treatment success and compared probiotics with various other treatments, 13 of which were a combined mixture of different strains.^36^^,^^41^^,^^43^^,^^45^^,^^50^^,^^53^^,^^55^^,^^57^^,^^58^^,^^62^^,^^68^^,^^72^^,^^73^ It was common for RCTs to compare probiotics in combination with other treatments, making it difficult for previous systematic reviews to distinguish the effect of the probiotic treatment separately from the treatment it was combined with in pairwise meta-analyses.^11^ Results from the present network meta-analysis showed that probiotics as an isolated treatment were not effective in increasing defecation frequency when compared with any conventional treatment, including PEG, mineral oil, lactulose, magnesium hydroxide, or magnesium oxide. Notably, when well-established treatments such as PEG, mineral oil, lactulose, or magnesium oxide were combined with probiotics, treatment effects were similar to those from the standalone treatment, indicating that the combined effect is mostly owing to the lactulose, mineral oil, magnesium oxide, or PEG components rather than an equal contribution of probiotics and these treatments on the outcome.

### Comparison to previous systematic reviews

The results from this systematic review and network meta-analysis are largely in agreement with previous systematic reviews and meta-analyses. The results are consistent with a recently published Cochrane review by Wallace et al,^10^ which found that there is insufficient evidence to determine whether probiotics are more efficacious than placebo in improving treatment success rates or increasing defecation frequency in children with FC. A Cochrane review conducted by Lee-Robichaud et al^81^ in 2010 found that PEG was more efficacious than lactulose in improving stool frequency in adults and children. An additional Cochrane review conducted in 2016 found that PEG may be more effective than lactulose and magnesium hydroxide.^82^ These analyses were based on few studies and reliance on pairwise meta-analysis and, therefore, conclusions were suggested much more cautiously. The present study builds on these findings by conducting a more robust analysis by utilizing both direct and indirect evidence in network meta-analysis and provides a point of comparison for all oral maintenance therapies for FC in children.

Functional constipation is a common issue affecting children, and early treatment is required to minimize further hardening of stools, withholding, and subsequent soiling. Current guidelines recommend PEG as the preferred treatment option and lactulose as the second choice when PEG is unavailable.^5^^,^^83^ Polyethylene glycol and lactulose are both osmotic laxatives that create an osmotic gradient to increase luminal fluid, lower the pH, and increase peristalsis.^5^^,^^84^ However, they interact with the gut slightly differently. Lactulose is a derivative of lactose and needs to be fermented into low-molecular-weight acids by bacteria present in the lumen.^3^ On the other hand, PEG is a linear polymer and does not need to be metabolized before it can begin to draw water into the lumen through osmosis.^3^

Results from this systematic review suggested that mineral oil was more beneficial in improving constipation symptoms than lactulose and was ranked as having the highest probability of being the most effective treatment for treatment success. It is important to consider, however, that all effect sizes that compared mineral oil with another treatment were rated as “high risk” of bias according to the Cochrane RoB 2 tool. Furthermore, there are some limitations of using mineral oil as a treatment. Mineral oil is an emollient and increases the frequency of BMs by lubricating the stool and decreasing friction. Due to the fact that it is not absorbed, oil leakage is a potential side effect.^3^^,^^5^ It cannot be given to children less than 3 years of age or to children with gastroesophageal reflux or neurodevelopmental disorders due to risk of aspiration.^3^ Therefore, PEG should be prescribed in these cases.

An important clinical consideration of the present review is how probiotics may affect other systems of the body in addition to the gastrointestinal system. It is known that the gut microbiota affects the function of other organs in the body, with increasing investigation into the gut–lung axis.^85^ Both the gut and lungs share exposure to microbes through the oral route.^86^ Therefore, taking probiotics orally may affect the presence of these bacteria in the respiratory system.^87^ Currently, it is understood that probiotics influence the relationship between commensal microbes and the mucosal immune framework, which may lead to differences in responses to viral pathogens and other inflammatory respiratory conditions.^85–87^ The probiotics examined in the present review usually belonged to the genus *Lactobacillus*. Many strains from this genus have been found to be related to various functions and health outcomes related to the respiratory system.^88^  *Lactobacillus rhamnosus* GG was examined in the present review as a singular strain by 1 study^29^ and as a probiotic mixture with other strains in multiple studies. This strain has been shown to reduce the risk of respiratory infection from influenza virus and reduce the incidence and number of days with respiratory tract infection from rhinovirus.^86^ A commonly evaluated strain of probiotic in the RCTs included in the present systematic review was *Lactobacillus reuteri* DSM 17938. This strain has been shown to excrete antimicrobial molecules, promote the function of regulatory T cells,^89^^,^^90^ and also has anti-inflammatory properties.^91^ Therefore, this strain may also prevent infection of pathogens in the lungs as well as reduce the symptoms of inflammatory conditions such as asthma.^89^ Overall, the probiotics examined by the studies in the present systematic review seem to provide beneficial effects for functioning of the respiratory system. A systematic review and meta-analysis of RCTs found that children randomized to probiotics had a reduced risk of respiratory tract infection and had a fewer number of days with respiratory tract infection.^92^ In the present review, there were no significant concerns regarding adverse events in RCTs examining probiotics, including those involving any respiratory issues.

This systematic review is the first to analyze all studies comparing oral maintenance therapies in a network meta-analysis and rank treatments based on their effectiveness at improving symptoms of FC. Indirect evidence from network meta-analysis models used to analyze defecation frequency and treatment success has enabled comparisons between treatments that were not compared directly in the original trials, and that are unable to be compared in a pairwise meta-analysis.

### Limitations

The findings of this research have limitations that must be considered when interpreting the results from this network meta-analysis. A main limitation of this review is that there were a range of different probiotic strains examined by the RCTs included, making it difficult to analyze the effect of different strains on the outcomes of interest. In one-third of all RCTs examining probiotics, the treatment group received a combination of different probiotic strains. In the present network meta-analysis, different strains of probiotics were grouped together and analyzed as a singular treatment. Therefore, this review did not investigate the potential for strain-specific effects to influence BMs per week and treatment success rates in children with FC.

Within-study RoB was considered to be either “some concerns” or “high risk” for all but 5 RCTs analyzed. This was a major contributing factor to the confidence in the majority of estimates from network meta-analyses being assessed as low and very low. This means that the true effect of the treatments analyzed could be markedly different from the effect estimates generated from the network meta-analysis models.

Another limitation is that there was some incoherence detected in the networks. In other words, there was a difference between the observed effect sizes and the predicted effect sizes calculated from indirect evidence. The studies that compared these may have differed from other studies in the network with regard to important effect modifiers, such as duration of constipation and BMs at baseline.

Two treatments (lubiprostone and prucalopride) were only examined once across the RCTs in the networks. These treatments were similar to placebo in their effectiveness; however, it is possible that a true effect exists but was not able to be detected due to lack of power.

Secondary outcomes, such as fecal incontinence, abdominal pain, and bloating, were not considered in this review as it was anticipated that these outcomes would not be reported consistently enough to enable comparison in a network meta-analysis. It is possible that treatments ranked with a high probability of being the most effective at increasing BMs/wk and treatment success rates, such as mineral oil and PEG, may not be as effective in improving these other symptoms, which are common in FC.

## CONCLUSION 

It is clear that more high-quality RCTs are needed to improve the quality of evidence in this research area, increase the certainty in results from meta-analysis, and enable better clinical recommendations that are more reflective of the effectiveness of the treatments analyzed. Particular domains that had a high RoB in the current set of RCTs were blinding of participants and study personnel, randomization sequence concealment, and adherence to a published protocol with a prespecified analysis plan.

There are various areas on this topic that warrant further investigation. Thus far, lubiprostone and prucalopride have only been evaluated in 1 RCT in children. Although these trials did not show any statistically significant benefit over their comparators in the present systematic review, these medications have shown promise in adults with FC^93–95^ and more RCTs in children are needed to determine their effectiveness in this population. There were no RCTs evaluating other novel pharmacological treatments, such as linaclotide, naronapride, or plecanatide, which have also shown promising results in adults.^96^

Future studies are also needed to explore the differences between the effect of different strains of probiotics. In this analysis, different strains and mixtures of probiotics were evaluated together with *L reuteri* being the most commonly evaluated strain. However, other less studied strains, such as *Bifidobacterium lactis*, *Bifidobacterium brevis*, and *L rhamnosus* could impact the gastrointestinal tract differently. Future RCTs should continue to evaluate these different strains of probiotics as an additional component to conventional treatments and compare their effectiveness with the conventional treatment without any probiotic component. It is important that these studies be free from RoB in the randomization, concealment of allocation, and blinding components of the trial, so that any effect of an additional probiotic component can be attributed to the probiotic rather than to bias in the RCT.

In conclusion, results from this network meta-analysis support the use of PEG and mineral oil for maintenance therapy for children with FC. Probiotics were not found to be beneficial in improving defecation frequency or successfully treating children with FC. Caution should be exercised in interpreting the results due to the low certainty of the evidence. The evidence from the present review suggests that clinicians should not prescribe probiotics as a standalone treatment to children with FC. Polyethylene glycol remains the preferred treatment for maintenance therapy in children with FC.

## Supplementary Material

nuae119_Supplementary_Data

## Data Availability

Data described in the manuscript and all code used to run analyses are presented in the online supporting material.
