# Supplementary material for: Efficacy of Probiotics Compared With Pharmacological Treatments for Maintenance Therapy for Functional Constipation in Children: A Systematic Review and Network Meta-analysis
Source: Nutr Rev. 2024 Sep 30;83(6):1006–34. doi: 10.1093/nutrit/nuae119 (PMC12066947; doi:10.1093/nutrit/nuae119)
Supplement: nuae119_Supplementary_Data [file nuae119_supplementary_data.docx]

**The efficacy of probiotics compared to pharmacological treatments for maintenance therapy for functional constipation in children: A systematic review and network meta-analysis.**

**Rebecca G Harris, Elizabeth P Neale, Marijka Batterham**

**Supplementary Materials**

[**Supplementary Table S1** PRISMA-NMA checklist. 3](#_Toc173415445)

[**Supplementary Table S2** Details of systematic search strategy. 6](#_Toc173415446)

[**Supplementary Table S3** Dataset used in the NMA of defecation frequency and treatment success. 10](#_Toc173415447)

[**Supplementary Table S4** Code used in the NMA of defecation frequency and treatment success in R software. This code will run after saving the dataset from Supplementary Table S3 in an excel file as ‘Dataset’ in the working directory as an excel file. 14](#_Toc173415448)

[**Supplementary Table S5** Trial protocols identified and their publication status. 17](#_Toc173415449)

[**Supplementary Table S6** List of the 77 reports excluded during full text screening (with reasons). 19](#_Toc173415450)

[**Supplementary Table S7** Overview of adverse events as reported in the RCTs in the present review. 22](#_Toc173415451)

[**Supplementary Table S8** Comparison between direct and indirect evidence by comparison for the NMA of defecation frequency. 32](#_Toc173415452)

[**Supplementary Table S9** Comparison between direct and indirect evidence by comparison for the NMA of treatment success. 33](#_Toc173415453)

[**Supplementary Figure S1** Boxplots comparing A) BMs/wk at baseline, B) duration of follow-up, C) sample size, D) publication year, E) mean age of participants, F) duration of FC and G) Percentage female participants in the RCTs contributing to the analysis of defecation frequency by treatment group. 34](#_Toc173415454)

[**Supplementary Figure S2** Boxplots comparing A) BMs/wk at baseline, B) duration of follow-up, C) sample size, D) publication year, E) mean age of participants, F) duration of FC and G) Percentage female participants in the RCTs contributing to the analysis of treatment success by treatment group. 38](#_Toc173415455)

[**Supplementary Figure S3** Overview of ROB assessments according to the Cochrane ROB2 tool for all studies included in the present systematic review. 42](#_Toc173415456)

[**Supplementary Figure S4** Confidence in the estimates of the comparisons in the network of defecation frequency using CINeMA. 43](#_Toc173415457)

[**Supplementary Figure S5** Confidence in the estimates of the comparisons in the network of treatment success using CINeMA. 45](#_Toc173415458)

[**Supplementary Figure S6** Funnel plot depicting the effect size by standard error for each comparison contributing to the analysis of defecation frequency. 47](#_Toc173415459)

[**Supplementary Figure S7** Funnel plot depicting the effect size by standard error for each comparison contributing to the analysis of treatment success. 48](#_Toc173415460)

[**References** 49](#_Toc173415461)

## **Supplementary Table S1** PRISMA-NMA checklist.

| **Section/Topic** | **Item #** | **Checklist Item** | **Reported on Page #** |
| --- | --- | --- | --- |
| **TITLE** |  |  |  |
| Title | 1 | Identify the report as a systematic review *incorporating a network meta-analysis (or related form of meta-analysis).* | ***1*** |
|  |  |  |  |
| **ABSTRACT** |  |  |  |
| Structured summary | 2 | Provide a structured summary including, as applicable:  **Background:** main objectives  **Methods:** data sources; study eligibility criteria, participants, and interventions; study appraisal; and *synthesis methods, such as network meta-analysis.*  **Results:** number of studies and participants identified; summary estimates with corresponding confidence/credible intervals; *treatment rankings may also be discussed. Authors may choose to summarize pairwise comparisons against a chosen treatment included in their analyses for brevity.*  **Discussion/Conclusions:** limitations; conclusions and implications of findings.  **Other:** primary source of funding; systematic review registration number with registry name. | 2 |
|  |  |  |  |
| **INTRODUCTION** |  |  |  |
| Rationale | 3 | Describe the rationale for the review in the context of what is already known*, including mention of why a network meta-analysis has been conducted.* | ***4*** |
| Objectives | 4 | Provide an explicit statement of questions being addressed, with reference to participants, interventions, comparisons, outcomes, and study design (PICOS). | 4 |
|  |  |  |  |
| **METHODS** |  |  |  |
| Protocol and registration | 5 | Indicate whether a review protocol exists and if and where it can be accessed (e.g., Web address); and, if available, provide registration information, including registration number. | 5 |
| Eligibility criteria | 6 | Specify study characteristics (e.g., PICOS, length of follow-up) and report characteristics (e.g., years considered, language, publication status) used as criteria for eligibility, giving rationale. *Clearly describe eligible treatments included in the treatment network, and note whether any have been clustered or merged into the same node (with justification).* | ***5-6*** |
| Information sources | 7 | Describe all information sources (e.g., databases with dates of coverage, contact with study authors to identify additional studies) in the search and date last searched. | 6 |
| Search | 8 | Present full electronic search strategy for at least one database, including any limits used, such that it could be repeated. | Table S1 |
| Study selection | 9 | State the process for selecting studies (i.e., screening, eligibility, included in systematic review, and, if applicable, included in the meta-analysis). | Table 1  Page 6 |
| Data collection process | 10 | Describe method of data extraction from reports (e.g., piloted forms, independently, in duplicate) and any processes for obtaining and confirming data from investigators. | 7 |
| Data items | 11 | List and define all variables for which data were sought (e.g., PICOS, funding sources) and any assumptions and simplifications made. | 7 |
| **Geometry of the network** | **S1** | Describe methods used to explore the geometry of the treatment network under study and potential biases related to it. This should include how the evidence base has been graphically summarized for presentation, and what characteristics were compiled and used to describe the evidence base to readers. | ***8*** |
| Risk of bias within individual studies | 12 | Describe methods used for assessing risk of bias of individual studies (including specification of whether this was done at the study or outcome level), and how this information is to be used in any data synthesis. | 9 |
| Summary measures | 13 | State the principal summary measures (e.g., risk ratio, difference in means). *Also describe the use of additional summary measures assessed, such as treatment rankings and surface under the cumulative ranking curve (SUCRA) values, as well as modified approaches used to present summary findings from meta-analyses.* | 8 |
| Planned methods of analysis | 14 | Describe the methods of handling data and combining results of studies for each network meta-analysis. This should include, but not be limited to:   - *Handling of multi-arm trials;* - *Selection of variance structure;* - *Selection of prior distributions in Bayesian analyses; and* - *Assessment of model fit.* | 8 |
| **Assessment of Inconsistency** | **S2** | Describe the statistical methods used to evaluate the agreement of direct and indirect evidence in the treatment network(s) studied. Describe efforts taken to address its presence when found. | 9 |
| Risk of bias across studies | 15 | Specify any assessment of risk of bias that may affect the cumulative evidence (e.g., publication bias, selective reporting within studies). | **10** |
| Additional analyses | 16 | Describe methods of additional analyses if done, indicating which were pre-specified. This may include, but not be limited to, the following:   - Sensitivity or subgroup analyses; - Meta-regression analyses; - *Alternative formulations of the treatment network; and* - *Use of alternative prior distributions for Bayesian analyses (if applicable).* | ***N/A*** |
|  |  |  |  |
| **RESULTS†** |  |  |  |
| Study selection | 17 | Give numbers of studies screened, assessed for eligibility, and included in the review, with reasons for exclusions at each stage, ideally with a flow diagram. | 11  Figure 1 |
| **Presentation of network structure** | **S3** | Provide a network graph of the included studies to enable visualization of the geometry of the treatment network. | ***Figure 2*** |
| **Summary of network geometry** | **S4** | Provide a brief overview of characteristics of the treatment network. This may include commentary on the abundance of trials and randomized patients for the different interventions and pairwise comparisons in the network, gaps of evidence in the treatment network, and potential biases reflected by the network structure. | ***11-12*** |
| Study characteristics | 18 | For each study, present characteristics for which data were extracted (e.g., study size, PICOS, follow-up period) and provide the citations. | Table 2 |
| Risk of bias within studies | 19 | Present data on risk of bias of each study and, if available, any outcome level assessment. | Figure S3 |
| Results of individual studies | 20 | For all outcomes considered (benefits or harms), present, for each study: 1) simple summary data for each intervention group, and 2) effect estimates and confidence intervals. *Modified approaches may be needed to deal with information from larger networks.* |  |
| Synthesis of results | 21 | Present results of each meta-analysis done, including confidence/credible intervals. *In larger networks, authors may focus on comparisons versus a particular comparator (e.g. placebo or standard care), with full findings presented in an appendix. League tables and forest plots may be considered to summarize pairwise comparisons.* If additional summary measures were explored (such as treatment rankings), these should also be presented. | ***12-13***  ***Figure 3***  ***Figure 5***  ***Table 3***  ***Table 4***  ***Table 5*** |
| **Exploration for inconsistency** | **S5** | Describe results from investigations of inconsistency. This may include such information as measures of model fit to compare consistency and inconsistency models, *P* values from statistical tests, or summary of inconsistency estimates from different parts of the treatment network. | ***15-16*** |
| Risk of bias across studies | 22 | Present results of any assessment of risk of bias across studies for the evidence base being studied. | Table S6  Table S7 |
| Results of additional analyses | 23 | Give results of additional analyses, if done (e.g., sensitivity or subgroup analyses, meta-regression analyses*, alternative network geometries studied, alternative choice of prior distributions for Bayesian analyses,* and so forth). | ***12-13*** |
|  |  |  |  |
| **DISCUSSION** |  |  |  |
| Summary of evidence | 24 | Summarize the main findings, including the strength of evidence for each main outcome; consider their relevance to key groups (e.g., healthcare providers, users, and policy-makers). | 17 |
| Limitations | 25 | Discuss limitations at study and outcome level (e.g., risk of bias), and at review level (e.g., incomplete retrieval of identified research, reporting bias). *Comment on the validity of the assumptions, such as transitivity and consistency. Comment on any concerns regarding network geometry (e.g., avoidance of certain comparisons).* | 18, 19 |
| Conclusions | 26 | Provide a general interpretation of the results in the context of other evidence, and implications for future research. | 20 |
|  |  |  |  |
| **FUNDING** |  |  |  |
| Funding | 27 | Describe sources of funding for the systematic review and other support (e.g., supply of data); role of funders for the systematic review. This should also include information regarding whether funding has been received from manufacturers of treatments in the network and/or whether some of the authors are content experts with professional conflicts of interest that could affect use of treatments in the network. | ***1*** |

## **Supplementary Table S2** Details of systematic search strategy.

| Database [#hits] | Search syntax |
| --- | --- |
| **SCOPUS**  [1970 hits]^1^ | (TITLE-ABS-KEY(child* OR adolescen* OR youth OR pediatric OR toddler OR neonat* OR infant* or girl or boy)) AND (TITLE-ABS-KEY(constipat* or costiveness or costive or obstipation or "fecal impaction" or "delayed bowel movement")) AND (TITLE-ABS-KEY(probiotic or synbiotic or lactobacillus or LGG or Lcr35 or Bifidobacterium or “B. Lactis” or GCL2505 or saccharomyces or longum or acidophilus or thermophilus or thermophylus or “Bacillus clausii” or PEG or "polyethylene glycol" or forlax or Miralax or Transipeg or Movicol or Forlax or Idrolax or GoLytely or PMF-100 or Golitely or Nulitely or Fortrans or TriLyte or Colyte or moviprep or sorbitol or glucitol or mannitol or lactulose or lactitol or lactulose or lactitol or generlac or cephulac or cholac or constilac or enulose or cilac or heptalac or actilax or duphalac or Kristalose or "magnesium hydroxide" or "milk of magnesia" or "magnesium citrate" or "magnesium sulfate" or "magnesium salts" or "mg salts" or "magnesium oxide" or MgOH2 or MgOH or MgO or MgSO4 or citroma or diphenylmethane or "sodium picosulfate" or Bisacodyl or senokot or Dulcolax or anthraquinones or senna or sennosides or docusate or Colace or "liquid paraffin" or "paraffin liquid" or "mineral oil" or lubiprostone or Amitiza or linaclotide or Linzess or Constella or prucalopride or Resolor or Motegrity or plecanatide or Trulance or naronapride or "ATI-7505")) |
| **COCHRANE Library**  [577 hits]^1^ | \| ID \| Search \| Hits \| \| --- \| --- \| --- \| \| #1 \| child*:ti,ab,kw \| 174112 \| \| #2 \| MeSH descriptor: [Child] explode all trees \| 61335 \| \| #3 \| adolescen*:ti,ab,kw \| 147270 \| \| #4 \| p*ediatric:ti,ab,kw \| 35642 \| \| #5 \| youth:ti,ab,kw \| 7940 \| \| #6 \| toddler:ti,ab,kw \| 971 \| \| #7 \| neonate*:ti,ab,kw \| 9247 \| \| #8 \| infant*:ti,ab,kw \| 66504 \| \| #9 \| MeSH descriptor: [Infant] explode all trees \| 34864 \| \| #10 \| girls:ti,ab,kw \| 7817 \| \| #11 \| boys:ti,ab,kw \| 6954 \| \| #12 \| #1 or #2 or #3 or #4 or #5 or #6 or #7 or #8 or #9 or #10 or #11 \| 310708 \| \| #13 \| constipat*:ti,ab,kw \| 13841 \| \| #14 \| MeSH descriptor: [Constipation] explode all trees \| 1904 \| \| #15 \| costiveness:ti,ab,kw \| 1 \| \| #16 \| obstipat*:ti,ab,kw \| 62 \| \| #17 \| faecal impcation:ti,ab,kw \| 0 \| \| #18 \| delayed bowel movement:ti,ab,kw \| 0 \| \| #19 \| #13 or #14 or #15 or #16 or #17 or #18 \| 13875 \| \| #20 \| probiotics:ti,ab,kw \| 6122 \| \| #21 \| MeSH descriptor: [Probiotics] explode all trees \| 2542 \| \| #22 \| synbiotic:ti,ab,kw \| 686 \| \| #23 \| MeSH descriptor: [Synbiotics] explode all trees \| 172 \| \| #24 \| lactobacillus:ti,ab,kw \| 5555 \| \| #25 \| reuteri:ti,ab,kw \| 696 \| \| #26 \| Lactis:ti,ab,kw \| 884 \| \| #27 \| bifidobacterium:ti,ab,kw \| 3039 \| \| #28 \| GCL2505:ti,ab,kw \| 7 \| \| #29 \| saccharomyces:ti,ab,kw \| 577 \| \| #30 \| longum:ti,ab,kw \| 751 \| \| #31 \| acidophilus:ti,ab,kw \| 1172 \| \| #32 \| thermophylus:ti,ab,kw \| 1 \| \| #33 \| thermophilus:ti,ab,kw \| 344 \| \| #34 \| bacillus clausii:ti,ab,kw \| 53 \| \| #35 \| laxative:ti,ab,kw \| 1122 \| \| #36 \| MeSH descriptor: [Laxatives] explode all trees \| 200 \| \| #37 \| PEG:ti,ab,kw \| 5456 \| \| #38 \| MeSH descriptor: [Polyethylene Glycols] explode all trees \| 3125 \| \| #39 \| polyethylene glycol:ti,ab,kw \| 2280 \| \| #40 \| macrogol:ti,ab,kw \| 591 \| \| #41 \| moviprep:ti,ab,kw \| 157 \| \| #42 \| transipeg:ti,ab,kw \| 10 \| \| #43 \| sorbitol:ti,ab,kw \| 671 \| \| #44 \| glucitol:ti,ab,kw \| 24 \| \| #45 \| lactulose:ti,ab,kw \| 1574 \| \| #46 \| MeSH descriptor: [Lactulose] explode all trees \| 465 \| \| #47 \| lactitol:ti,ab,kw \| 144 \| \| #48 \| “magnesium hydroxide”:ti,ab,kw \| 430 \| \| #49 \| “milk of magnesia”:ti,ab,kw \| 28 \| \| #50 \| “magnesium citrate”:ti,ab,kw \| 345 \| \| #51 \| “magnesium sulfate”:ti,ab,kw \| 2952 \| \| #52 \| “magnesium salts”:ti,ab,kw \| 38 \| \| #53 \| “mg salts”:ti,ab,kw \| 3 \| \| #54 \| “magnesium oxide”:ti,ab,kw \| 358 \| \| #55 \| MgOH2:ti,ab,kw \| 0 \| \| #56 \| MgOH:ti,ab,kw \| 18 \| \| #57 \| MgO:ti,ab,kw \| 50 \| \| #58 \| MgSO4:ti,ab,kw \| 501 \| \| #59 \| “sodium picosulfate”:ti,ab,kw \| 187 \| \| #60 \| bisacodyl:ti,ab,kw \| 435 \| \| #61 \| diphenylmethane:ti,ab,kw \| 2 \| \| #62 \| anthraquinone:ti,ab,kw \| 30 \| \| #63 \| senna:ti,ab,kw \| 239 \| \| #64 \| sennosides:ti,ab,kw \| 62 \| \| #65 \| docusate:ti,ab,kw \| 72 \| \| #66 \| “liquid paraffin”:ti,ab,kw \| 144 \| \| #67 \| “mineral oil”:ti,ab,kw \| 207 \| \| #68 \| lubiprostone:ti,ab,kw \| 155 \| \| #69 \| amitiza:ti,ab,kw \| 12 \| \| #70 \| linaclotide:ti,ab,kw \| 182 \| \| #71 \| linzess:ti,ab,kw \| 7 \| \| #72 \| constella:ti,ab,kw \| 5 \| \| #73 \| prucalopride:ti,ab,kw \| 213 \| \| #74 \| resolor:ti,ab,kw \| 20 \| \| #75 \| motegrity:ti,ab,kw \| 0 \| \| #76 \| plecanatide:ti,ab,kw \| 50 \| \| #77 \| trulance:ti,ab,kw \| 0 \| \| #78 \| naronapride:ti,ab,kw \| 0 \| \| #79 \| ATI-7505:ti,ab,kw \| 6 \| \| #80 \| #20 or #21 or #22 or #23 or #24 or #25 or #26 or #27 or #28 or #29 or #30 or #31 or #32 or #33 or #34 or #35 or #36 or #37 or #38 or #39 or #40 or #41 or #42 or #43 or #44 or #45 or #46 or #47 or #48 or #49 or #50 or #51 or #52 or #53 or #54 or #55 or #56 or #57 or #58 or #59 or #60 or #61 or #62 or #63 or #64 or #65 or #66 or #67 or #68 or #69 or #70 or #71 or #72 or #73 or #74 or #75 or #76 or #77 or #78 or #79 \| 26534 \| \| #81 \| #12 and #19 and #80 \| 593 \| |
| **EMBASE**  [711 hits]^1^ | ('child'/exp OR 'adolescent'/exp OR 'infant'/exp OR child*:ti,ab,kw OR adolescen*:ti,ab,kw OR youth:ti,ab,kw OR pediatric:ti,ab,kw OR toddler:ti,ab,kw OR neonat*:ti,ab,kw OR infant*:ti,ab,kw or girl:ti,ab,kw or boy:ti,ab,kw) AND  ('constipation'/exp OR constipat*:ti,ab,kw OR 'costiveness':ti,ab,kw OR obstipat*:ti,ab,kw OR 'faecal impaction':ti,ab,kw OR 'delayed bowel movement':ti,ab,kw) AND  ('probiotic agent'/exp OR 'synbiotic agent'/exp OR probiotic:ti,ab,kw OR synbiotic:ti,ab,kw OR lactobacillus:ti,ab,kw OR lgg:ti,ab,kw OR lcr35:ti,ab,kw OR bifidobacterium:ti,ab,kw OR lactis:ti,ab,kw OR gcl2505:ti,ab,kw OR saccharomyces:ti,ab,kw OR longum:ti,ab,kw OR acidophilus:ti,ab,kw OR thermophilus:ti,ab,kw OR thermophylus:ti,ab,kw OR 'bacillus clausii':ti,ab,kw OR 'laxative'/exp OR laxative:ti,ab,kw OR peg:ti,ab,kw OR 'polyethylene glycol':ti,ab,kw OR 'macrogol'/exp or macrogol:ti,ab,kw OR miralax:ti,ab,kw OR transipeg:ti,ab,kw OR movicol:ti,ab,kw OR forlax:ti,ab,kw OR idrolax:ti,ab,kw OR golytely:ti,ab,kw OR 'pmf 100':ti,ab,kw OR golitely:ti,ab,kw OR nulitely:ti,ab,kw OR fortrans:ti,ab,kw OR trilyte:ti,ab,kw OR colyte:ti,ab,kw OR moviprep:ti,ab,kw or sorbitol:ti,ab,kw OR glucitol:ti,ab,kw OR mannitol:ti,ab,kw OR lactulose:ti,ab,kw OR lactitol:ti,ab,kw OR generlac:ti,ab,kw OR cephulac:ti,ab,kw OR cholac:ti,ab,kw OR constilac:ti,ab,kw OR enulose:ti,ab,kw OR cilac:ti,ab,kw OR heptalac:ti,ab,kw OR actilax:ti,ab,kw OR duphalac:ti,ab,kw OR kristalose:ti,ab,kw OR 'magnesium hydroxide':ti,ab,kw OR 'milk of magnesia':ti,ab,kw OR 'magnesium citrate':ti,ab,kw OR 'magnesium sulfate':ti,ab,kw OR 'magnesium salts':ti,ab,kw OR 'mg salts':ti,ab,kw OR 'magnesium oxide':ti,ab,kw OR mgoh2:ti,ab,kw OR mgoh:ti,ab,kw OR mgo:ti,ab,kw OR mgso4:ti,ab,kw OR citroma:ti,ab,kw OR diphenylmethane:ti,ab,kw OR 'sodium picosulfate':ti,ab,kw OR bisacodyl:ti,ab,kw OR senokot:ti,ab,kw OR dulcolax:ti,ab,kw OR anthraquinones:ti,ab,kw OR senna:ti,ab,kw OR sennosides:ti,ab,kw OR docusate:ti,ab,kw OR colace:ti,ab,kw OR 'liquid paraffin':ti,ab,kw OR 'paraffin liquid':ti,ab,kw OR 'mineral oil':ti,ab,kw OR lubiprostone:ti,ab,kw OR amitiza:ti,ab,kw OR linaclotide:ti,ab,kw OR linzess:ti,ab,kw OR constella:ti,ab,kw OR prucalopride:ti,ab,kw OR resolor:ti,ab,kw OR motegrity:ti,ab,kw OR plecanatide:ti,ab,kw OR trulance:ti,ab,kw OR naronapride:ti,ab,kw OR 'ati-7505':ti,ab,kw) AND  (((‘randomized controlled trial’/de OR ‘controlled clinical trial’/de OR random*:ti,ab,tt OR ‘randomization’/de OR ‘intermethod comparison’/de OR placebo:ti,ab,tt OR (compare:ti,tt OR compared:ti,tt OR comparison:ti,tt) OR ((evaluated:ab OR evaluate:ab OR evaluating:ab OR assessed:ab OR assess:ab) AND (compare:ab OR compared:ab OR comparing:ab OR comparison:ab)) OR (open NEXT/1 label):ti,ab,tt OR ((double OR single OR doubly OR singly) NEXT/1 (blind OR blinded OR blindly)):ti,ab,tt OR ‘double blind procedure’/de OR (parallel NEXT/1 group*):ti,ab,tt OR (crossover:ti,ab,tt OR ‘cross over’:ti,ab,tt) OR ((assign* OR match OR matched OR allocation) NEAR/6 (alternate OR group OR groups OR intervention OR interventions OR patient OR patients OR subject OR subjects OR participant OR participants)):ti,ab,tt OR (assigned:ti,ab,tt OR allocated:ti,ab,tt) OR (controlled NEAR/8 (study OR design OR trial)):ti,ab,tt OR (volunteer:ti,ab,tt OR volunteers:ti,ab,tt) OR ‘human experiment’/de OR trial:ti,tt))) NOT ((((random* NEXT/1 sampl* NEAR/8 ('cross section***'** OR questionnaire* OR survey OR surveys OR database OR databases)):ti,ab,tt) NOT ('comparative study'/de OR 'controlled study'/de OR 'randomised controlled':ti,ab,tt OR 'randomized controlled':ti,ab,tt OR 'randomly assigned':ti,ab,tt)) OR ('cross‐sectional study' NOT ('randomized controlled trial'/de OR 'controlled clinical trial'/de OR 'controlled study'/de OR 'randomised controlled':ti,ab,tt OR 'randomized controlled':ti,ab,tt OR 'control group':ti,ab,tt OR 'control groups':ti,ab,tt)) OR ('case control*':ti,ab,tt AND random*:ti,ab,tt NOT ('randomised controlled':ti,ab,tt OR 'randomized controlled':ti,ab,tt)) OR ('systematic review':ti,tt NOT (trial:ti,tt OR study:ti,tt)) OR (nonrandom*:ti,ab,tt NOT random*:ti,ab,tt) OR 'random field*':ti,ab,tt OR ('random cluster' NEAR/4 sampl*):ti,ab,tt OR (review:ab AND review:it NOT trial:ti,tt) OR ('we searched':ab AND (review:ti,tt OR review:it)) OR 'update review':ab OR (databases NEAR/5 searched):ab OR ((rat:ti,tt OR rats:ti,tt OR mouse:ti,tt OR mice:ti,tt OR swine:ti,tt OR porcine:ti,tt OR murine:ti,tt OR sheep:ti,tt OR lambs:ti,tt OR pigs:ti,tt OR piglets:ti,tt OR rabbit:ti,tt OR rabbits:ti,tt OR cat:ti,tt OR cats:ti,tt OR dog:ti,tt OR dogs:ti,tt OR cattle:ti,tt OR bovine:ti,tt OR monkey:ti,tt OR monkeys:ti,tt OR trout:ti,tt OR marmoset*:ti,tt) AND 'animal experiment'/de) OR ('animal experiment'/de NOT ('human experiment'/de OR 'human'/de))) |
| **PUBMED**  [281 hits]^1^ | ("Child"[Mesh] OR "Adolescent"[Mesh] OR child*[Title/Abstract] OR adolescen*[Title/Abstract] OR youth[Title/Abstract] OR pediatric[Title/Abstract] OR toddler[Title/Abstract] OR neonat*[Title/Abstract] OR infant*[Title/Abstract] OR girl[Title/Abstract] or boy[Title/Abstract]) AND  ("Constipation"[Mesh] OR constipat*[Title/Abstract] OR costiveness[Title/Abstract] OR costive[Title/Abstract] OR obstipat*[Title/Abstract] OR "faecal impaction"[Title/Abstract] OR "delayed bowel movement"[Title/Abstract]) AND  (**"Probiotics"[Mesh] OR "Synbiotics"[Mesh]** OR **probiotic[Title/Abstract] OR synbiotic[Title/Abstract] OR lactobacillus[Title/Abstract] OR LGG[Title/Abstract] OR Lcr35[Title/Abstract] OR Bifidobacterium[Title/Abstract] OR Lactis[Title/Abstract] OR GCL2505[Title/Abstract] OR saccharomyces[Title/Abstract] OR longum[Title/Abstract] OR acidophilus[Title/Abstract] OR thermophilus[Title/Abstract] OR thermophylus[Title/Abstract] OR "Bacillus clausii"[Title/Abstract]** OR PEG[Title/Abstract] OR "polyethylene glycol"[Title/Abstract] OR macrogol[Title/Abstract] OR forlax[Title/Abstract] OR Miralax[Title/Abstract] OR Transipeg[Title/Abstract] OR Movicol[Title/Abstract] OR Forlax[Title/Abstract] OR Idrolax[Title/Abstract] OR GoLytely[Title/Abstract] OR PMF-100[Title/Abstract] OR Golitely[Title/Abstract] OR Nulitely[Title/Abstract] OR Fortrans[Title/Abstract] OR TriLyte[Title/Abstract] OR Colyte[Title/Abstract] OR moviprep[Title/Abstract] OR sorbitol[Title/Abstract] OR glucitol[Title/Abstract] OR mannitol[Title/Abstract] OR lactulose[Title/Abstract] OR lactitol[Title/Abstract] OR generlac[Title/Abstract] OR cephulac[Title/Abstract] OR cholac[Title/Abstract] OR constilac[Title/Abstract] OR enulose[Title/Abstract] OR cilac[Title/Abstract] OR heptalac[Title/Abstract] OR actilax[Title/Abstract] OR duphalac[Title/Abstract] OR Kristalose[Title/Abstract] OR "magnesium hydroxide"[Title/Abstract] OR "milk of magnesia"[Title/Abstract] OR "magnesium citrate"[Title/Abstract] OR "magnesium sulfate"[Title/Abstract] OR "magnesium salts"[Title/Abstract] OR "mg salts"[Title/Abstract] OR "magnesium oxide"[Title/Abstract] OR MgOH2[Title/Abstract] OR MgOH[Title/Abstract] OR MgO[Title/Abstract] OR MgSO4[Title/Abstract] OR citroma[Title/Abstract] OR diphenylmethane[Title/Abstract] OR "sodium picosulfate"[Title/Abstract] OR Bisacodyl[Title/Abstract] OR senokot[Title/Abstract] OR Dulcolax[Title/Abstract] OR anthraquinones[Title/Abstract] OR senna[Title/Abstract] OR sennosides[Title/Abstract] OR docusate[Title/Abstract] OR Colace[Title/Abstract] OR "liquid paraffin"[Title/Abstract] OR "paraffin liquid"[Title/Abstract] OR "mineral oil"[Title/Abstract] OR lubiprostone[Title/Abstract] OR Amitiza[Title/Abstract] OR linaclotide[Title/Abstract] OR Linzess[Title/Abstract] OR Constella[Title/Abstract] OR prucalopride[Title/Abstract] OR Resolor[Title/Abstract] OR Motegrity[Title/Abstract] OR plecanatide[Title/Abstract] OR Trulance[Title/Abstract] OR naronapride[Title/Abstract] OR "ATI-7505"[Title/Abstract]) AND  (randomized controlled trial[pt] OR controlled clinical trial[pt] OR randomized[tiab] OR placebo[tiab] OR clinical trials as topic[mesh:noexp] OR randomly[tiab] OR trial[ti] NOT (animals[mh] NOT humans [mh])) |

^1^as at June 18, 2022

## **Supplementary Table S3** Dataset used in the NMA of defecation frequency and treatment success.

| author | FU | mean_age | per_female | const_dur | basemean | trt | treat | nFU | nsuccess | meantype | n | mean | sd |
| --- | --- | --- | --- | --- | --- | --- | --- | --- | --- | --- | --- | --- | --- |
| Voskuijl 2004 | 8 | 6.50 | 46.00 |  | 2.59 | PEG | 1 | 46 | 26 | raw | 46 | 7.12 | 5.14 |
| Voskuijl 2004 | 8 | 6.50 | 44.00 |  | 2.75 | Lactulose | 2 | 45 | 13 | raw | 45 | 6.43 | 5.18 |
| Banaszkewicz 2005 | 4 | 6.60 |  | 24.00 | 2.20 | Lactulose+Probiotics | 1 | 43 | 31 | raw | 43 | 5.90 | 2.30 |
| Banaszkewicz 2005 | 4 | 5.42 |  | 23.00 | 2.20 | Lactulose | 2 | 41 | 28 | raw | 41 | 7.70 | 5.40 |
| Dupont 2005 - babies | 6 | 2.33 | 57.00 |  |  | PEG | 1 |  |  | raw | 10 | 8.67 | 4.73 |
| Dupont 2005 - babies | 6 | 2.15 | 36.00 |  |  | Lactulose | 2 |  |  | raw | 12 | 9.67 | 5.03 |
| Dupont 2005 - toddlers | 6 | 2.33 | 57.00 |  |  | PEG | 1 |  |  | raw | 41 | 8.00 | 3.07 |
| Dupont 2005 - toddlers | 6 | 2.15 | 36.00 |  |  | Lactulose | 2 |  |  | raw | 33 | 6.00 | 1.55 |
| Urganci 2005 | 8 | 3.84 | 40.00 | 9.50 | 1.90 | Mineral oil | 1 | 19 | 19 | raw | 20 | 16.10 | 2.20 |
| Urganci 2005 | 8 | 3.64 | 50.00 | 9.20 | 1.80 | Lactulose | 2 | 12 | 8 | raw | 20 | 12.30 | 6.60 |
| Loening Baucke 2006 | 4 | 8.00 | 21.00 |  | 3.50 | PEG | 1 | 38 | 19 | raw | 38 | 9.70 | 5.60 |
| Loening Baucke 2006 | 4 | 8.20 | 15.00 |  | 3.50 | Magnesium Hydroxide | 2 | 37 | 22 | raw | 37 | 9.70 | 6.00 |
| Bu 2007 | 4 | 3.06 | 55.56 | 21.90 | 0.57 | Probiotics | 1 | 18 | 14 | raw | 18 | 5.03 | 1.50 |
| Bu 2007 | 4 | 2.92 | 80.00 | 22.80 | 0.37 | Placebo | 2 | 9 | 1 | raw | 9 | 2.60 | 0.71 |
| Bu 2007 | 4 | 2.70 | 50.00 | 20.90 | 0.55 | Magnesium Oxide | 3 | 18 | 13 | raw | 18 | 4.75 | 1.11 |
| Farahmand 2007 | 8 | 4.10 | 33.39 | 24.00 | 1.60 | Mineral oil | 1 | 127 | 108 | raw | 127 | 13.10 | 2.30 |
| Farahmand 2007 | 8 | 4.20 | 38.41 | 22.00 | 1.80 | Lactulose | 2 | 120 | 35 | raw | 120 | 8.10 | 3.10 |
| Hannah 2008 | 1 | 3.64 | 50.00 |  |  | Probiotics | 1 | 22 | 17 | raw | 22 | 2.80 | 2.30 |
| Hannah 2008 | 1 | 3.52 | 47.37 |  |  | Placebo | 2 | 19 | 7 | raw | 19 | 1.70 | 0.80 |
| Nurko 2008 | 2 | 8.40 | 38.50 | 60.00 | 1.50 | PEG | 1 | 24 | 20 | raw | 24 | 5.95 | 4.90 |
| Nurko 2008 | 2 | 8.00 | 37.50 | 49.20 | 1.60 | Placebo | 2 | 20 | 10 | raw | 20 | 2.14 | 2.82 |
| Karami 2009 | 4 | 4.01 | 48.54 | 30.70 | 2.70 | PEG | 1 |  |  | raw | 48 | 4.70 | 1.80 |
| Karami 2009 | 4 | 4.01 | 48.54 | 24.70 | 3.00 | Mineral oil | 2 |  |  | raw | 55 | 4.50 | 1.90 |
| Ratanamongkol 2009 | 4 | 2.58 | 32.61 | 12.00 | 3.00 | PEG | 1 | 46 | 42 | raw | 46 | 5.92 | 1.72 |
| Ratanamongkol 2009 | 4 | 2.58 | 48.84 | 12.00 | 3.33 | Magnesium Hydroxide | 2 | 43 | 28 | raw | 43 | 5.33 | 2.30 |
| Coccorullo 2010 | 8 | 0.68 | 63.64 |  |  | Probiotics | 1 | 22 | 22 | TS only |  |  |  |
| Coccorullo 2010 | 8 | 0.73 | 27.27 |  |  | Placebo | 2 | 22 | 15 | TS only |  |  |  |
| Khodadad 2010 | 4 | 6.20 | 51.61 |  | 2.19 | Probiotics | 1 | 31 | 22 | raw | 31 | 5.22 | 3.20 |
| Khodadad 2010 | 4 | 5.90 | 51.35 |  | 1.83 | Mineral oil+Probiotics | 2 | 37 | 28 | raw | 37 | 7.49 | 4.40 |
| Khodadad 2010 | 4 | 6.90 | 55.17 |  | 1.81 | Mineral oil | 3 | 29 | 24 | raw | 29 | 6.75 | 2.60 |
| Guerra 2011 | 5 |  | 80.00 |  |  | Probiotics | 1 |  |  | change | 29 | 0.14 | 0.76 |
| Guerra 2011 | 5 |  | 79.31 |  |  | Placebo | 2 |  |  | change | 30 | -0.47 | 0.89 |
| Gomes 2011 | 8 | 4.37 | 41.20 |  | 2.00 | PEG | 1 |  |  | raw | 17 | 5.00 | 1.56 |
| Gomes 2011 | 8 | 5.05 | 38.10 |  | 1.33 | Magnesium Hydroxide | 2 |  |  | raw | 21 | 4.31 | 1.89 |
| Rafati 2011 | 4 | 4.40 | 48.75 |  | 1.60 | PEG | 1 |  |  | raw | 80 | 7.00 | 3.80 |
| Rafati 2011 | 4 | 4.22 | 44.87 |  | 1.40 | Mineral oil | 2 |  |  | raw | 78 | 6.30 | 3.10 |
| Tabbers 2011 | 3 | 7.00 | 46.84 | 40.80 | 1.50 | Probiotics | 1 | 71 | 27 | raw | 74 | 2.90 | 3.20 |
| Tabbers 2011 | 3 | 6.50 | 48.75 | 40.80 | 1.50 | Placebo | 2 | 72 | 17 | raw | 74 | 2.60 | 2.60 |
| Saneian 2012 | 2 | 3.26 |  | 20.86 | 1.33 | PEG | 1 |  |  | change | 30 | 3.56 | 1.99 |
| Saneian 2012 | 2 | 3.19 |  | 20.64 | 1.53 | Lactulose | 2 |  |  | change | 30 | 3.16 | 1.72 |
| Saneian 2012 | 2 | 3.11 |  | 16.29 | 1.37 | Magnesium Hydroxide | 3 |  |  | change | 30 | 4.67 | 2.29 |
| Wang 2012 | 2 | 11.29 | 59.05 |  |  | PEG | 1 | 105 | 76 | TS only |  |  |  |
| Wang 2012 | 2 | 11.20 | 57.66 |  |  | Lactulose | 2 | 111 | 46 | TS only |  |  |  |
| Olgac 2013 | 4 |  |  |  | 2.00 | Probiotics | 1 |  |  | raw | 25 | 5.00 | 0.30 |
| Olgac 2013 | 4 |  |  |  | 1.70 | Lactulose | 2 |  |  | raw | 28 | 4.60 | 0.40 |
| Mugie 2014 | 8 | 8.30 | 56.60 | 52.80 | 0.80 | Prucalopride | 1 | 106 | 18 | change | 106 | 1.50 | 2.35 |
| Mugie 2014 | 8 | 8.20 | 54.21 | 50.40 | 1.10 | Placebo | 2 | 107 | 19 | change | 107 | 1.00 | 1.78 |
| Sadeghzadeh 2014 | 4 | 6.10 | 41.67 |  |  | Lactulose+Probiotics | 1 |  |  | change | 24 | 2.08 | 0.65 |
| Sadeghzadeh 2014 | 4 | 6.30 | 58.33 |  |  | Lactulose | 2 |  |  | change | 24 | 1.54 | 0.98 |
| Treepongkaruna 2014 | 4 | 1.98 | 45.45 | 11.10 | 0.70 | Lactulose | 1 |  |  | raw | 44 | 5.60 | 2.87 |
| Treepongkaruna 2014 | 4 | 1.99 | 40.91 | 10.80 | 0.50 | PEG | 2 |  |  | raw | 43 | 7.70 | 3.85 |
| Ala 2015 |  | 6.50 | 42.00 | 26.40 |  | PEG | 1 | 100 | 70 | TS only |  |  |  |
| Ala 2015 |  | 6.50 | 55.00 | 37.20 |  | PEG+Lactulose | 2 | 100 | 87 | TS only |  |  |  |
| Hashemi 2015 | 6 |  |  |  |  | PEG+Probiotics | 1 |  |  | raw | 40 | 4.23 | 1.41 |
| Hashemi 2015 | 6 |  |  |  |  | Probiotics | 2 |  |  | raw | 40 | 3.40 | 1.22 |
| Hashemi 2015 | 6 |  |  |  |  | PEG | 3 |  |  | raw | 40 | 3.48 | 1.06 |
| Basturk 2017 | 4 | 9.31 | 55.56 |  |  | Probiotics | 1 | 72 | 48 | TS only |  |  |  |
| Basturk 2017 | 4 | 9.06 | 54.05 |  |  | Placebo | 2 | 74 | 21 | TS only |  |  |  |
| Mahdavi 2017 | 4 | 3.50 |  |  | 1.78 | PEG+Probiotics | 1 |  |  | raw | 37 | 6.31 | 2.05 |
| Mahdavi 2017 | 4 | 4.00 |  |  | 2.02 | PEG | 2 |  |  | raw | 41 | 6.53 | 2.50 |
| Russo 2017 | 4 | 7.40 | 51.85 |  | 2.30 | PEG+Probiotics | 1 | 25 | 20 | raw | 25 | 6.00 | 1.20 |
| Russo 2017 | 4 | 7.10 | 53.57 |  | 2.50 | PEG | 2 | 25 | 16 | raw | 25 | 6.30 | 0.90 |
| Torabi 2017 |  | 5.24 | 57.50 |  |  | PEG | 1 | 80 | 40 | TS only |  |  |  |
| Torabi 2017 |  | 5.28 | 56.25 |  |  | Mineral oil | 2 | 80 | 47 | TS only |  |  |  |
| Wojtyniak 2017 | 4 | 3.23 | 54.17 | 14.70 |  | Probiotics | 1 | 41 | 24 | raw | 41 | 4.50 | 3.00 |
| Wojtyniak 2017 | 4 | 3.11 | 56.52 | 13.80 |  | Placebo | 2 | 40 | 28 | raw | 40 | 6.70 | 3.80 |
| Cao 2018 | 6 | 3.90 | 44.00 | 9.80 | 4.20 | Lactulose | 1 |  |  | change | 46 | 0.50 | 0.87 |
| Cao 2018 | 6 | 4.00 | 48.00 | 10.10 | 4.90 | Placebo | 2 |  |  | change | 45 | 0.20 | 0.86 |
| Jadrešin 2018 | 6 | 4.40 | 66.67 |  |  | Lactulose+Probiotics | 1 | 18 | 7 | raw | 18 | 6.50 | 0.50 |
| Jadrešin 2018 | 6 | 4.70 | 73.33 |  |  | Lactulose | 2 | 15 | 6 | raw | 15 | 6.60 | 0.80 |
| Jose 2018 | 4 |  | 40.00 |  |  | Lactulose+Probiotics | 1 |  |  | change | 25 | 0.89 | 0.69 |
| Jose 2018 | 4 |  | 60.00 |  |  | Lactulose | 2 |  |  | change | 25 | 0.71 | 0.62 |
| Modin 2018 | 24 | 6.80 | 36.70 | 19.00 | 5.40 | PEG | 1 | 47 | 33 | raw | 49 | 9.10 | 3.30 |
| Modin 2018 | 24 | 7.33 | 50.90 | 24.50 | 5.20 | Placebo | 2 | 48 | 19 | raw | 53 | 8.60 | 3.40 |
| Wegner 2018 | 4 | 4.66 | 44.62 | 23.23 | 1.75 | PEG+Probiotics | 1 | 59 | 57 | raw | 59 | 7.69 | 4.30 |
| Wegner 2018 | 4 | 4.69 | 43.75 | 23.05 | 1.77 | PEG | 2 | 61 | 59 | raw | 61 | 7.74 | 3.60 |
| Cassettari 2019 | 8 | 8.78 | 43.75 | 73.33 |  | PEG | 1 | 16 | 10 | TS only |  |  |  |
| Cassettari 2019 | 8 | 8.92 | 58.82 | 74.00 |  | Sodium Picosulfate | 2 | 17 | 8 | TS only |  |  |  |
| Jarzebicka 2019 | 4 | 3.65 | 50.98 | 20.36 | 2.19 | PEG | 1 | 46 | 46 | raw | 46 | 8.50 | 0.70 |
| Jarzebicka 2019 | 4 | 3.60 | 37.25 | 18.33 | 2.08 | Lactulose | 2 | 49 | 39 | raw | 49 | 5.50 | 0.40 |
| Kubota 2020 | 4 | 2.73 | 45.00 |  |  | Probiotics | 1 |  |  | change | 20 | 2.38 | 0.53 |
| Kubota 2020 | 4 | 3.36 | 47.37 |  |  | Probiotics+Magnesium Oxide | 2 |  |  | change | 19 | 2.38 | 0.55 |
| Kubota 2020 | 4 | 2.85 | 42.86 |  |  | Magnesium Oxide | 3 |  |  | change | 21 | 2.38 | 0.52 |
| Tjokronegoro 2020 | 4 | 7.00 | 64.10 |  | 4.49 | Probiotics | 1 |  |  | raw | 39 | 6.92 | 3.31 |
| Tjokronegoro 2020 | 4 | 7.00 | 64.10 |  | 5.15 | Placebo | 2 |  |  | raw | 39 | 6.31 | 2.71 |
| Dheivamani 2021 | 4 | 5.13 | 46.00 | 13.89 |  | PEG | 1 |  |  | raw | 47 | 7.98 | 4.63 |
| Dheivamani 2021 | 4 | 5.16 | 52.00 | 27.66 |  | Lactulose | 2 |  |  | raw | 45 | 6.53 | 5.37 |
| Worona-Dibner 2021 | 4 | 1.77 | 53.66 |  | 1.77 | PEG | 1 | 39 | 33 | change | 39 | 10.48 | 6.22 |
| Worona-Dibner 2021 | 4 | 1.91 | 57.14 |  | 1.91 | Magnesium Hydroxide | 2 | 41 | 33 | change | 41 | 11.87 | 5.86 |
| Benninga 2022 | 14 | 11.20 | 54.14 |  | 1.40 | Lubiprostone | 1 | 399 | 74 | TS only |  |  |  |
| Benninga 2022 | 14 | 11.20 | 54.36 |  | 1.40 | Placebo | 2 | 195 | 28 | TS only |  |  |  |
| Foroughi 2022 | 3 | 4.03 | 44.44 | 3.42 |  | PEG | 1 |  |  | raw | 36 | 6.58 | 0.60 |
| Foroughi 2022 | 3 | 5.56 | 55.55 | 3.58 |  | PEG+Probiotics | 2 |  |  | raw | 36 | 6.72 | 0.51 |
| Gan 2022 | 4 | 8.40 |  |  |  | Probiotics | 1 |  |  | raw | 44 | 3.55 | 1.37 |
| Gan 2022 | 4 | 8.10 |  |  |  | Placebo | 2 |  |  | raw | 40 | 3.09 | 1.34 |
| Lee 2022 - 2 wk | 2 | 3.22 | 42.00 | 9.10 | 2.70 | Probiotics | 1 |  |  | raw | 37 | 3.69 | 2.60 |
| Lee 2022 - 2 wk | 2 | 3.70 | 64.71 | 7.60 | 2.90 | Lactulose+Probiotics | 2 |  |  | raw | 61 | 4.57 | 2.91 |
| Lee 2022 - 2 wk | 2 | 3.53 | 52.17 | 11.40 | 2.70 | Lactulose | 3 |  |  | raw | 60 | 4.05 | 2.49 |
| Lee 2022 - 6 wk | 6 | 3.22 | 42.00 | 9.10 |  | Probiotics | 1 | 37 | 12 |  |  |  |  |
| Lee 2022 - 6 wk | 6 | 3.70 | 64.71 | 7.60 |  | Lactulose+Probiotics | 2 | 61 | 36 |  |  |  |  |
| Lee 2022 - 6 wk | 6 | 3.53 | 52.17 | 11.40 |  | Lactulose | 3 | 60 | 40 |  |  |  |  |
| Mansour 2022 - 4 wk | 4 | 4.16 | 53.49 | 11.02 | 2.09 | PEG | 1 |  |  | raw | 20 | 3.23 | 0.50 |
| Mansour 2022 - 4 wk | 4 | 4.16 | 53.49 | 11.02 | 2.09 | Lactulose | 2 |  |  | raw | 22 | 3.46 | 0.70 |
| Mansour 2022 - 12 wk | 12 | 4.16 | 53.49 | 11.02 |  | PEG | 1 | 20 | 19 | TS only |  |  |  |
| Mansour 2022 - 12 wk | 12 | 4.16 | 53.49 | 11.02 |  | Lactulose | 2 | 22 | 17 | TS only |  |  |  |
| Lojantorn 2023 | 4 | 2.70 | 65.00 | 12.10 | 4.00 | Probiotics | 1 | 18 | 10 | raw | 20 | 4.30 | 1.80 |
| Lojantorn 2023 | 4 | 2.70 | 44.00 | 12.70 | 3.00 | Placebo | 2 | 20 | 9 | raw | 18 | 4.20 | 1.70 |

FU, follow-up; MgO, magnesium oxide; nFU, number of participants observed at follow-up; nsuccess, number of participants at follow-up who achieved treatment success PEG, polyethylene glycol; per, percent; sd, standard deviation; trt, treatment; TS, treatment success; wk, weeks; y, years.

## **Supplementary Table S4** Code used in the NMA of defecation frequency and treatment success in R software. This code will run after saving the dataset from Supplementary Table S3 in an excel file as ‘Dataset’ in the working directory as an excel file.

| Operation | R code used | | |
| --- | --- | --- | --- |
| Opening the packages used | library(tidyverse)  library(netmeta)  library(readxl) | | |
| Importing the dataset  And deleting transitivity variables not needed for meta-analysis | dat <- read_excel("Dataset.xlsx")  dat <- subset(dat, select=-c(FU, mean_age,per_female,const_dur, basemean)) | | |
| Rearrange the dataset | dat <-dat %>%  pivot_wider(  names_from = treat,  names_sep=".",  values_from = c(trt,nFU, nsuccess,n, mean, sd)) | | |
| The code used to set up the data and run the network meta-analysis was run separately to get results for both defecation frequency and treatment success. Then all following code was the same.  Set-up the dataset as required for meta-analysis and keep only desired variables. | Defecation frequency | Treatment success | |
|  | p1 <- pairwise(list(trt.1, trt.2,trt.3),  n = list(n.1, n.2,n.3),  mean = list(mean.1,mean.2,mean.3),  sd = list(sd.1, sd.2,sd.3),  data = dat, studlab = author)  p1 <- subset(p1, select=c(TE, seTE,n1,n2,studlab,treat1,treat2)) | p1 <- pairwise(list(trt.1, trt.2,trt.3),  n = list(nFU.1, nFU.2, nFU.3),  event=list(nsuccess.1, nsuccess.2, nsuccess.3),  data = dat, studlab = author)  p1 <- subset(p1, select=c(TE, seTE,n1,n2,studlab,treat1,treat2)) | |
| Run the meta-analysis and rank the treatments based on P-scores | net1 <- netmeta(TE, seTE, treat1, treat2, studlab, n1=n1,n2=n2,  data = p1, reference.group ="Probiotics", random=T, method.tau="REML", sm = "MD")  netrank(net1, small.values="bad", method="P-score", random=T) | net1 <- netmeta(TE, seTE, treat1, treat2, studlab, n1=n1,n2=n2,data = p1, reference.group ="Probiotics", random=T, method.tau="REML", sm = "RR") | |
| Generate network plot | netgraph(net1, plastic=F, cex=1.5, number.of.studies=F, lwd=3,  seq="o", points=T,  cex.points= n.trts,  col.points="cornflowerblue",  labels=paste0(trts,"  n=",n.trts)) | | |
| Generate forest plot | forest(net1, pooled="random", col.by = "black", addrow.subgroups = T,  fontsize = 10, spacing = 0.7, squaresize = 0.9, reference.group="Probiotics",  overall.hetstat=F,  digits=2,  small.values="undesirable",  sortvar=-Pscore,  drop.reference.group = F,  rightcols=c("Pscore", "effect", "ci"),  leftcols=c("studlab","n.trts", "k"),  label.left = "Favours Probiotics",  label.right = "Favours Other", smlab=" ",xlab="BMs/wk") | | |
| Funnel plot | order <- c("Placebo", "Magnesium Hydroxide", "Magnesium Oxide", "Mineral oil", "Lactulose", "PEG", "Probiotics","Probiotics+Magnesium Oxide","Mineral oil+Probiotics", "PEG+Probiotics", "Lactulose+Probiotics", "Prucalopride")  funnel(net1,order=order, xlab="Mean Difference",method.bias="Egger", legend=F) | | order <- c("Placebo","Magnesium Hydroxide", "Magnesium Oxide", "Mineral oil", "Lactulose","Sodium Picosulfate", "PEG", "Probiotics", "Lactulose+Probiotics", "Mineral oil+Probiotics", "PEG+Lactulose", "PEG+Probiotics", "Prucalopride", "Lubiprostone")  funnel(net1,order=order, xlab="Relative Risk", method.bias="Egger", legend=F) |
| Global inconsistency  Netsplit | global <- decomp.design(net1)  ns1 <-netsplit(net1,method="SIDDE") | | |
| Generate league table | a <- netrank(net1, small.values="bad", method="P-score", random=T)  netleague(net1,verbose = FALSE, common=F,seq=a,  small="bad",digits=2) | | |

## **Supplementary Table S5** Trial protocols identified and their publication status.

| Protocols from trial registries | Published version | Identified by search? | Included in review? |
| --- | --- | --- | --- |
| <https://trialsearch.who.int/Trial2.aspx?TrialID=IRCT2013041513021N1> | Abediny et al. (2016)^S1^ | Yes | Yes |
| <https://trialsearch.who.int/Trial2.aspx?TrialID=NTR1098>  <https://clinicaltrials.gov/ct2/show/record/NCT01810653> | Bekkali et al. (2018)^S2^ | Yes | No |
| <https://trialsearch.who.int/Trial2.aspx?TrialID=EUCTR2013-003468-30-BE>  <https://clinicaltrials.gov/ct2/show/results/NCT02042183> | Benninga et al. (2022)^S3^ | Yes | Yes |
| <https://clinicaltrials.gov/ct2/show/NCT00404040> | Candy et al. (2006)^S4^ | Yes | No |
| <https://trialsearch.who.int/Trial2.aspx?TrialID=ChiCTR2000038603>  <http://www.chictr.org.cn/showproj.aspx?proj=62068> | Gan et al. (2022)^S5^ | No, published after search | Yes |
| IRCT20181214041966N1  <https://en.irct.ir/trial/35951> | Hakimzadeh et al. (2019^S6^ | Yes | No |
| <https://trialsearch.who.int/Trial2.aspx?TrialID=IRCT2013112415511N1> | Hashemi et al. (2015^S7^ | Yes | Yes |
| <https://clinicaltrials.gov/ct2/show/record/NCT01587846> | Jadresin et al. (2018^S8^ | Yes | Yes |
| <https://clinicaltrials.gov/ct2/show/NCT03177434> | Jarzebicka et al. (2019)^S9^ | Yes | Yes |
| <https://en.irct.ir/trial/2151> | Kasiri et al. (2020)^S10^ | Yes | No |
| https://trialsearch.who.int/Trial2.aspx?TrialID=IRCT138811193309N1 | Khodadad 2010^S11^ | Yes |  |
| Kubota  <https://trialsearch.who.int/Trial2.aspx?TrialID=JPRN-UMIN000033922> | Kubota 2020^S12^ | Yes | Yes |
| <https://trialsearch.who.int/Trial2.aspx?TrialID=TCTR20200222003> | Lojanatorn 2023^S13^ | Yes | Yes |
| <https://trialsearch.who.int/Trial2.aspx?TrialID=IRCT2015072723363N1> | Mahdavi 2017^S14^ | Yes | Yes |
| <https://clinicaltrials.gov/ct2/show/NCT01566409> | Modin 2018^S15^ | Yes |  |
| <https://clinicaltrials.gov/ct2/show/NCT01330381> | Mugie 2014^S16^ | Yes | Yes |
| <https://trialsearch.who.int/Trial2.aspx?TrialID=IRCT201207311579N1>  http://www.irct.ir/searchresult.php? Id=1579&number=1 | Saneian 2013^S17^ | Yes | No |
| <https://clinicaltrials.gov/ct2/show/study/NCT01592734> | Savino 2012^S18^ | Yes | No |
| <https://trialsearch.who.int/Trial2.aspx?TrialID=NTR1571> | Tabbers 2011^S19^ | Yes | Yes |
| <https://clinicaltrials.gov/ct2/show/NCT00403819> | Thomson 2007^S20^ | Yes | Yes |
| <https://clinicaltrials.gov/ct2/show/NCT00255372> | Treepongkaruna 2014^S21^ | Yes | Yes |
| <https://trialsearch.who.int/Trial2.aspx?TrialID=JPRN-UMIN000044070> | Ongoing |  |  |
| <https://trialsearch.who.int/Trial2.aspx?TrialID=IRCT20120215009014N361> | Ongoing |  |  |
| <https://clinicaltrials.gov/ct2/show/study/NCT04026113> | Ongoing |  |  |
| <https://trialsearch.who.int/Trial2.aspx?TrialID=IRCT20210525051398N1> | Ongoing |  |  |
| <https://trialsearch.who.int/Trial2.aspx?TrialID=IRCT20190717044239N1> | Ongoing |  |  |
| <https://trialsearch.who.int/Trial2.aspx?TrialID=CTRI/2018/01/011262> | Ongoing |  |  |
| <https://trialsearch.who.int/Trial2.aspx?TrialID=CTRI/2018/01/011061> | Ongoing |  |  |
| <https://trialsearch.who.int/Trial2.aspx?TrialID=IRCT20160827029535N7> | Ongoing |  |  |
| <https://trialsearch.who.int/Trial2.aspx?TrialID=CTRI/2022/01/039517> | Ongoing |  |  |
| <https://trialsearch.who.int/Trial2.aspx?TrialID=TCTR202103110010> | Ongoing |  |  |
| <https://trialsearch.who.int/Trial2.aspx?TrialID=KCT0006286> | Ongoing |  |  |
| <https://trialsearch.who.int/Trial2.aspx?TrialID=CTRI/2022/02/040158> | Ongoing |  |  |
| <https://trialsearch.who.int/Trial2.aspx?TrialID=IRCT20150706023084N13> | Ongoing |  |  |
| https://trialsearch.who.int/Trial2.aspx?TrialID=EUCTR2019-001955-38-BG | Ongoing |  |  |
| <https://trialsearch.who.int/Trial2.aspx?TrialID=EUCTR2019-001500-38-PL> | Ongoing |  |  |
| <https://clinicaltrials.gov/ct2/show/study/NCT03054805> | Complete, results reported in protocol record but not published yet |  |  |
| <https://clinicaltrials.gov/ct2/show/results/NCT04110145> | Complete, results reported in protocol record but not published yet |  |  |
| <https://clinicaltrials.gov/ct2/show/results/NCT02559570> | Complete, results reported in protocol record but not published yet |  |  |
| <https://clinicaltrials.gov/show/NCT04759833> | Recruitment taking place |  |  |
| <https://clinicaltrials.gov/ct2/show/study/NCT03120520> | Complete, not published |  |  |
| <https://clinicaltrials.gov/ct2/show/NCT00153114> | Data collection complete, no results published |  |  |
| <https://trialsearch.who.int/Trial2.aspx?TrialID=IRCT2013120415530N3> | Data collection complete, no results published |  |  |
| <https://clinicaltrials.gov/show/NCT00153114> | No updates |  |  |
| <https://clinicaltrials.gov/ct2/show/record/NCT03030664> | Data collection complete, no results published |  |  |
| <https://trialsearch.who.int/Trial2.aspx?TrialID=IRCT201409015330N7> | No updates |  |  |
| <https://trialsearch.who.int/Trial2.aspx?TrialID=IRCT2014091618971N2> | No updates |  |  |
| <https://clinicaltrials.gov/ct2/show/NCT01913665> | No updates |  |  |
| <https://clinicaltrials.gov/ct2/show/NCT01629147> | Abandoned |  |  |
| <https://clinicaltrials.gov/ct2/show/study/NCT01388712> | Abandoned |  |  |
| Bekkali Forlax versus Transipeg in constipated children (2007)  Netherland Trial Register: TC=1098 | The Netherlands Trial Registery is no longer operational, the details cannot be retrieved. |  |  |

## **Supplementary Table S6** List of the 77 reports excluded during full text screening (with reasons).

| Author, year | Reason for exclusion |
| --- | --- |
| Connolly et al. (1975)^S22^ | Participants aged >18 years. |
| Perkin et al. (1977^S23^ | Did not provide FC definition. |
| Bass et al. (1981)^S24^ | Participants aged >18 years. |
| Sondheimer et al. (1982)^S25^ | The participants were assigned to treatment groups non-randomly. Patients were assigned according to the last digit of their hospital number. |
| Gallet et al. (1990)^S26^ | This study had no comparison group. All 40 children received lactitol. |
| Martino et al. (1992)^S27^ | Did not provide FC definition. |
| Pitzalis et al. (1995)^S28^ | The definition of FC was too dissimilar to the Rome criteria to be included. |
| Muller et al. (1994)^S29^ | Full text not found. |
| Dupont et al. (2000)^S30^ | Conference abstract of subsequently published study. The full text version of this study was identified by the systematic search, but not included in the review.^S31^ |
| Gremse et al. (2000)^S32^ | Conference abstract of subsequently published RCT. The full-text version was identified by the systematic search (Gremse et al. 2002^S33^ below), but not included in the review. |
| Tozzi et al. (2000)^S34^ | Conference abstract. We could not identify the full text of this study. |
| Gremse et al. (2002)^S33^ | Definition of functional constipation was not reported. |
| Loening-Baucke et al. (2002)^S35^ | Children were not randomized to treatment groups (PEG or milk of magnesia). |
| Sloots et al. (2002)^S36^ | Participants aged >18 years. |
| Candy et al. (2003)^S37^ | Conference abstract. Conference abstract of subsequently published study.^S4^ After identifying the full text, the study was not included because treatments for Disimpaction were evaluated instead of maintenance therapies. |
| Voskuijl et al. (2003)^S38^ | Conference abstract of subsequently published RCT. The full text version of this study was identified by the systematic search and included in the review (Voskuijl 2004^S39^). |
| Kinservik et al. (2004)^S40^ | Commentary article. |
| Shevtsov et al. (2005)^S41^ | Participants aged >18 years. |
| Dupont et al. (2006)^S31^ | No control group |
| Bekkali et al. (2007)^S42^ | This study is a pilot study with only one treatment group (probiotic mixture). |
| Uhm et al. (2007)^S43^ | Not stated if the study was randomized. |
| Camilleri et al. (2008)^S44^ | This RCT evaluated prucalopride compared to a placebo in an adult population. |
| Coccorullo et al. (2009)^S45^ | Conference abstract. |
| Hyman et al. (2009)^S46^ | Conference abstract of subsequently published study. The full text version of this study was identified by the systematic search, but not included in the review.^S47^ |
| Quigley et al. (2009)^S48^ | Participants aged >18 years. |
| Tack et al. (2009)^S49^ | This RCT only evaluated the effect of Prucalopride on adult participants with constipation. |
| Bae et al. (2010)^S50^ | This was a retrospective study with was no comparison group of interest because all children received lactulose. The exposure of interest was fluid intake (also not randomized). |
| Camilleri et al. (2010)^S51^ | A follow-up of the RCT from the same authors (Camilleri 2008) of an adult population comparing prucalopride to a placebo. |
| Farahmand et al. (2010)^S52^ | This RCT compared liquid parrafin administered orally to a rectal administration of liquid parrafin. |
| Lembo et al. (2010)^S53^ | Participants aged >18 years. |
| Lembo et al. (2011)^S54^ | Participants aged >18 years. |
| Tabbers et al. (2010)^S55^ | Conference abstract. The published full-text of this trial was identified by our systematic search and included in the review (Tabbers 2011^S19^). |
| Tabbers et al. (2011)^S56^ | This was a pilot study evaluating the effect of probiotics on childhood constipation with no control group. |
| Gheibi et al. (2012)^S57^ | Participants were not correctly randomized to treatment groups. After randomization, but before treatment began, participants were reassigned to the opposite treatment group if they had already tried and not responded to the treatment they were initially randomized to. |
| Parzȩcka et al. (2012)^S58^ | This was a retrospective study and participants were not randomized to treatment groups. |
| Savino et al. (2012)^S59^ | There was no control group of interest. All participants received PEG either with or without electrolytes. |
| Cinca et al. (2013)^S60^ | Participants aged >18 years. |
| Saneian et al. (2013)^S17^ | Children were not randomized to treatment groups. The authors used ‘alternate randomisation’ which is not a truly random method of allocation. |
| Treepongkaruna et al. (2013)^S61^ | Conference abstract |
| Winter et al. (2013)^S62^ | There was only one treatment group. All patients received Prucalopride. |
| Benninga et al. (2014)^S63^ | Conference abstract. |
| Dziechciarz et al. (2014)^S64^ | All participants were allocated to the same treatment group. |
| Hyman et al. (2014) | There was no control group, all participants received Lubiprostone at different doses. |
| Magro et al. (2014)^S65^ | Participants aged >18 years. |
| Tack et al. (2014)^S66^ | Participants aged >18 years. |
| Dziechciarz et al. (2015)^S67^ | No control group. |
| Russo et al. (2015)^S68^ | Conference abstract of subsequently published RCT. The full text version of this study was identified by the systematic search and included in the review (Russo 2017)^S69^. |
| Wegner et al. (2015)^S70^ | Conference abstract of subsequently published RCT. The full text version of this study was identified by the systematic search and included in the review (Wegner 2018^S71^). |
| Baştürk et al. (2016)^S72^ | This report is a conference abstract of a published RCT identified by our systematic search (See below). |
| Baştürk et al. (2016)^S73^ | This RCT compared synbiotics to a placebo, however, children were diagnosed with irritable bowel syndrome according to the Rome III criteria instead of FC. |
| Chao et al. (2016)^S74^ | Conference abstract. The full text of this study could not be identified. |
| Jordan-Ely et al. (2016)^S75^ | Conference abstract. The full text of this study could not be identified. |
| Jarzebicka et al. (2017)^S76^ | Conference abstract. The published full-text of this trial was identified by our systematic search and included in the review (Jarzebicka 2019^S9^). |
| Jarzebicka et al. (2017)^S77^ | Another conference abstract of the same trial as above. |
| Miner et al. (2017)^S78^ | Participants aged >18 years. |
| Modin et al. (2017)^S79^ | Conference abstract of a published RCT. The full text article of the RCT was published and identified by our systematic search and included in our review (Modin 2018^S15^). |
| Wojtyniak et al. (2017)^S80^ | Conference abstract of a published RCT. The full text article of the RCT was published and identified by our systematic search and included in our review (Wojtyniak 2017).^S81^ |
| Acharyya et al. (2018)^S82^ | This RCT did not have an adequate control. The two interventions compared were PEG in combination with electrolytes compared to PEG alone. This report is a conference abstract and included in the search for a full text (see Table below). |
| Benninga et al. (2018)^S83^ | This is a conference abstract for an RCT which was identified by our systematic search and included in the review (Benninga 2022^S3^). |
| Benninga et al. (2018)^S84^ | Conference abstract. |
| Kubota et al. (2018)^S85^ | Conference abstract of a published RCT identified by our systematic search. The full text of the RCT met the inclusion criteria and was included in the review (Kubota 2020^S12^). |
| Kumar et al. (2018)^S86^ | Conference abstract |
| Xinias et al. (2018)^S87^ | This study was not randomized. The parents and pediatrician of the infant decided which group the infant would be assigned to. |
| Di Lorenzo et al. (2019)^S88^ | Conference abstract |
| Hakimzadeh et al. (2019)^S89^ | This study evaluated children with occult constipation, where there are no constipation symptoms except having a hard stool consistency or fecal impaction. |
| Matthew et al. (2019)^S90^ | Commentary article. |
| Shatnawi et al. (2019)^S91^ | This study compared PEG and lactulose for disimpaction therapy instead of maintenance therapy. |
| Bae et al. (2020)^S92^ | This study was an observational retrospective study which aimed to evaluate the usefulness of classifying subtypes of FC using a colon transit time test. PEG 4000 or lactulose were the treatments administered, but they were not administered randomly to participants, given the retrospective nature of the study. |
| Demir et al. (2020)^S93^ | Not randomized. |
| Kasiri et al. (2020)^S10^ | No functional constipation definition was reported. |
| Olgac et al. (2020)^S94^ | No outcomes of interest reported. The main outcome was a Health-Related quality of life survey. |
| Cuffari et al. (2021)^S95^ | Conference abstract. |
| Hussain et al. (2021)^S96^ | This study was not randomized and all participants received Lubiprostone. |
| Lee et al. (2021)^S97^ | Conference abstract of a published RCT. The full text version of this study has been published and included in our review (Lee 2022^S98^). |
| Nasri et al. (2021)^S99^ | Participants were randomized to receive either PEG or LaxaPlus Barij. LaxaPlus is a traditional Iranian medicine consisting of various plant extracts. This is not a treatment of interest and therefore the study was excluded. |
| Saneian et al. (2021)^S100^ | In this study, PEG was compared to Golghand which is a herbal-based laxative. This is not a treatment of interest and therefore the study was excluded. |
| Tierney et al. (2022)^S101^ | The definition of functional constipation used in this trial was too dissimilar to the Rome (II, III or IV) criteria to be included in the review. Many of the participants characterized as having constipation in the study would not have constipation according to the Rome criteria given that more that 50% of the participants had 5 or more BMs/week. |

BM, bowel movement; PEG, polyethylene glycol; RCT, randomised controlled trial.

## **Supplementary Table S7** Overview of adverse events as reported in the RCTs in the present review.

| **Author, year (Ref #)** | **Adverse effects examined/reported** | **Treatment groups** | | | | **Additional comments and supporting ‘quotes’, if applicable** |
| --- | --- | --- | --- | --- | --- | --- |
| Voskuijl et al. (2004)^S39^ |  | **PEG** | **Lactulose** |  | *‘During the eight week study period there were no serious or significant adverse events recorded. Figure 2 shows that significantly more adverse events were reported by patients using lactulose compared with patients on PEG.’*  Data were presented in graphical form and were extracted from Figure 2. | |
|  | Abdominal pain  Bad taste  Pain at defecation  Straining at defecation  Bloating  Diarrhea  Flatulence  Nausea  Hard stool consistency  Vomiting | 16  15  6  16  40  15  20  6  14  - | 26  5  22  26  9  3  27  9  18  4 |  |  |  |
| Banaszkewicz et al. (2005)^S102^ |  | **Lactulose + Lactobacillus GG** | **Lactulose** |  | - | |
|  | Abdominal pain  Vomiting  Headache | 3  1  - | 5  -  1 |  |  |  |
| Dupont et al. (2005)^S103^ |  | **PEG** | **Lactulose** |  | *‘Only 6 treatment related adverse events, all nonserious, occurred. These were diarrhea (5 episodes in 2 children in both treatment groups) and anorexia (1 child in the lactulose group).’* | |
|  | Diarrhea  Anorexia | **2**  **-** | **2**  **1** |  |  |  |
| Urganci et al. (2005)^S104^ |  | **Mineral oil** | **Lactulose** |  | *‘None of the patients stopped treatment because of adverse effects.’*  *‘Adverse events included vomiting, bloating and increased flatulence, and abdominal cramping. Bloating and increased flatulence were seen mostly in the higher doses. However, liquid paraffin was better tolerated and compliance was higher compared with the other laxative.’* -number of events of bloating, flatulence and vomiting not reported as a total or broken down by group. | |
|  | Abdominal distention + cramping  Watery stool  Bloating  Flatulence  Vomiting | **-**  **2**  **?**  **?**  **?** | **3**  **-**  **?**  **?**  **?** |  |  |  |
| Loening-Baucke et al. (2006)^S105^ |  | **PEG** | **MoM** |  | *‘One child was allergic to PEG. No other significant clinical adverse effects were reported with either PEG or MOM except for transient diarrhea, which disappeared with dose reduction.’* -number of patients with diarrhea was not reported as a total or broken down by group. | |
|  | Diarrhea  Abdominal pain  Flatulence  Abdominal distention | **?**  **-**  **-**  **-** | **?**  **-**  **-**  **-** |  |  |  |
| Bu et al. (2007)^S106^ |  | **Lactobacillus Rhamnosus** | **Magnesium Oxide** | **Placebo** | ‘…*two patients suffered from acute gastroenteritis (and discontinued treatment)*’ – unclear from which group;  *‘Only one patient in the MgO group suffered from mild diarrhea’* | |
|  | Acute gastroenteritis  Diarrhea | ?  - | ?  1 | ?  - |  |  |
| Farahmand et al. (2007)^S107^ |  | **Mineral oil** | **Lactulose** |  | Side effects reported are from week 4-12 week of intervention.  Percentages of patients who experienced adverse events were reported (in graphical form – Figure 1), therefore the percentages are estimated from the graph and may differ from the true value. | |
|  | Abdominal pain  Bad taste  Pain during defecation  Bloating  Diarhhea  Anal oil leakage  Flatulence  Nausea  Hard stool  Vomiting | 50%  40%  50%  20%  30%  40%  20%  5%  6%  - | 10%  15%  10%  10%  10%  20%  10%  10%  20%  - |  |  |  |
| Thomson et al. (2007)^S20^ |  | **PEG** | **Placebo** |  | *‘*Twenty children (41%) on PEG+E and 22 children (45%) on placebo experienced 41 events and 45 events, respectively, that were judged by the investigator to be at least possibly related to the study treatment.’ | |
|  | Abdominal pain | **39** | **41** |  |  |  |
| Hannah et al. (2008)^S108^ |  | **Synbiotics** | **Placebo** |  | *‘The side effects that frequently occurred was bloating and the difference was not significant (p=0.27)*’ – number of such events broken down by group not specified. | |
|  | Abdominal pain  Bloating  Diarrhea | ? | ? |  |  |  |
| Nurko et al. (2008)^S109^ |  | **PEG** | **Placebo** |  | ‘The frequency of adverse events was similar among the different treatment groups: 14 patients (58.3%) in the placebo group, 9 (34.6%) in 0.2 g/kg, 16 (59.3%) in 0.4 g/kg, and 17 (65.4%) in 0.8 g/kg.’ -number of patients with diarrhea was not reported as a total or broken down by group. | |
|  | Abdominal pain  Fever + headache  Flatulence  Abdominal pain  Nausea  Diarrhea | **?**  **?**  **?**  **?**  **?**  **?** | **?**  **?**  **?**  **?**  **?**  **?** |  |  |  |
| Karami et al. (2009)^S110^ |  | **PEG** | **Mineral oil** |  | Adverse events not reported | |
|  | ? | **?** | **?** |  |  |  |
| Ratanamongkol et al. (2009)^S111^ |  | **PEG** | **MoM** |  | *‘No patient was withdrawn from the study due to adverse effects.’*  *‘Overall adverse effects in both groups were not significantly*  *different (p=0.245).’* | |
|  | Diarrhea  Abdominal pain  Bloating  Nausea/Vomiting | **2**  **9**  **13**  **4** | **12**  **14**  **13**  **9** |  |  |  |
| Coccorullo et al. (2010)^S112^ |  | **Lactobacillus reuteri** | **Placebo** |  | - | |
|  | Bloating  Increased flatulence  Vomiting | -  -  - | -  -  - |  |  |  |
| Khodadad et al. (2010)^S11^ |  | **Synbiotics + Placebo** | **Synbiotics + Mineral oil** | **Mineral oil + Placebo** | - | |
|  | Diarrhea  Seepage  Vomiting | -  -  - | 21  - | -  18  - |  |  |
| Guerra et al. (2011)^S113^ |  | **Probiotic mixture** | **Placebo** |  | ‘*Resolved spontaneously’*; These data were obtained from the 1^st^ authors’ Master thesis (available at <http://www.bibliotecadigital.ufmg.br/dspace/handle/1843/BUOS-8JFMKK>, in Portuguese) where this RCT was described in more detail) and contradicts somewhat the statement in the published report ^S113^: *‘there was no adverse effect due to the intervention in the present study.’* | |
|  | Acute diarrhea | 3 | 1 |  |  |  |
| Gomes et al. (2011)^S114^ |  | **PEG** | **MoM** |  | Adverse events were extracted from the 2-month timepoint. The 4- and 6-month timepoints had less adverse effects than the 2-month timepoint. | |
|  | Abdominal pain  Straining  Fecal incontinence | **8**  **2**  **4** | **8**  **5**  **3** |  |  |  |
| Rafati et al. (2011)^S115^ |  | **PEG** | **Mineral oil** |  | *‘All of adverse reactions (nausea, vomiting, flatulence, abdominal pain and dehydration), other than diarrhea, occurred more frequently in patients using liquid paraffin compared with PEG 3350 (p<0.05) (Table 3).’* | |
|  | Nausea Vomiting Diarrhea Flatulence Abdominal pain Dehydration Withdraw for adverse drug reactions Withdraw for no efficacy | **?**  **?**  **?**  **?**  **?**  **?**  **?**  **?** | **?**  **?**  **?**  **?**  **?**  **?**  **?**  **?** |  |  | |
| Tabbers et al. (2011)^S19^ |  | **Probiotic mixture** | **Placebo** |  | - | |
|  | Bad taste  Diarrhea  Gastroenteritis  Nausea/vomiting  Candida infection in anorectal region | -  -  1  3  - | -  -  3  2  1 |  |  |  |
| Saneian et al. (2012)^S116^ |  | **PEG** | **MoM** | **Lactulose** | *‘The statistical studies showed that incidence of abdominal pain in PEG significantly was lower than other groups (P = 0.001).’* | |
|  | Abdominal pain  Nausea + Vomiting  Bloating  Diarrhea  Stomach irritation | **2**  **0**  **2**  **0**  **1** | **17**  **0**  **1**  **5**  **1** | 14  1  17  1  2 |  |  |
| Wang et al. (2012)^S117^ |  | **PEG** | **Lactulose** |  | - | |
|  | Abdominal pain  Diarrhea  Blood in stool | **1**  **1**  **-** | **-**  **-**  **-** |  |  |  |
| Olgaç et al. (2013)^S118^ |  | **Lactobacillus reuteri** | **Lactulose** |  | - | |
|  | Abdominal pain  Flatulence  Rectal bleeding | **?**  **?**  **?** | **?**  **?**  **?** |  |  |  |
| Mugie et al. (2014)^S16^ |  | **Prucalopride** | **Placebo** |  | *‘During the double-blind period, treatment-emergent adverse events (TEAEs) were reported by 69.8% of patients in the prucalopride group (tablet: 81.8%; solution: 66.7%) and 60.7% of patients in the placebo group (Table 3).’*  *‘Two patients (1 in each treatment group) permanently discontinued treatment because of a TEAE.’* | |
|  | Headache  Pyrexia  Abdominal pain  Vomiting  Nausea  Viral infection  Cough  Diarrhea  Nasopharyngitis  Pharyngitis  Bronchitis  Upper respiratory tract infection  Abdominal pain  Constipation  Vomiting  Diarrhea  Nausea  Appendicitis  Pneumonia  Dizziness  Syncope  Anxiety  Viral infection  Contusion  Proctalgia  Anorectal discomfort | **17**  **15**  **14**  **15**  **10**  **6**  **6**  **6**  **3**  **3**  **2**  **2**  **2**  **1**  **1**  **1**  **1**  **1**  **1**  **1**  **1**  **1**  **1**  **0**  **0**  **0**  **0** | **9**  **3**  **13**  **5**  **6**  **5**  **2**  **6**  **2**  **6**  **7**  **5**  **3**  **2**  **1**  **1**  **0**  **0**  **0**  **0**  **0**  **0**  **0**  **0**  **0**  **0**  **0**  **1** |  |  |  |
| Sadeghzadeh et al. (2014)^S119^ |  | **Lactulose + Probiotic mixture** | **Lactulose** |  | - | |
|  | ? | - | - |  |  |  |
| Treepongkaruna et al. (2014)^S21^ |  | **PEG** | **lactulose** |  | *‘Over the course of the study, 55 treatment-emergent adverse events (TEAEs) were reported in 26 children in the lactulose group (59.1%) and 80 TEAEs reported in 27 children in the PEG 4000 group (61.4%).’* | |
|  | Anal dilation  Upper respiratory tract infections  Anal fissure  Faecaloma  Hard faeces  Anal skin tags  Rhinorrhoea  Vomiting | **14**  **11**  **6**  **10**  **4**  **2**  **1**  **0** | **11**  **11**  **10**  **6**  **3**  **5**  **3**  **3** |  |  |  |
| Ala et al. (2015)^S120^ |  | **PEG** | **Lactulose** |  | *‘Adverse effects such as abdominal pain, diarrhea, and flatulence were seen in 15% of patients treated with lactu­lose in group II, but not seen with PEG.’* – number of adverse events was not reported as a total or broken down by type of event. | |
|  | *Abdominal pain Diarrhea*  *Flatulence* | **-**  **-**  **-** | **?**  **?**  **?** |  |  |  |
| Hashemi et al. (2015)^S7^ |  | **PEG** | **Probiotic mixture** | **PEG + Probiotic mixture** | - | |
|  | Headache  Nausea  Vomiting | -  -  - | 1  -  - | -  -  - |  |  |
| Abediny et al. (2016)^S1^ | ? | **PEG** | **Synbiotics** |  | Mentioned in abstract and methods section as outcome of interested but no data provided in the results or discussion sections. | |
|  |  | ? | ? |  |  |  |
| Wojtyniak et al. (2017)^S81^ |  | **Lactobacillus rhamnosus** | **Placebo** |  | - | |
|  | Stool odor (change in)  Abdominal pain and flatulence  Loss of appetite | -  -  - | 1  1  1 |  |  |  |
| Mahdavi et al. (2017)^S14^ |  | **PEG + Synbiotics** | **PEG** |  | - | |
|  | Abdominal pain  Diarrhea  Nausea  Skin symptoms  Vomiting | 2  2  1  -  - | 2  1  -  -  - |  |  |  |
| Russo et al. (2017)^S69^ |  | **PEG + Probiotic mixture** | **PEG** |  | 3 children dropped-out of the RCT due to the bad taste experienced;  *‘No significant clinical adverse effects were reported with either PEG or PEG+PM except for transient diarrhea, which disappeared with dose reduction.’* – exact numbers within each group not reported.  *‘There were no complaints of abdominal distention, increased flatus, or new onset of abdominal pain’…’There were no new abnormal physical findings on examination.’* | |
|  | Bad taste  Diarrhea  Flatulence, meteorism, nausea or vomiting, abdominal distention and pain | 1  ?  - | 2  ?  - |  |  |  |
| Baştürk et al. (2017)^S121^ |  | **Synbiotics** | **Placebo** |  | - | |
|  | Vomiting  Diarrhea | -  - | -  - |  |  |  |
| Torabi et al. (2017)^S122^ |  | **PEG** | **Mineral oil** |  | *‘The frequency of adverse effects was similar and didn’t differ significantly between the two groups’* | |
|  | Abdominal pain  Fecal incontinence | **17**  **-** | 15  2 |  |  |  |
| Cao et al. (2018)^S123^ |  | **Lactulose** | **Placebo** |  | *‘no significant differences regarding all adverse events were detected between 2 groups.’* | |
|  | Anal dilation  Upper respiratory tract infections  Faecaloma  Anal fissure  Hard faeces  Rhinorrhoea | 11  8  9  7  4  1 | 8  6  6  5  2  2 |  |  |  |
| Wegner et al. (2018)^S71^ |  | **PEG + Lactobacillus reuteri** | **PEG** |  | *‘The reported adverse events did not cause withdrawal from the trial’* | |
|  | Abdominal pain | 2 | - |  |  |  |
| Jose et al. (2018)^S124^ |  | **Lactulose + Probiotic mixture** | **Lactulose** |  | - | |
|  | ? | - | - |  |  |  |
| Jadrešin et al. (2018)^S8^ |  | **Lactulose + Lactobacillus reuteri** | **Lactulose** |  | - | |
|  | ? | - | - |  |  |  |
| Modin et al. (2018)^S15^ |  | **PEG** | **Placebo** |  | *‘Significantly more adverse events related to the gastrointestinal tract were observed in children receiving placebo, compared with children receiving PEG (69% [22] vs 28%, [7]; P¼0.002).’* | |
|  | Abdominal pain  Bloating  Nausea/vomiting | 6  1  - | 22  0  - |  |  |  |
| Cassettari et al. (2019)^S125^ |  | **PEG** | **Sodium Picosulfate** |  | *‘Green banana biomass alone and associ-ated with laxatives was well tolerated, and adverse effects were not reported.’* | |
|  | Pain  Nausea  Vomiting  Diarrhea  Flatulence | -  -  -  -  - | -  -  -  -  - |  |  |  |
| Jarzebicka et al. (2019)^S9^ |  | **PEG** | **Lactulose** |  |  | |
|  | Abdominal pain  Diarrhea  Nausea/vomiting  Bloating/Flatulence  Irritation of the anal area | 10  6  1  20  10 | 17  3  1  35  9 |  | *‘Significantly more side effects were observed in the lactulose group at both weeks 4 (27 vs 38, respectively, P¼0.04) and also at week 12 (15 vs 23, respectively, P¼0.02).’*  Numbers shown here are from the week 4 follow-up because there were more adverse events at this timepoint. | |
|  | Abdominal pain | 4 | 3 |  |  | |
| Kubota et al. (2020)^S12^ |  | **Lactobacillus reuteri + Placebo** | **Lactobacillus reuteri + MgO** | **MgO + Placebo** | *‘No adverse event related to any treatment was observed in this study.’* | |
|  |  | - | - | - |  |  |
| Tjokronegoro et al. (2020)^S126^ |  | **Probiotic mixture** | **Placebo** |  | *-* | |
|  | Abdominal pain  Diarrhea | 2  - | 4  2 |  |  |  |
| Dheivamani et al. (2021)^S127^ |  | **PEG 3350** | **Lactulose** |  |  | |
|  | ? | - | - |  |  |  |
| Worona-Dibner et al. (2023)^S128^ |  | **PEG** | **MoM** |  | ‘The main adverse events detected were gases, cramps, FI, and diarrhea, all of which improved upon reducing the dose of the laxative, coinciding with data reported by other authors.’  *‘The parents were instructed to keep a diary, during the entire study, registering:*  *…Adverse events/day (yes/no): cramps, gases, abdominal pain, diarrhea, non-retentionist FI (presence of stool on underwear and liquid stools in the toilet), vomiting, dehydration, and others, with their specification.’* | |
|  | Gases  Colic  Fecal incontinence  Diarrhea  Vomiting  Nausea  Other  Headache  Bloating  Pain during defecation  Belching  Thirst  Dizziness  General malaise  Cramping  Lower limb pain  Hair loss  Exanthem | 40  33  20  17  13  14  8  4  2  0  1  1  1  0  1  0  1  1 | 37  30  25  24  13  14  10  5  5  5  1  1  1  1  0  1  0  0 |  |  |  |
| Benninga et al. (2022)^S3^ |  | **Lubiprostone** | **Placebo** |  | *‘nausea and vomiting were common AEs that led to lubiprostone discontinuation in study 1.’*  *‘Because lubiprostone dosing in the pediatric studies was primarily based on weight, it is important to examine the potential impact of weight on AEs. In general, vomiting was more common in children weighing <50 kg, while nausea and headache were more common in children weighing ≥50 kg.’* | |
|  | Headache  Nausea  Vomiting  Abdominal pain  Nasopharyngitis  Diarrhea | 34  57  45  42  ?  ? | 10  14  12  23  ?  ? |  |  |  |
| Foroughi et al. (2022)^S129^ |  | **PEG** | **PEG + Probiotics** |  | ? | |
|  | ? |  |  |  |  |  |
| Gan et al. (2022)^S5^ |  | **Probiotic mixture** | **Placebo** |  |  | |
|  | ? |  |  |  |  |  |
| Lee et al. (2022)^S98^ |  | **Saccharomyces boulardii** | **Lactulose** |  | *‘Abdominal pain was the most common adverse event (20.9%, 11.3%, and 1.8% at weeks 2, 6, and 12, respectively), followed by diarrhea (6.3% and 4.7% at weeks 2 and 6, respectively), abdominal distension (4.4% at week 2), and vomiting (1.3% at week 2). The frequency of adverse effects decreased from week 2 to week 12; no vomiting or distension was observed at week 6; and no vomiting, distension, or diarrhea was observed at week 12. There were no intergroup differences in the adverse events.’* | |
|  | Abdominal pain  Diarrhea  Abdominal distension  Vomiting | ?  ?  ?  ? | ?  ?  ?  ? |  |  |  |
| Mansour et al. (2022)^S130^ |  | **PEG** | **Lactulose** |  | *‘We reported significantly more side effects in patients who received Lactulose compared with the patients who received PEG.’* | |
|  | Abdominal pain  Bloating  Diarrhea  Anal irritation | 5  -  8  - | 15  6  9  5 |  |  |  |
| Lojantorn et al. (2023)^S13^ |  | **Bacillus clausii** | **Placebo** |  | *‘No serious adverse effects were observed. One patient had urticaria and another one with abdominal pain in the B. clausii group (n=2) and one patient in the placebo group had vomiting.’* | |
|  | Urticaria  Abdominal pain  Vomiting | 1  1  - | -  -  1 |  |  |  |

## **Supplementary Table S8** Comparison between direct and indirect evidence by comparison for the NMA of defecation frequency.

| **Comparison** | **Number of studies providing direct evidence** | **Direct evidence proportion** | **NMA** | **Direct** | **Indirect** | **Difference (Direct and Indirect)** | **z** | **p-value** |
| --- | --- | --- | --- | --- | --- | --- | --- | --- |
| Lactulose:Lactulose+Probiotics | 5 | 0.96 | -0.12 | -0.01 | -2.73 | 2.72 | 1.15 | 0.2512 |
| Lactulose:Magnesium Hydroxide | 1 | 0.31 | -1.59 | -1.51 | -1.62 | 0.11 | 0.08 | 0.9360 |
| Lactulose:Mineral oil | 2 | 0.4 | -2.52 | -4.7 | -1.07 | -3.63 | -3.06 | **0.0022** |
| Lactulose:PEG | 8 | 0.71 | -1.59 | -1.28 | -2.36 | 1.08 | 1.34 | 0.1816 |
| Lactulose:Placebo | 1 | 0.25 | 0.13 | 0.3 | 0.08 | 0.22 | 0.19 | 0.8497 |
| Lactulose:Probiotics | 2 | 0.39 | -0.44 | -0.07 | -0.67 | 0.61 | 0.63 | 0.5287 |
| Lactulose+Probiotics:Probiotics | 1 | 0.3 | -0.32 | 0.88 | -0.83 | 1.71 | 1.24 | 0.2139 |
| Magnesium Hydroxide:PEG | 5 | 0.93 | -0.01 | 0.11 | -1.58 | 1.68 | 0.76 | 0.4498 |
| Magnesium Oxide:Placebo | 1 | 0.47 | 0.95 | 2.15 | -0.13 | 2.28 | 1.55 | 0.1200 |
| Magnesium Oxide:Probiotics | 2 | 0.87 | 0.38 | -0.13 | 3.77 | -3.89 | -1.90 | **0.0577** |
| Magnesium Oxide:Probiotics+Magnesium Oxide | 1 | 0.87 | 0.2 | 0.01 | 1.44 | -1.44 | -0.52 | 0.6058 |
| Mineral oil:Mineral oil+Probiotics | 1 | 0.86 | -0.48 | -0.74 | 1.05 | -1.79 | -0.51 | 0.6088 |
| Mineraloil:PEG | 2 | 0.53 | 0.93 | -0.43 | 2.45 | -2.88 | -2.54 | **0.0112** |
| Mineral oil:Probiotics | 1 | 0.27 | 2.08 | 1.53 | 2.28 | -0.75 | -0.51 | 0.6088 |
| Mineral oil+Probiotics:Probiotics | 1 | 0.83 | 2.56 | 2.27 | 3.94 | -1.67 | -0.51 | 0.6088 |
| PEG:PEG+Probiotics | 5 | 0.95 | 0.02 | -0.08 | 2.02 | -2.1 | -0.95 | 0.3445 |
| PEG:Placebo | 2 | 0.29 | 1.72 | 1.74 | 1.71 | 0.03 | 0.03 | 0.9783 |
| PEG:Probiotics | 1 | 0.22 | 1.15 | 0.08 | 1.46 | -1.38 | -1.18 | 0.2378 |
| PEG+Probiotics:Probiotics | 1 | 0.36 | 1.14 | 0.82 | 1.31 | -0.49 | -0.37 | 0.7093 |
| Probiotics:Placebo | 8 | 0.81 | 0.57 | 0.51 | 0.83 | -0.32 | -0.35 | 0.7261 |
| Probiotics:Probiotics+Magnesium Oxide | 1 | 0.87 | -0.19 | 0 | -1.43 | 1.44 | 0.52 | 0.6058 |

## **Supplementary Table S9** Comparison between direct and indirect evidence by comparison for the NMA of treatment success.

| **Comparison** | **Number of studies providing direct evidence** | **Direct evidence proportion** | **NMA** | **Direct** | **Indirect** | **Ratio of ratios (direct vs indirect)** | **z** | **p-value** |
| --- | --- | --- | --- | --- | --- | --- | --- | --- |
| Lactulose:Lactulose+Probiotics | 3 | 0.95 | 0.92 | 1.03 | 0.11 | 9.19 | 2.40 | **0.0164** |
| Lactulose:Mineral oil | 2 | 0.59 | 0.58 | 0.47 | 0.78 | 0.6 | -1.37 | 0.1711 |
| Lactulose:PEG | 4 | 0.77 | 0.71 | 0.68 | 0.83 | 0.82 | -0.56 | 0.5737 |
| Lactulose:Probiotics | 1 | 0.30 | 0.96 | 2.06 | 0.69 | 2.97 | 2.36 | **0.0181** |
| Lactulose+Probiotics:Probiotics | 1 | 0.47 | 1.04 | 1.82 | 0.63 | 2.9 | 1.99 | 0.0471 |
| Lubiprostone:Placebo | 1 | 1 | 1.29 | 1.29 | . | . | . | . |
| Magnesium Hydroxide:PEG | 3 | 1 | 0.91 | 0.91 | . | . | . | . |
| Magnesium Oxide:Placebo | 1 | 0.14 | 1.49 | 6.5 | 1.18 | 5.51 | 1.59 | 0.1115 |
| Magnesium Oxide:Probiotics | 1 | 0.97 | 1.02 | 0.93 | 26.43 | 0.04 | -1.59 | 0.1115 |
| Mineral oil:Mineral oil+Probiotics | 1 | 0.88 | 1.29 | 1.09 | 4.11 | 0.27 | -1.47 | 0.1408 |
| Mineral oil:PEG | 1 | 0.35 | 1.22 | 1.18 | 1.25 | 0.94 | -0.15 | 0.8777 |
| Mineral oil:Probiotics | 1 | 0.47 | 1.65 | 1.17 | 2.24 | 0.52 | -1.47 | 0.1408 |
| Mineral oil+Probiotics:Probiotics | 1 | 0.85 | 1.28 | 1.07 | 3.72 | 0.29 | -1.47 | 0.1408 |
| PEG:PEG+Lactulose | 1 | 1 | 0.8 | 0.8 | . | . | . | . |
| PEG:PEG+Probiotics | 2 | 1 | 0.91 | 0.91 | . | . | . | . |
| PEG:Placebo | 2 | 0.62 | 1.97 | 1.72 | 2.45 | 0.7 | -0.84 | 0.4 |
| PEG:Sodium Picosulfate | 1 | 1 | 1.33 | 1.33 | . | . | . | . |
| Probiotics:Placebo | 7 | 0.87 | 1.46 | 1.53 | 1.08 | 1.42 | 0.84 | 0.4 |


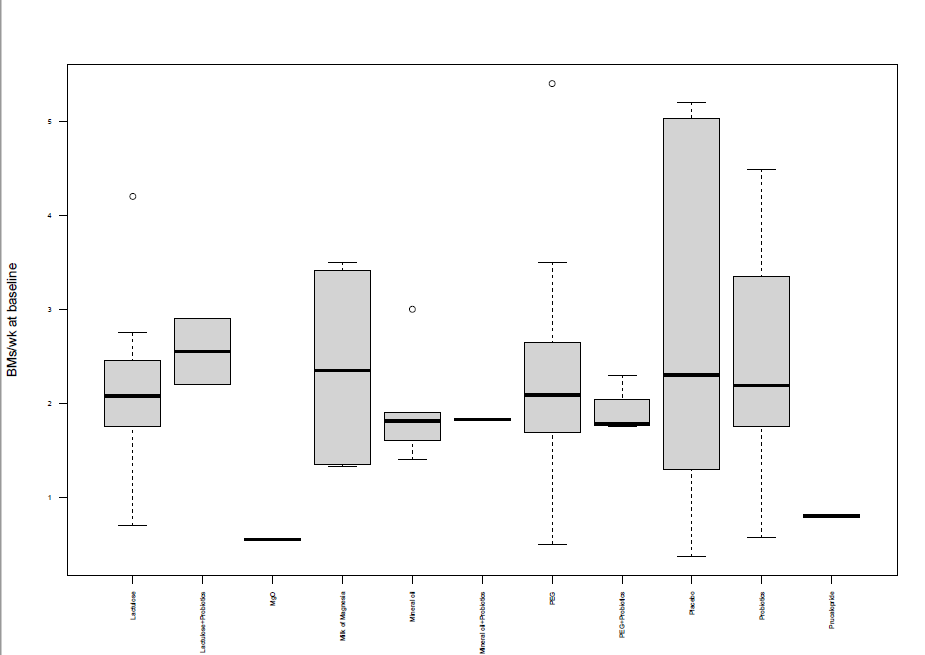
A


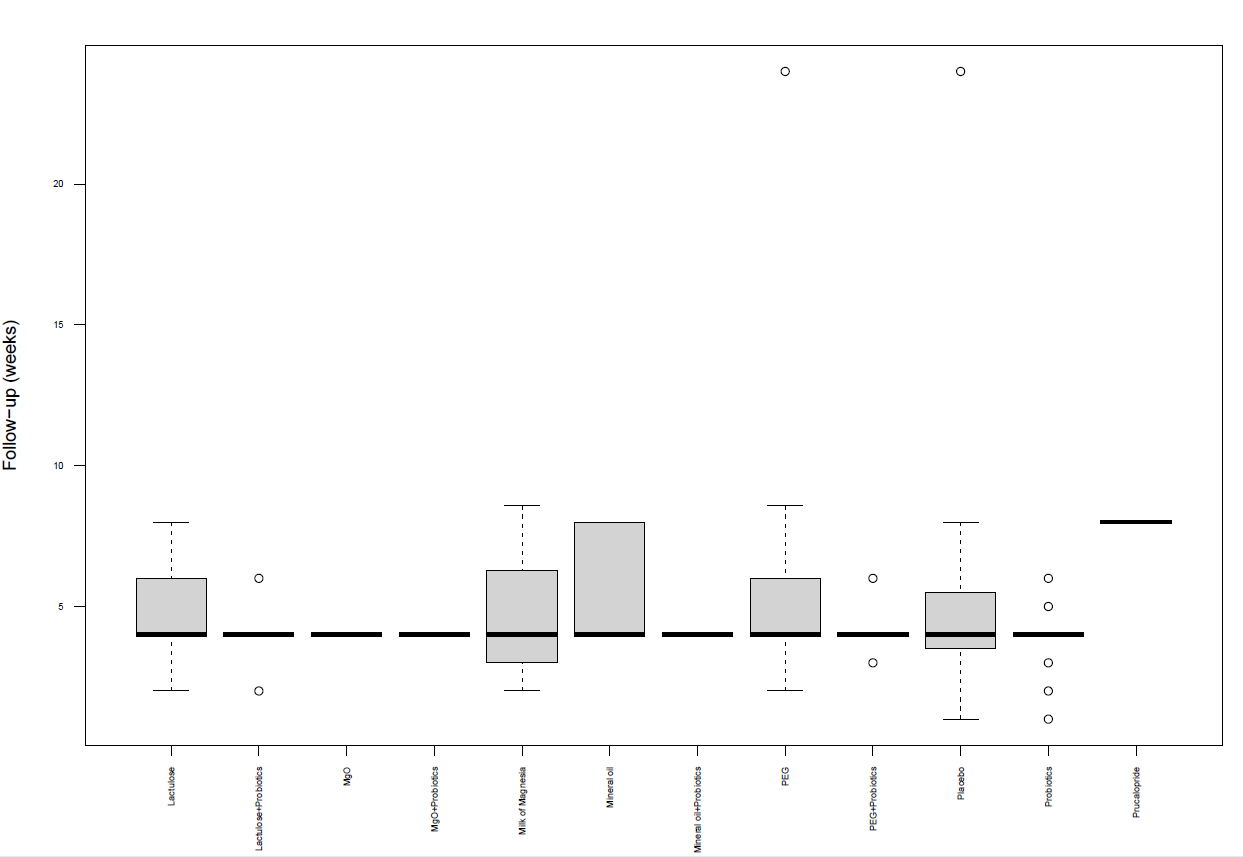
B

## **Supplementary Figure S1** Boxplots comparing A) BMs/wk at baseline, B) duration of follow-up, C) sample size, D) publication year, E) mean age of participants, F) duration of FC and G) Percentage female participants in the RCTs contributing to the analysis of defecation frequency by treatment group.


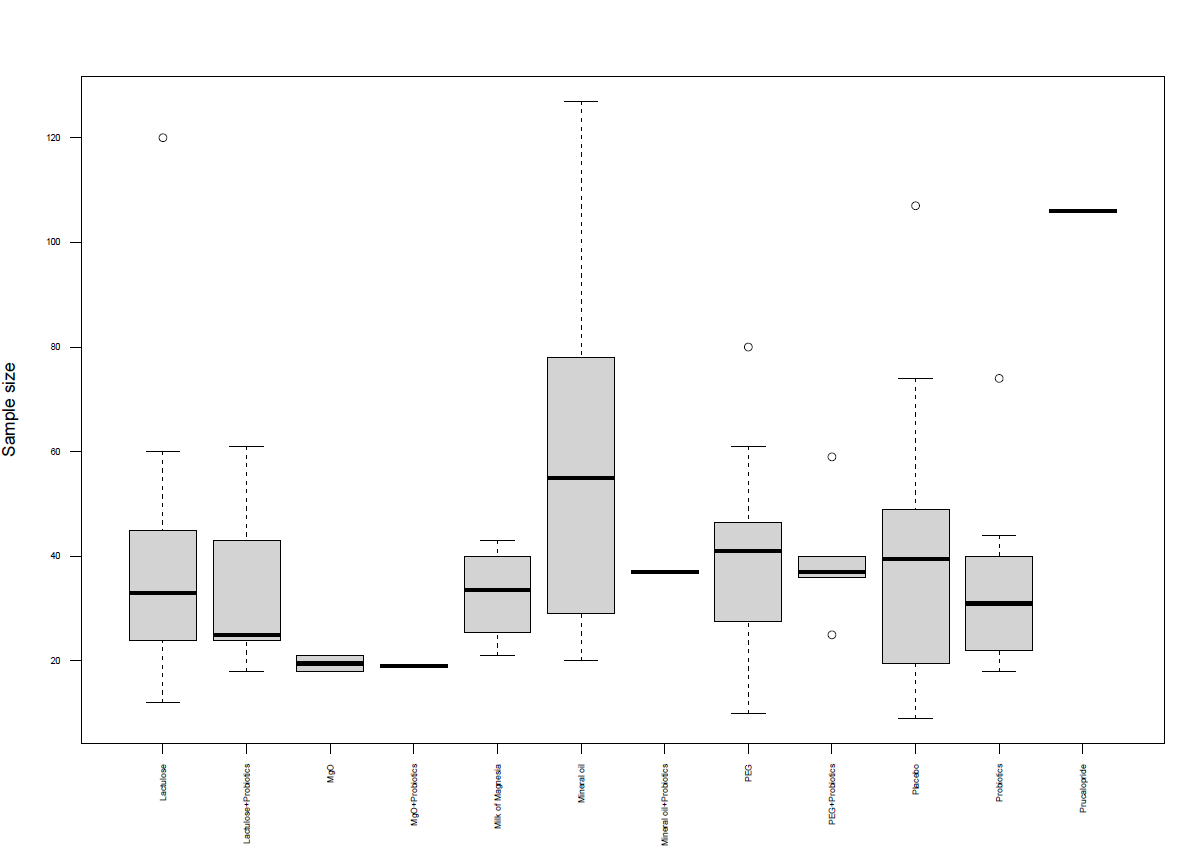
C


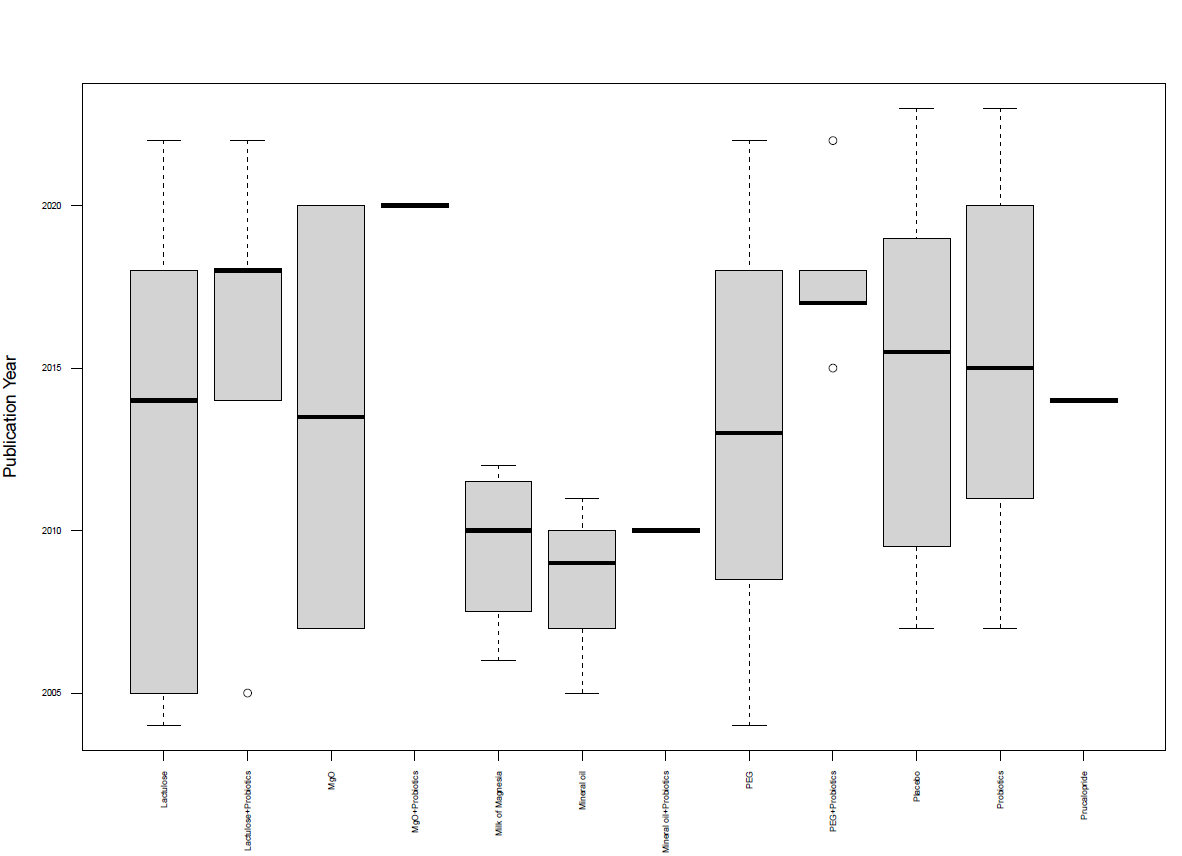
D

Supplementary Figure 1 **Continued**


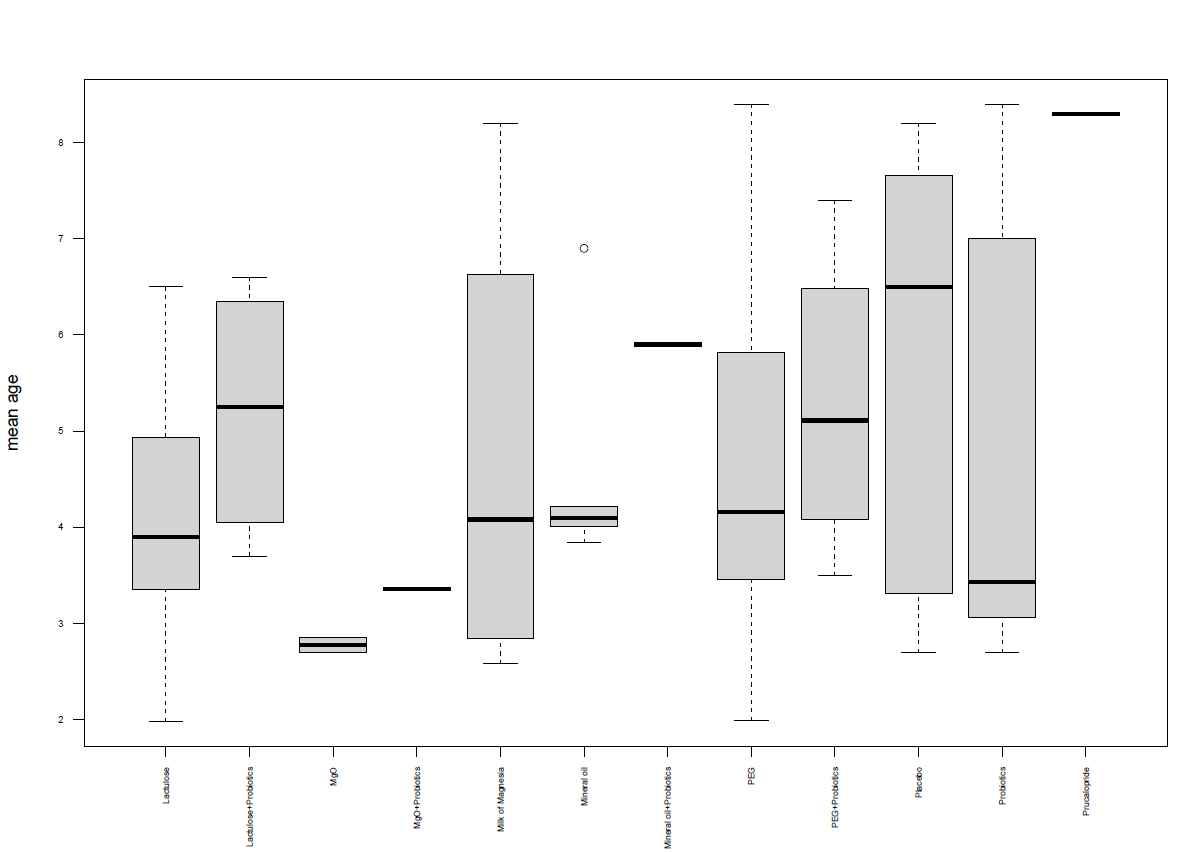
E

F


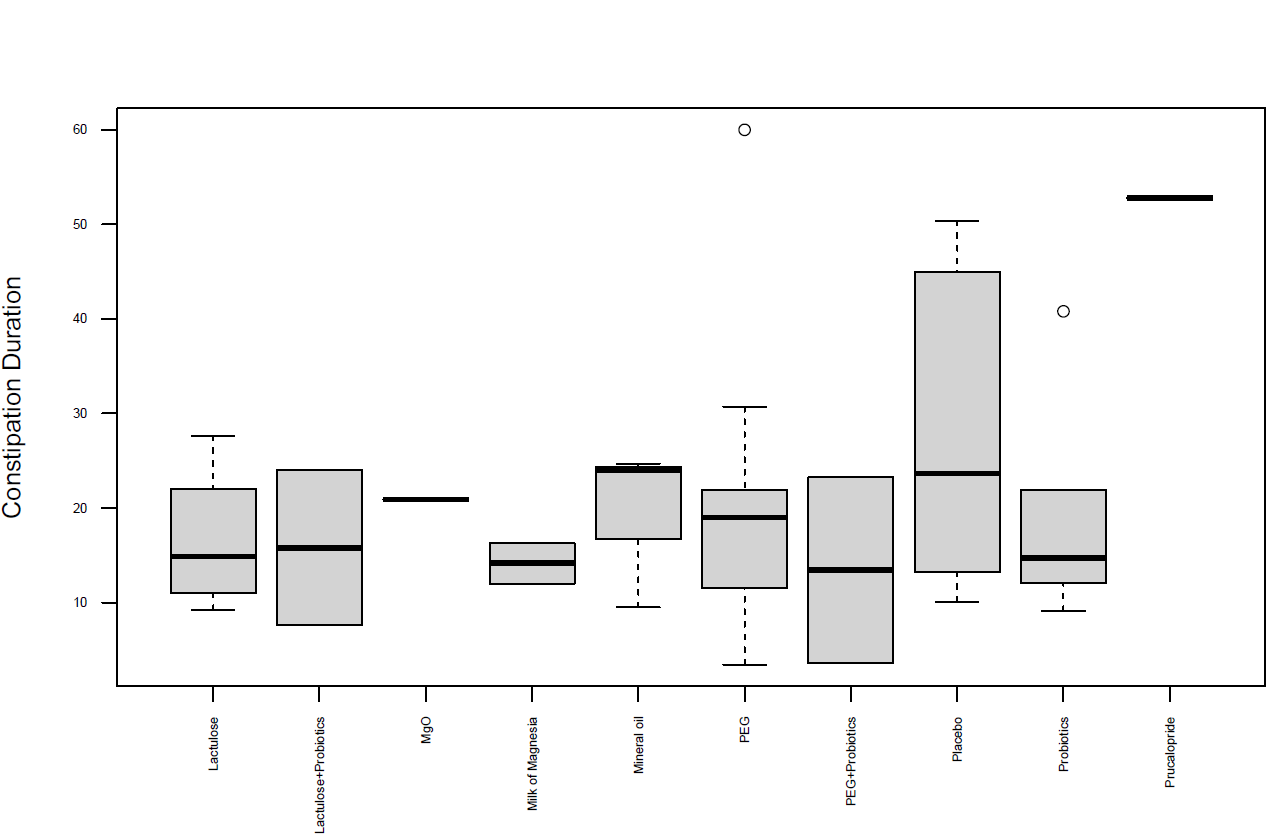


Supplementary Figure 1 **Continued**


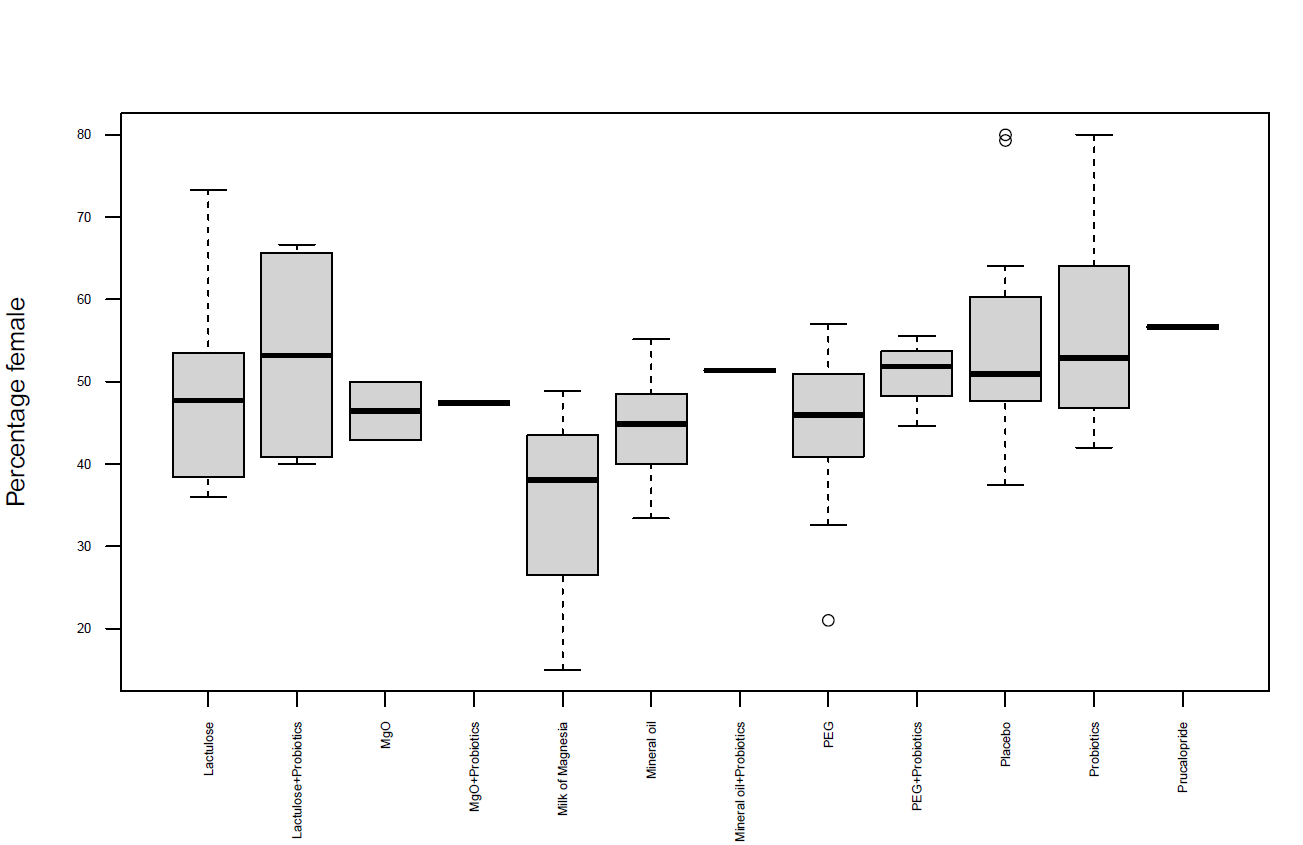
G

Supplementary Figure 1 **Continued**


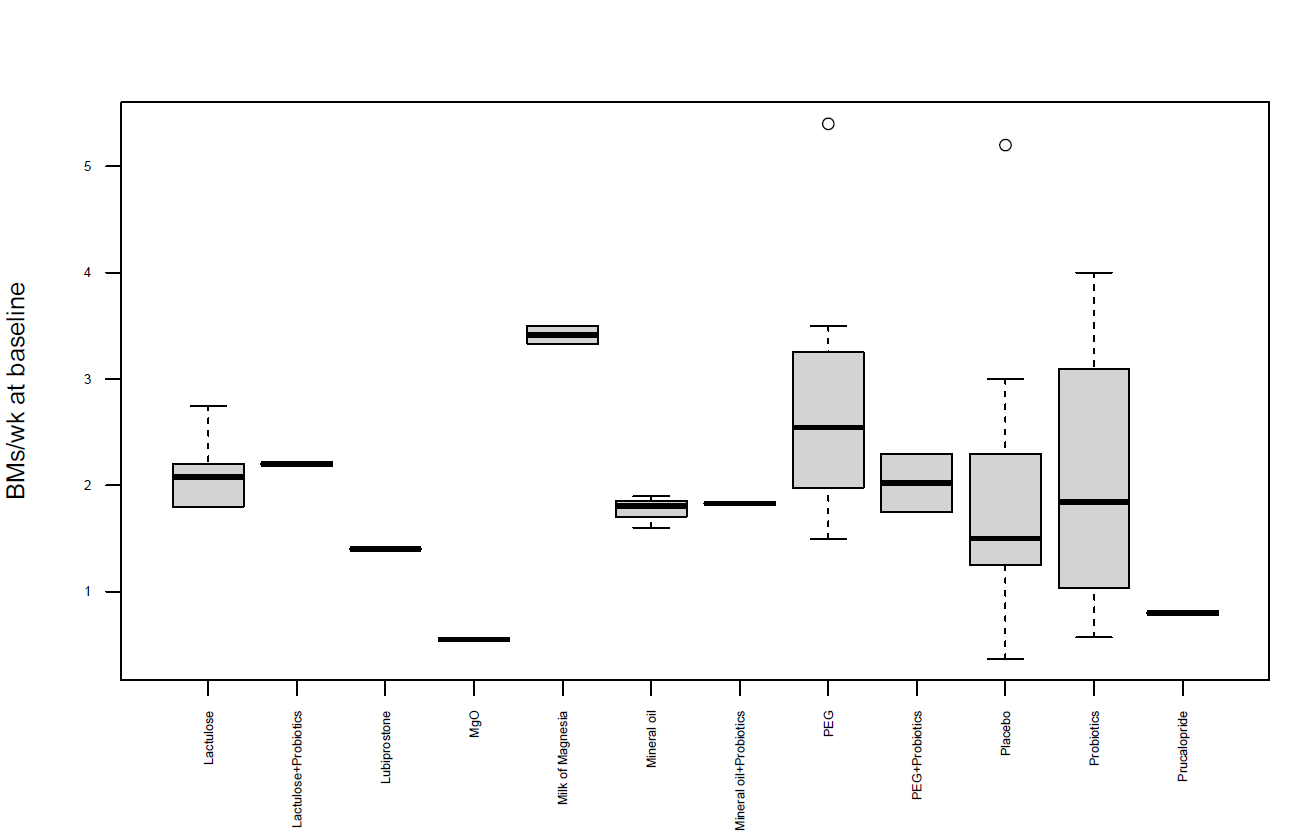
A


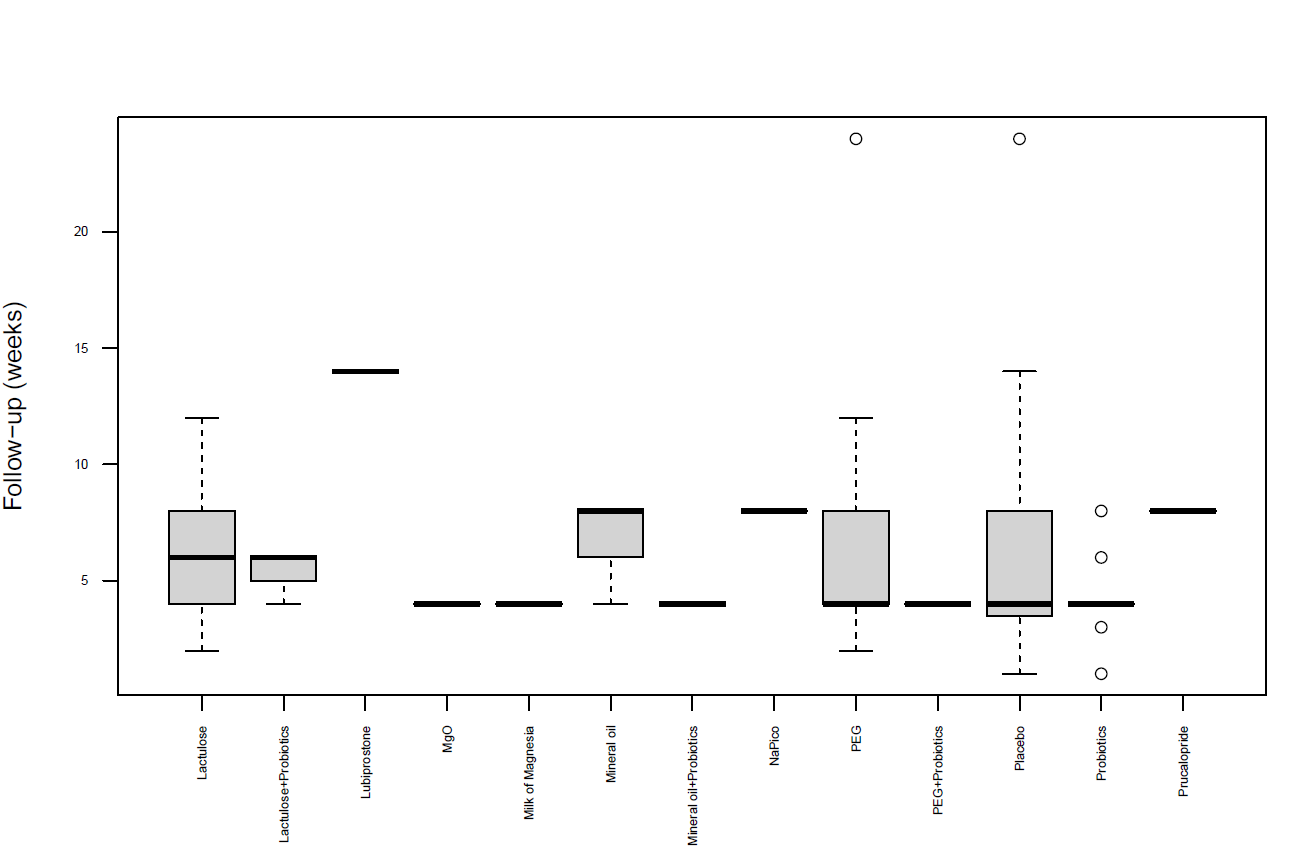
B

## **Supplementary Figure S2** Boxplots comparing A) BMs/wk at baseline, B) duration of follow-up, C) sample size, D) publication year, E) mean age of participants, F) duration of FC and G) Percentage female participants in the RCTs contributing to the analysis of treatment success by treatment group.


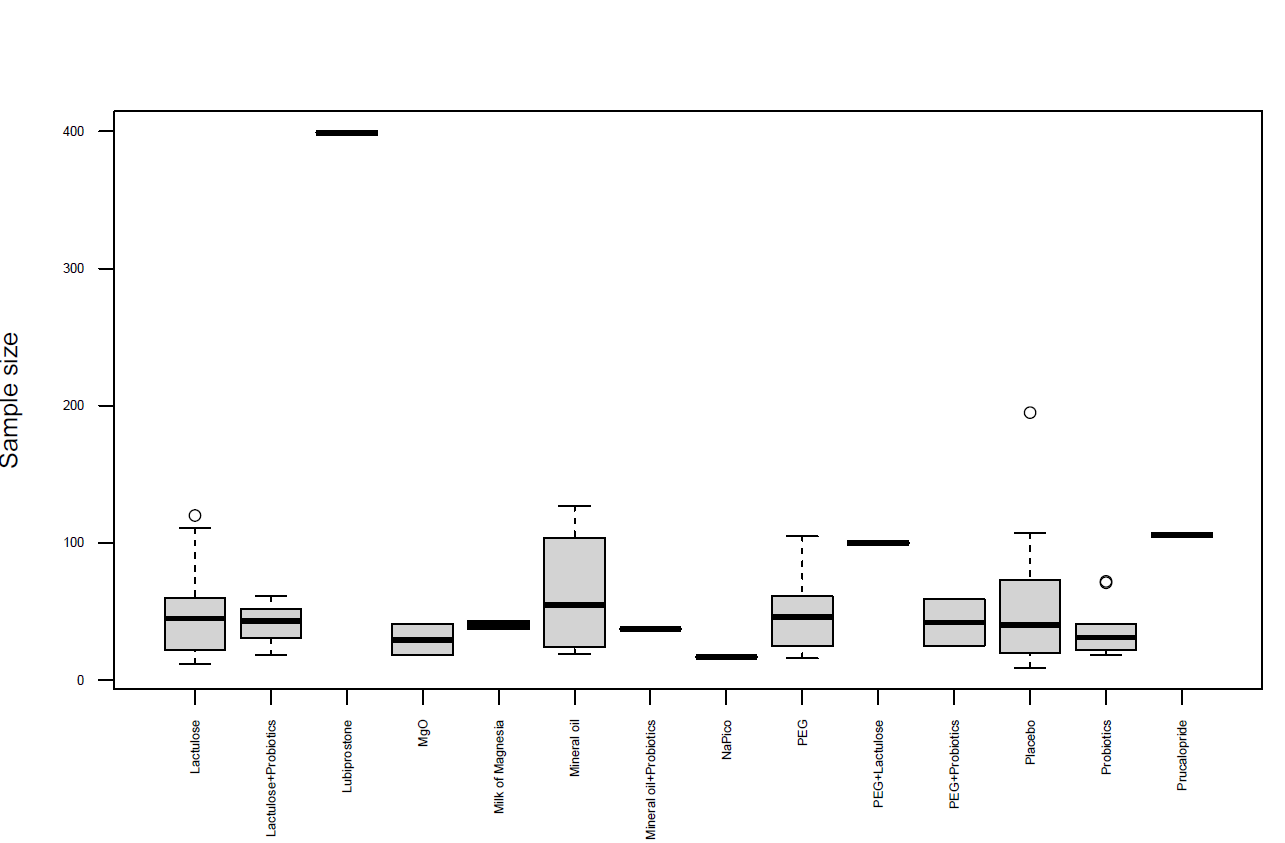
C

D


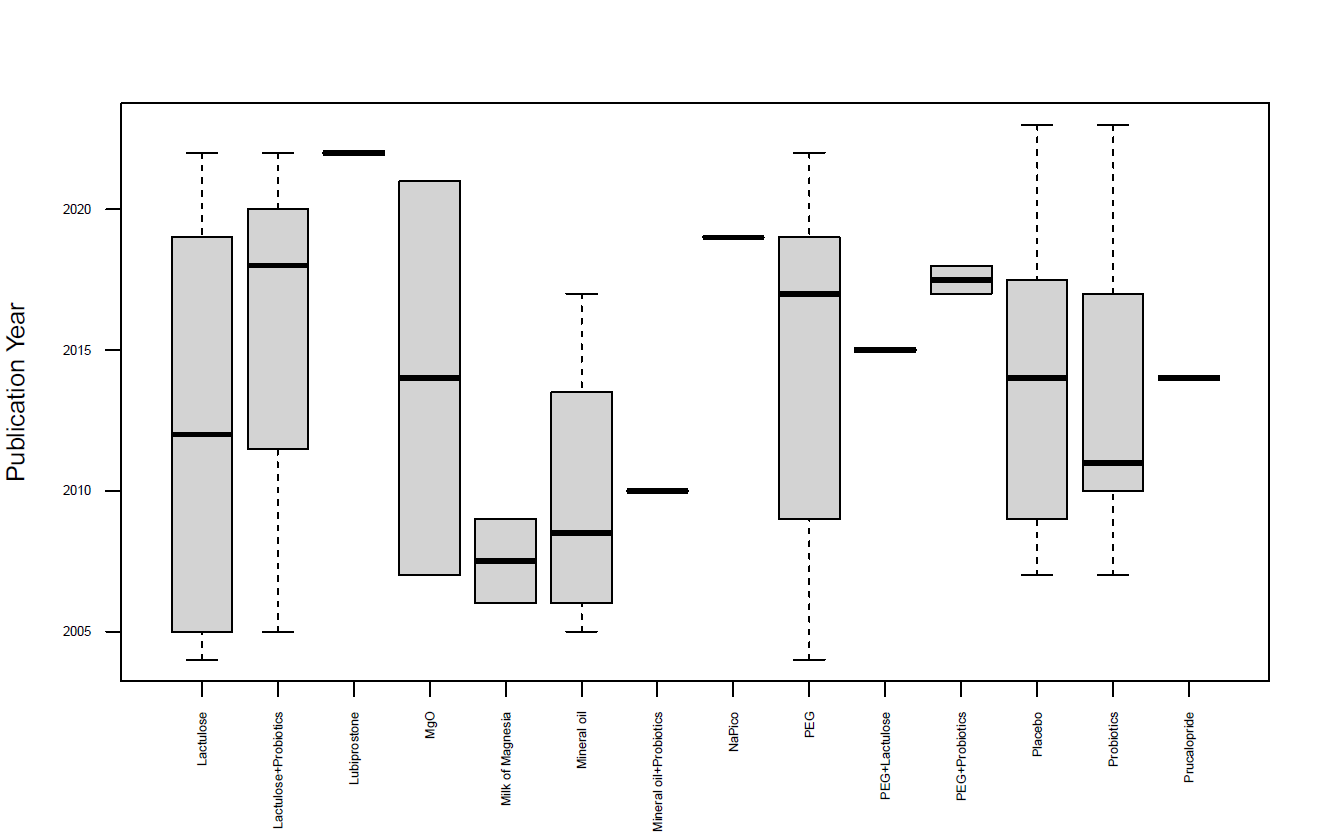


Supplementary Figure 2 **Continued**


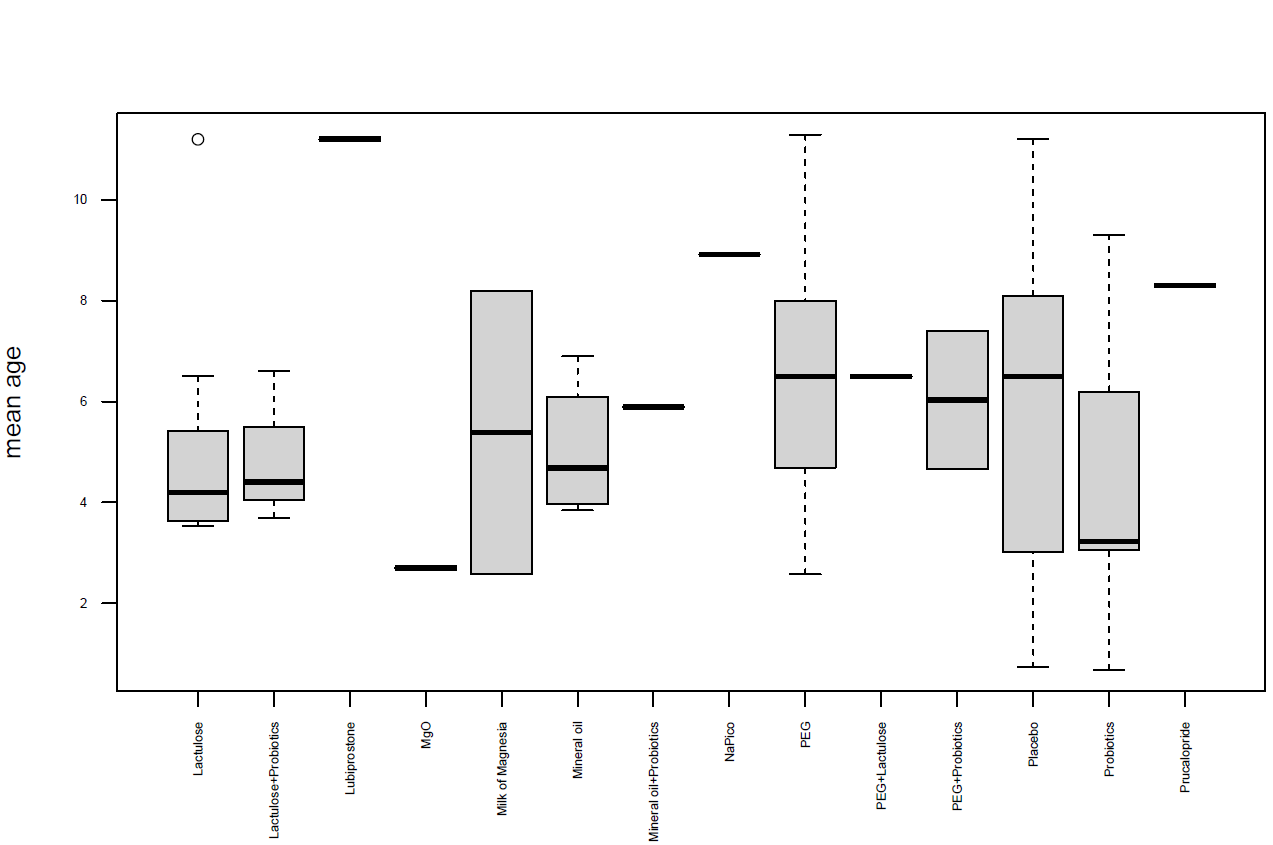
E


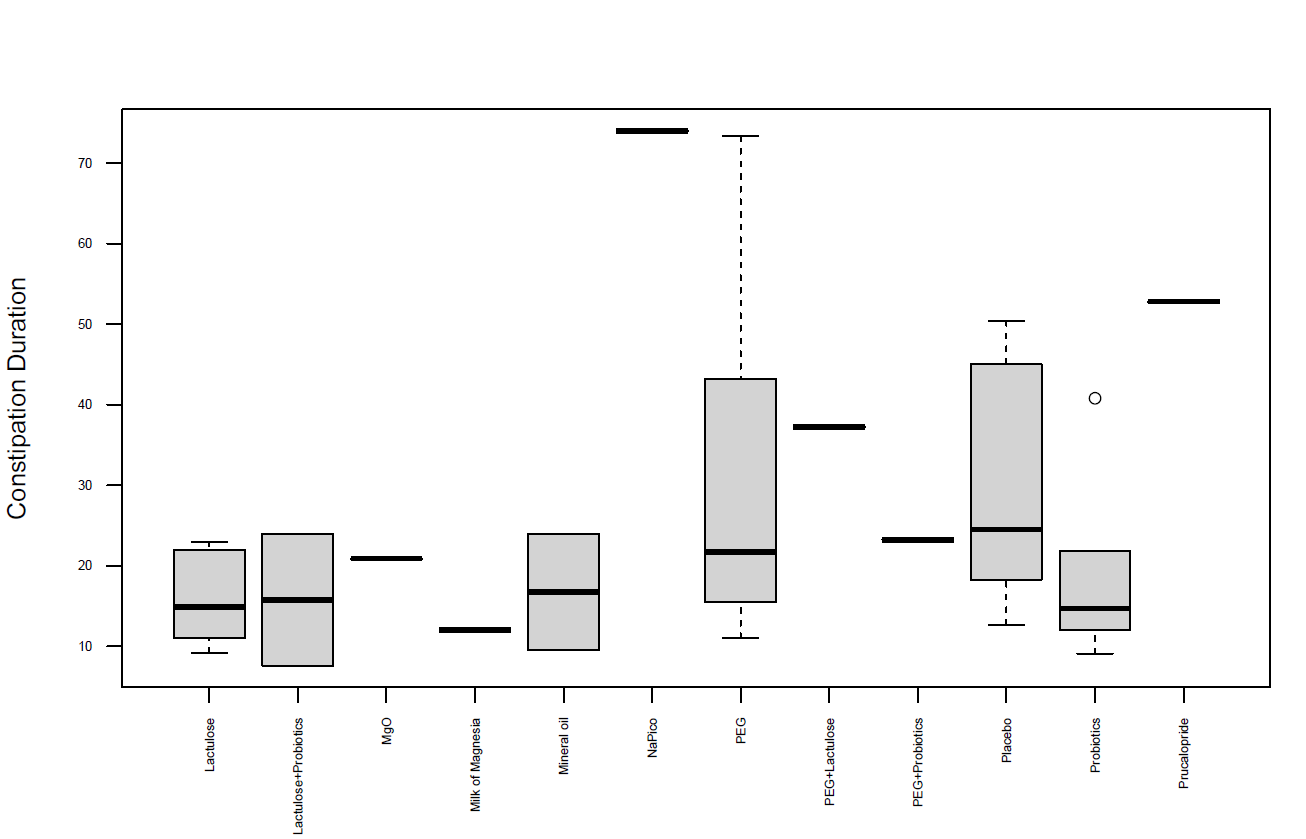
F

Supplementary Figure 2 **Continued**


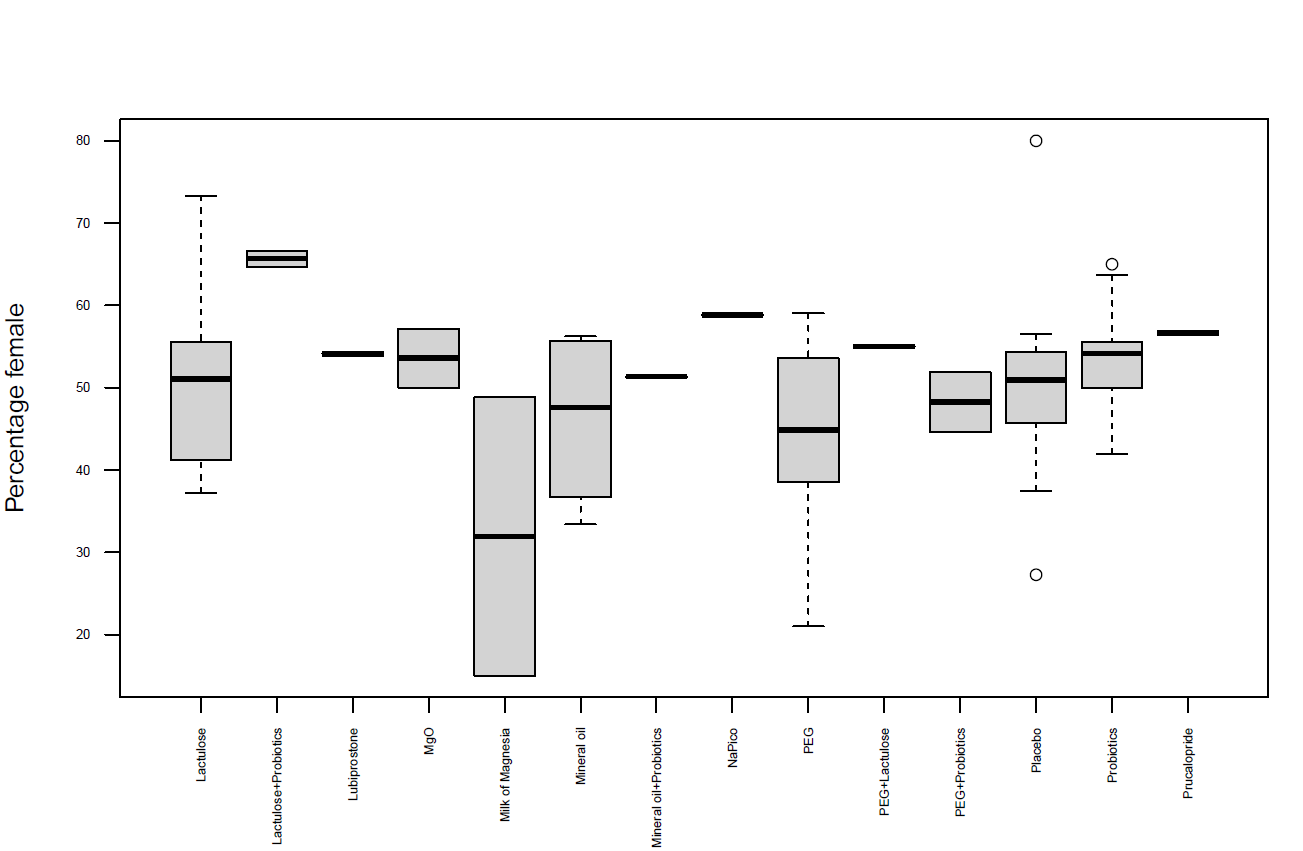
G

Supplementary Figure 2 **Continued**

## **
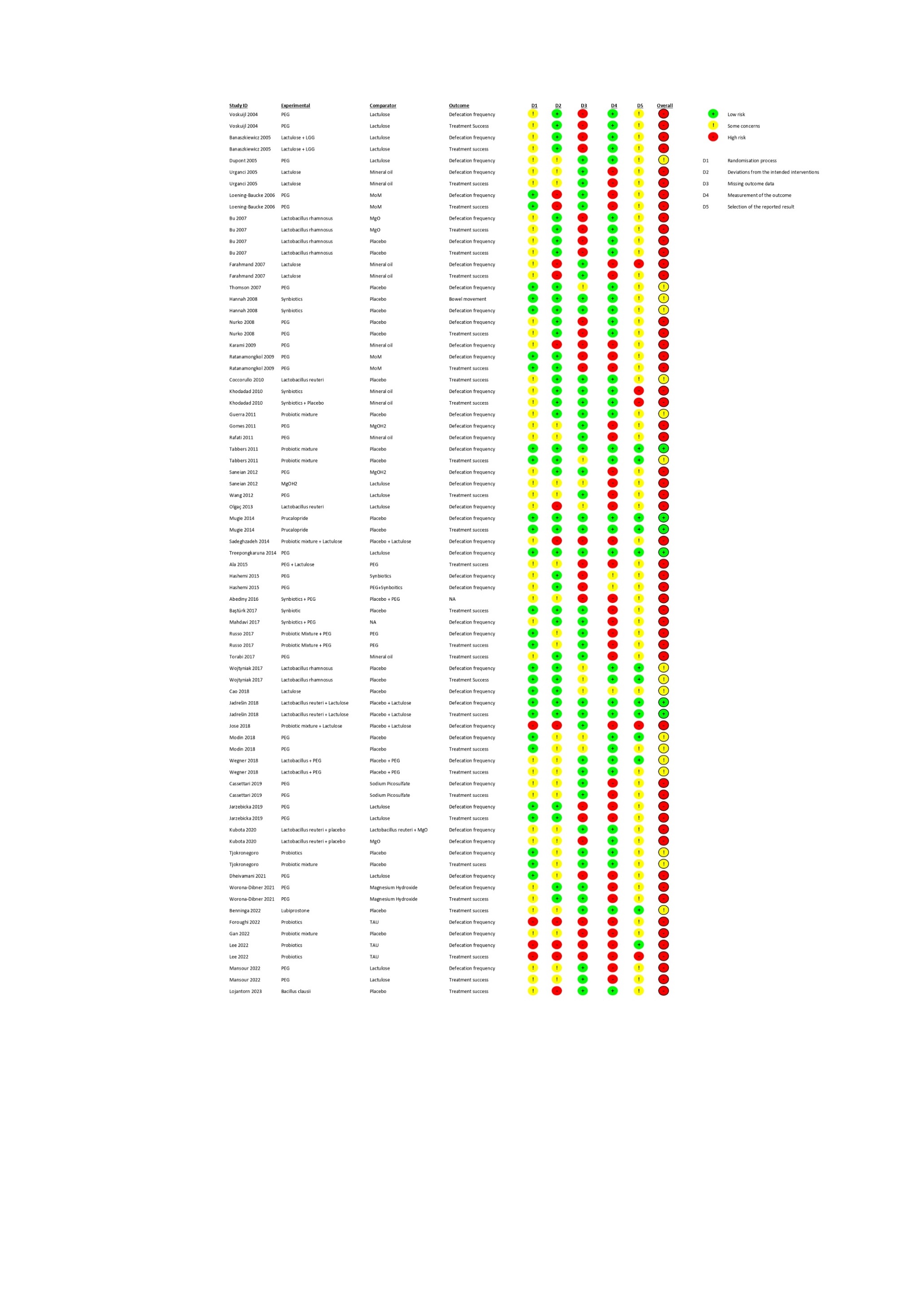
****Supplementary Figure S3** Overview of ROB assessments according to the Cochrane ROB2 tool for all studies included in the present systematic review.


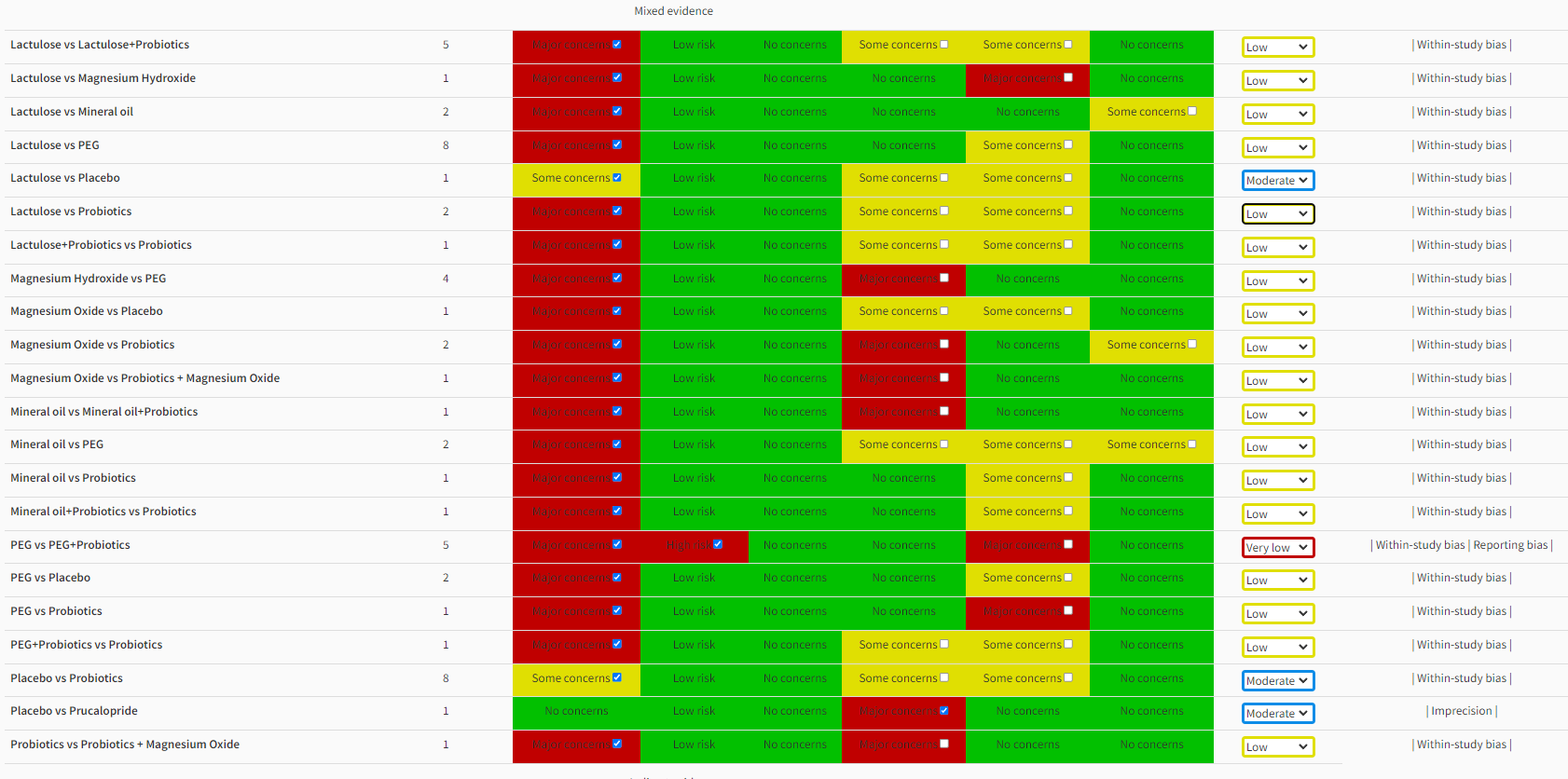

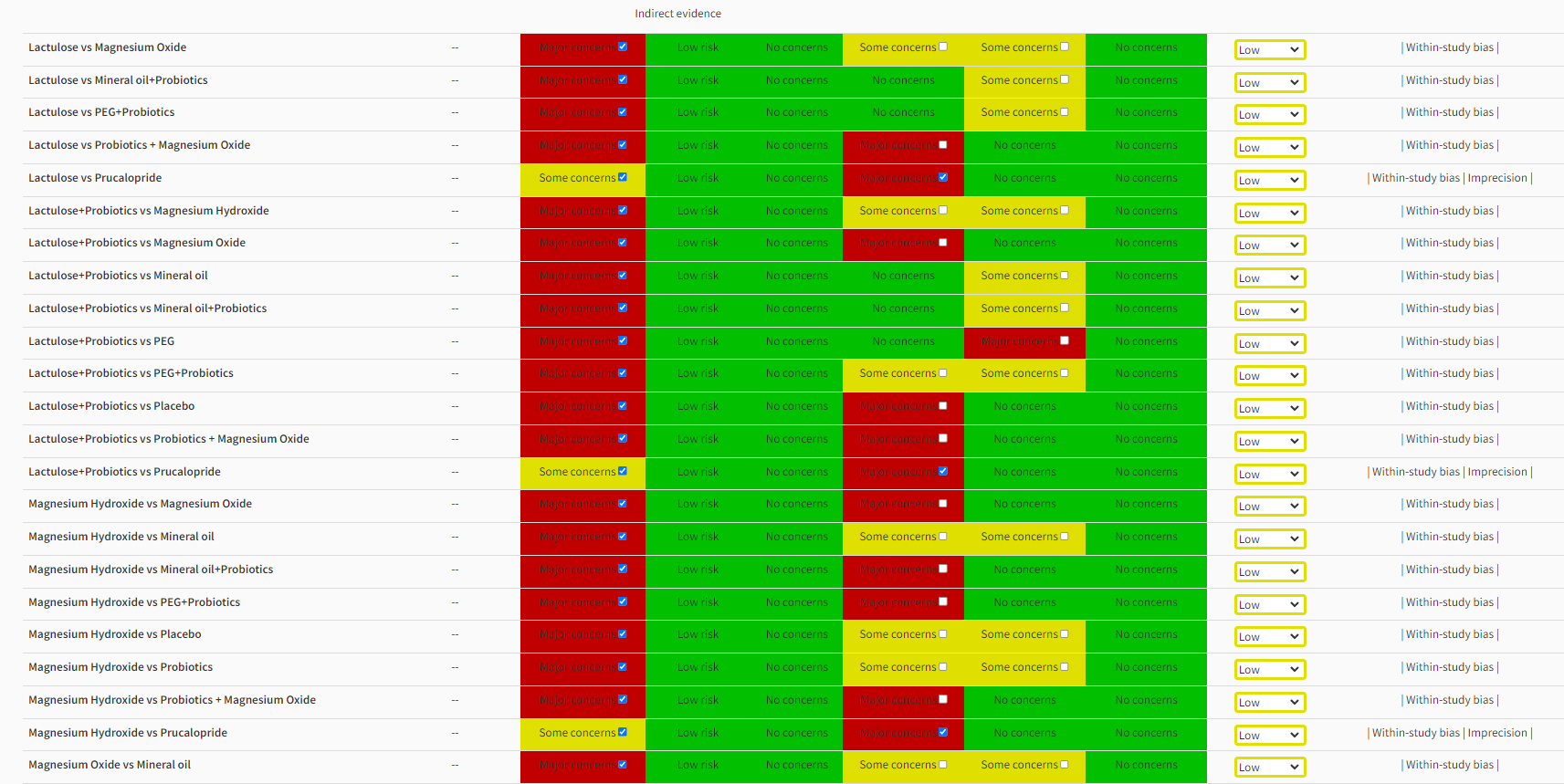

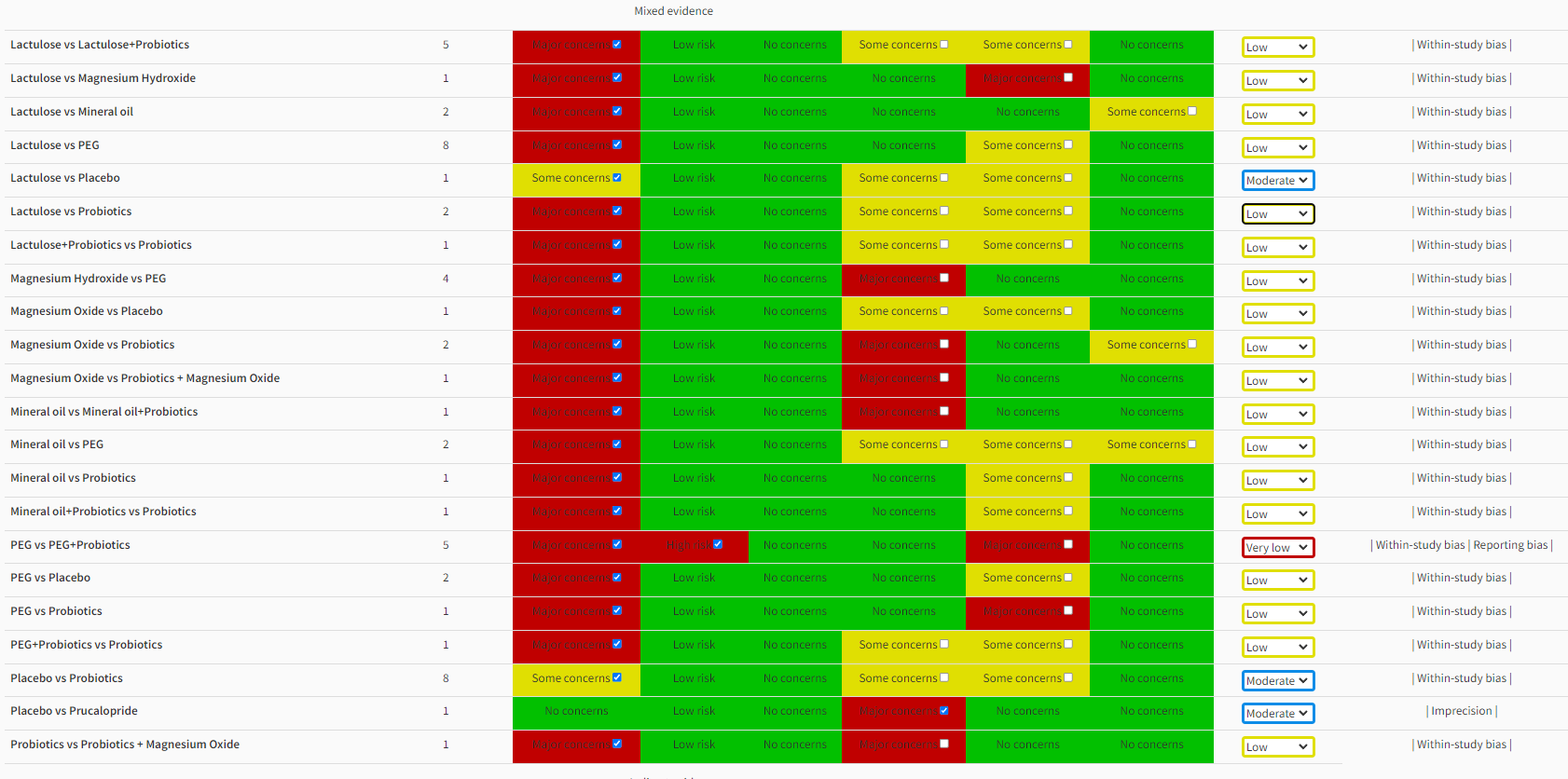


## **Supplementary Figure S4** Confidence in the estimates of the comparisons in the network of defecation frequency using CINeMA.


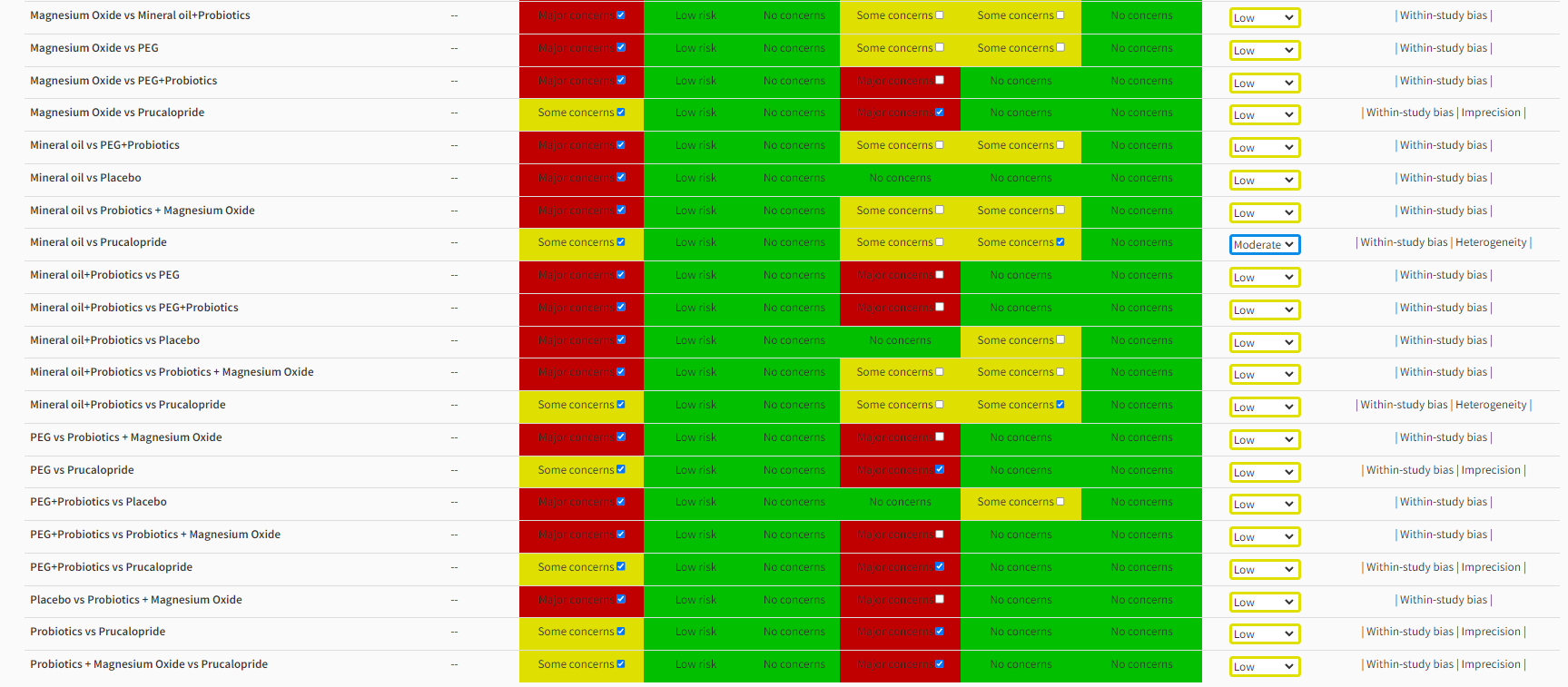


Supplementary Figure S4 **Continued**


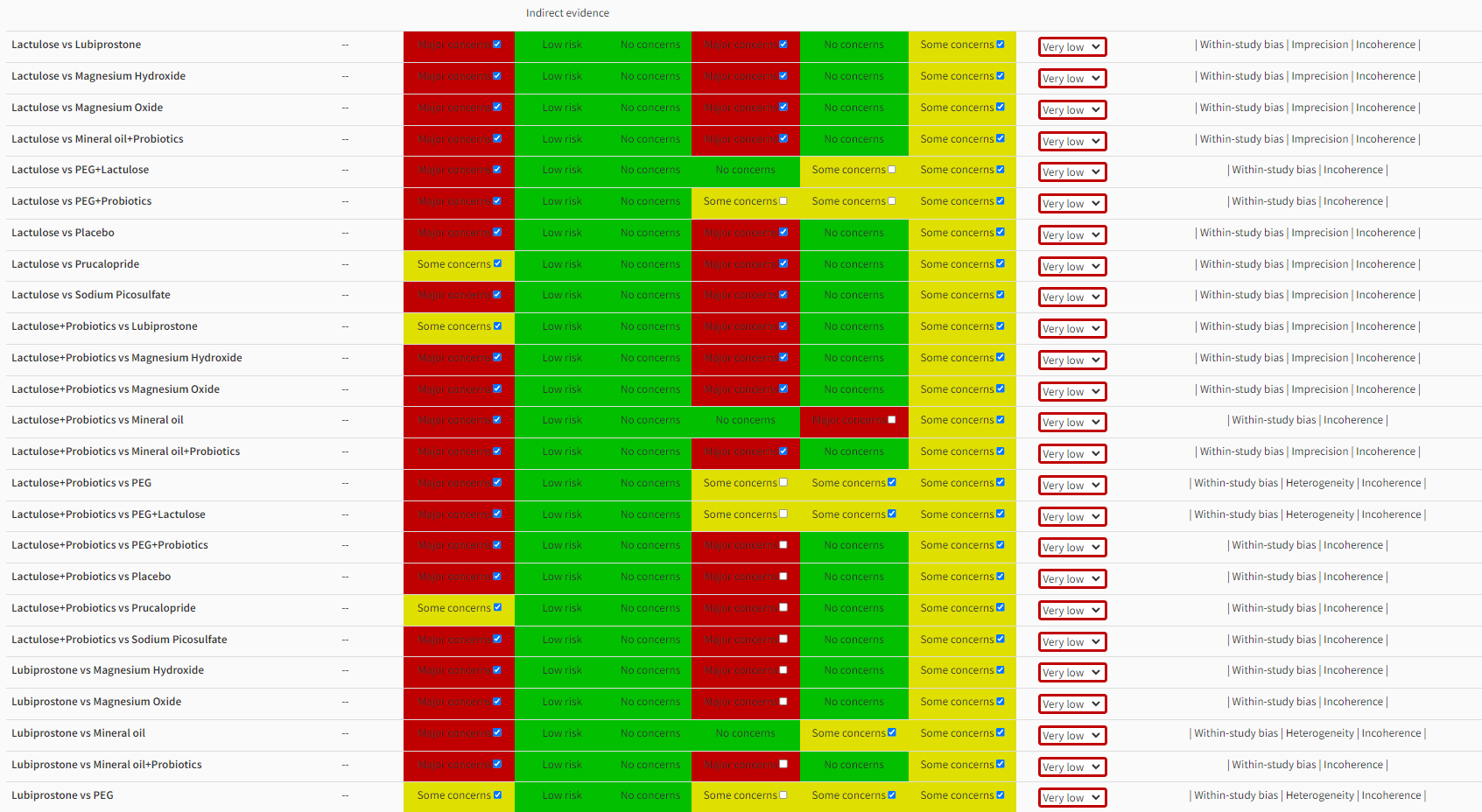

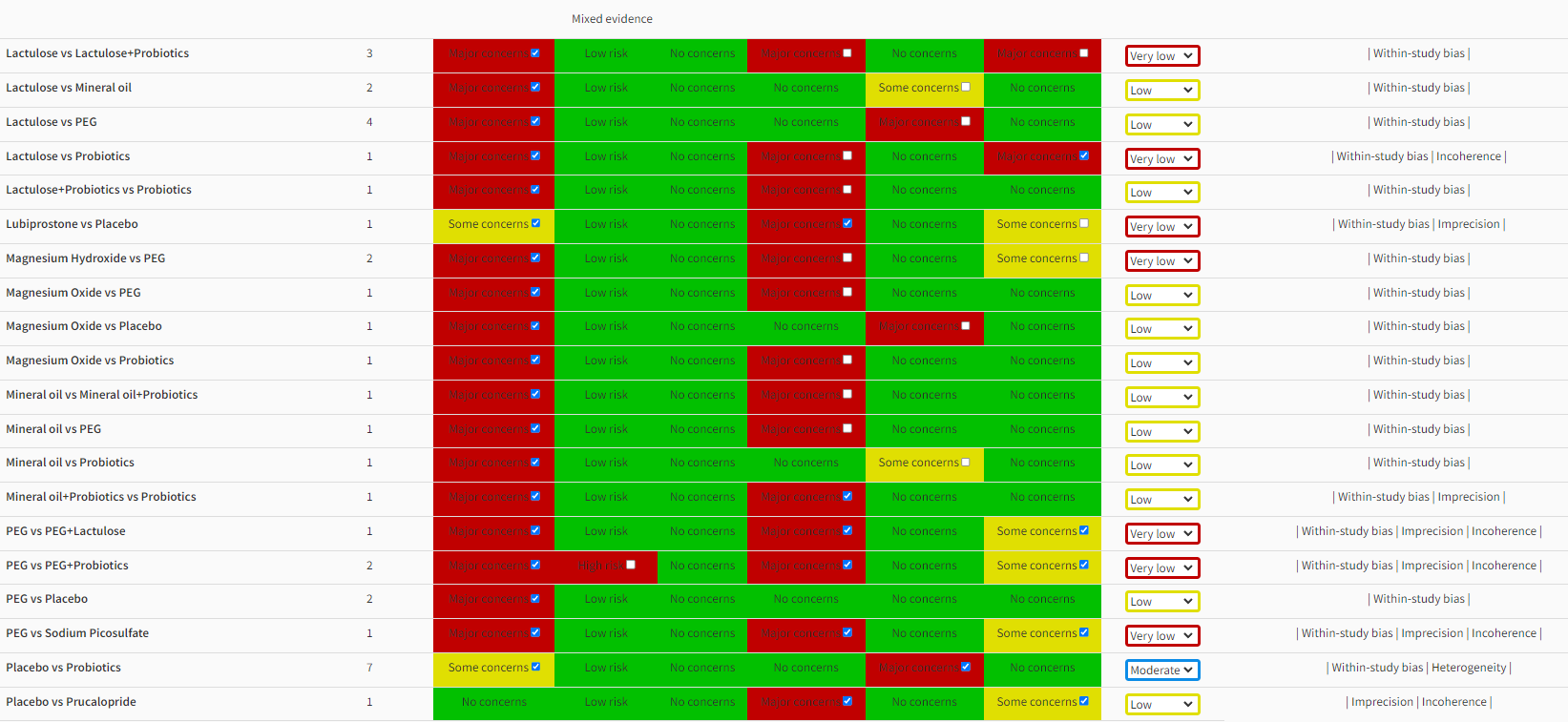


## **Supplementary Figure S5** Confidence in the estimates of the comparisons in the network of treatment success using CINeMA.


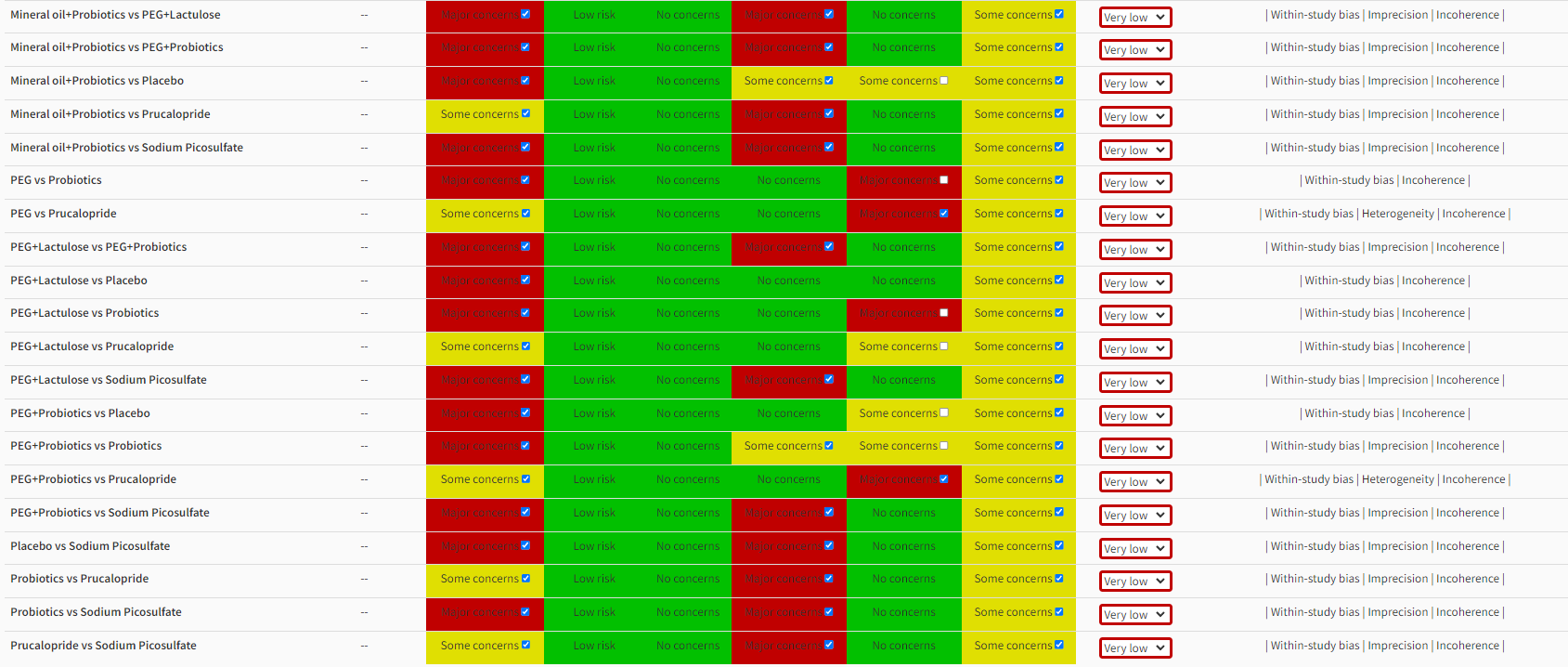

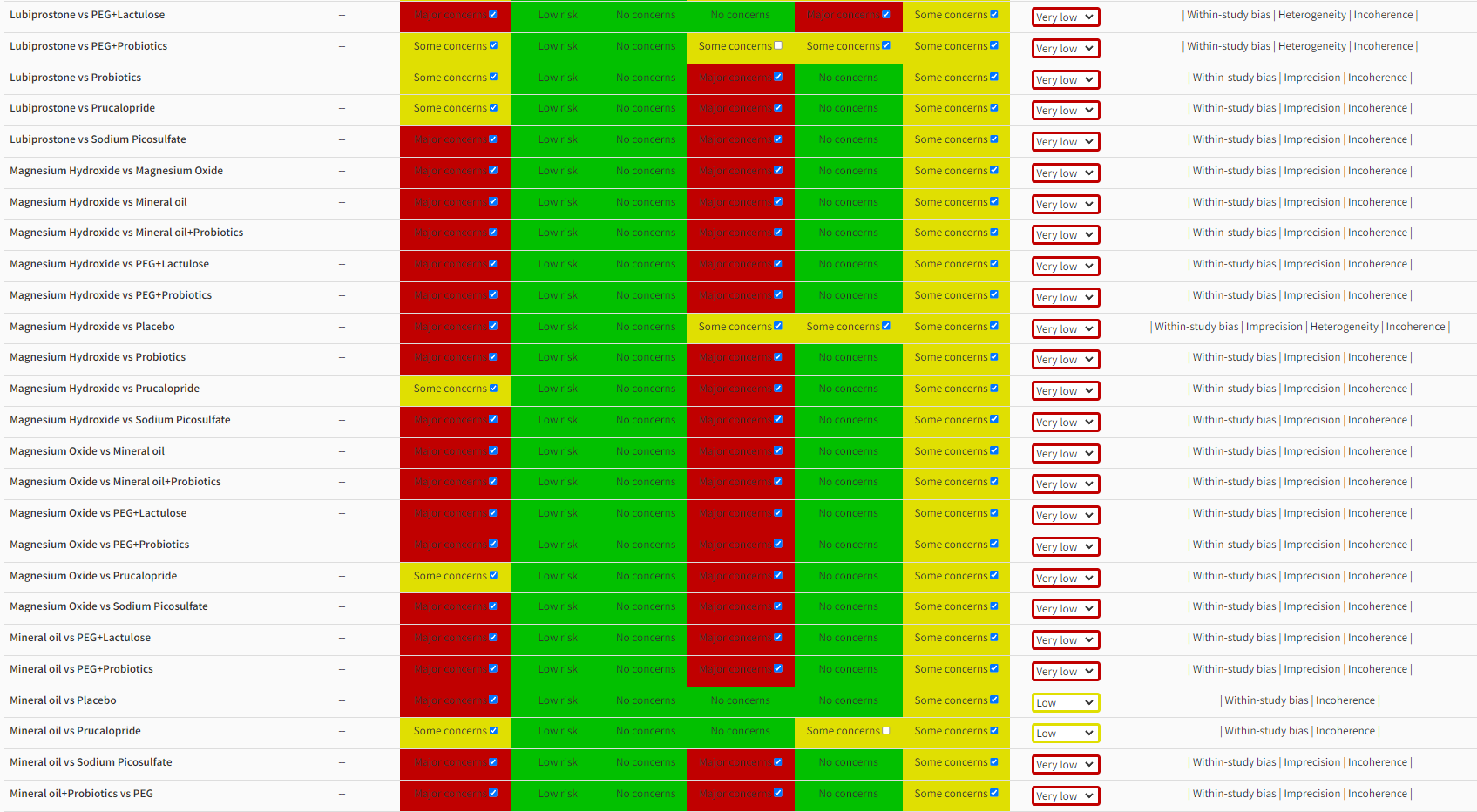


Supplementary Figure S5 **Continued**

**
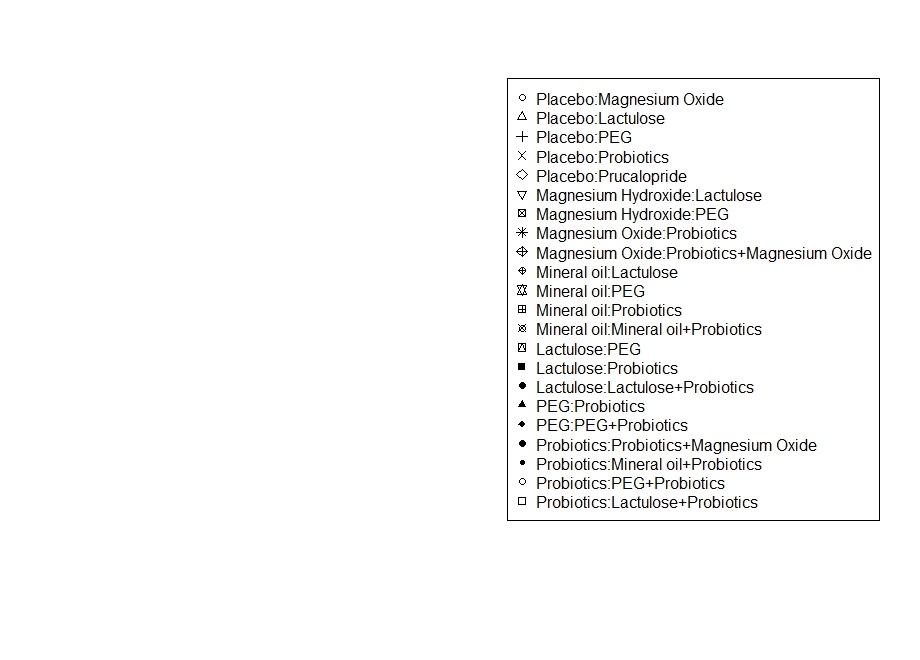

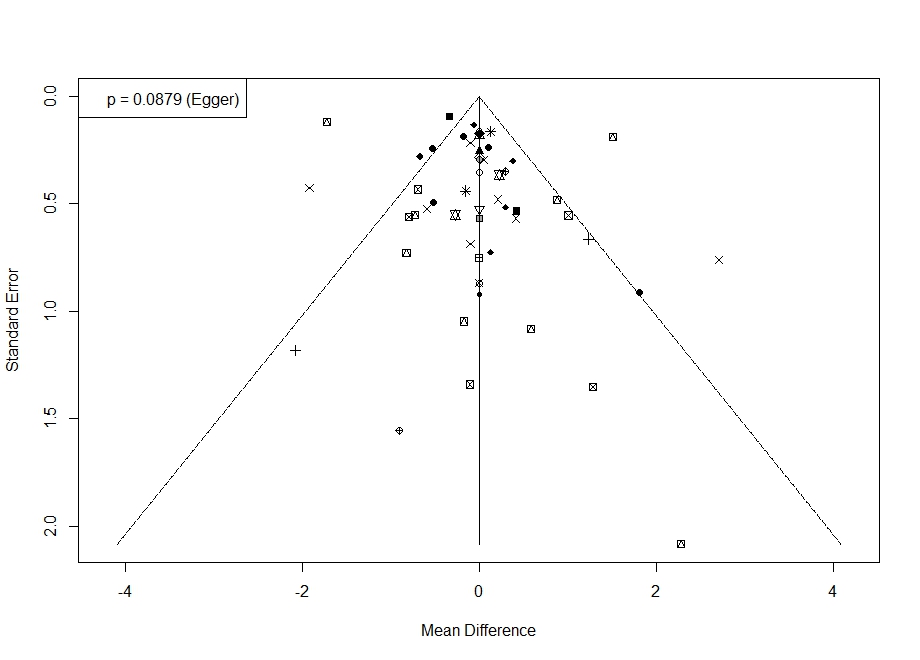
**

**Supplementary Figure S6** Comparison-adjusted funnel plot depicting the effect size by standard error for each comparison contributing to the analysis of defecation frequency.

The comparison-adjusted funnel plot was constructed by first specifying the order of treatments from oldest to newest based on the assumption that newer treatments are favored in small trials. In this way, funnel plot asymmetry with missing small studies on the right side of the line of no effect suggest that small-study effects favor newer treatments. The order specified is:

Placebo, Magnesium Hydroxide, Magnesium Oxide, Mineral oil, Lactulose, PEG, Probiotics, Probiotics+Magnesium Oxide, Mineral oil+Probiotics, PEG+Probiotics, Lactulose+Probiotics, Prucalopride.


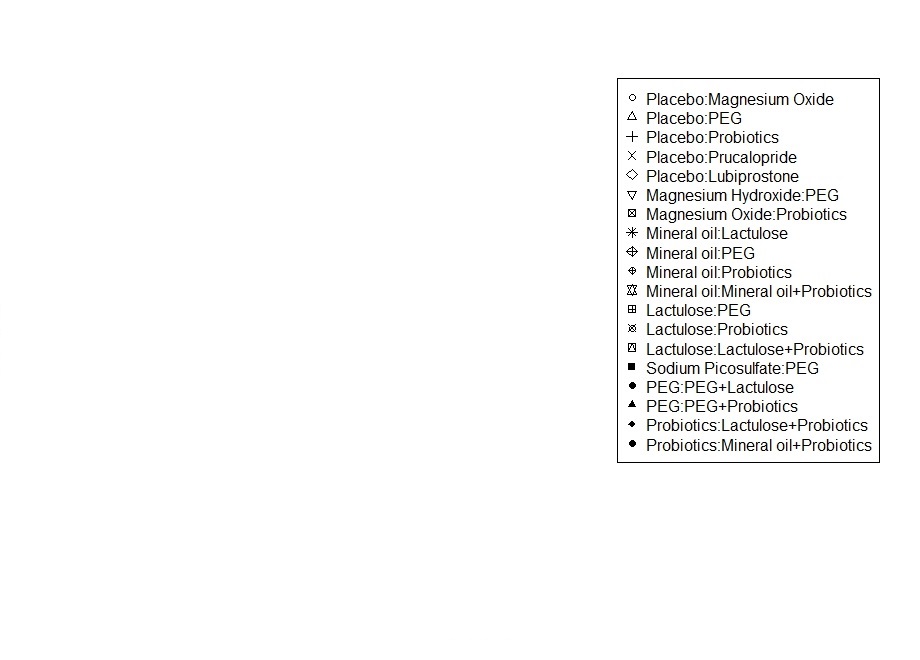
**
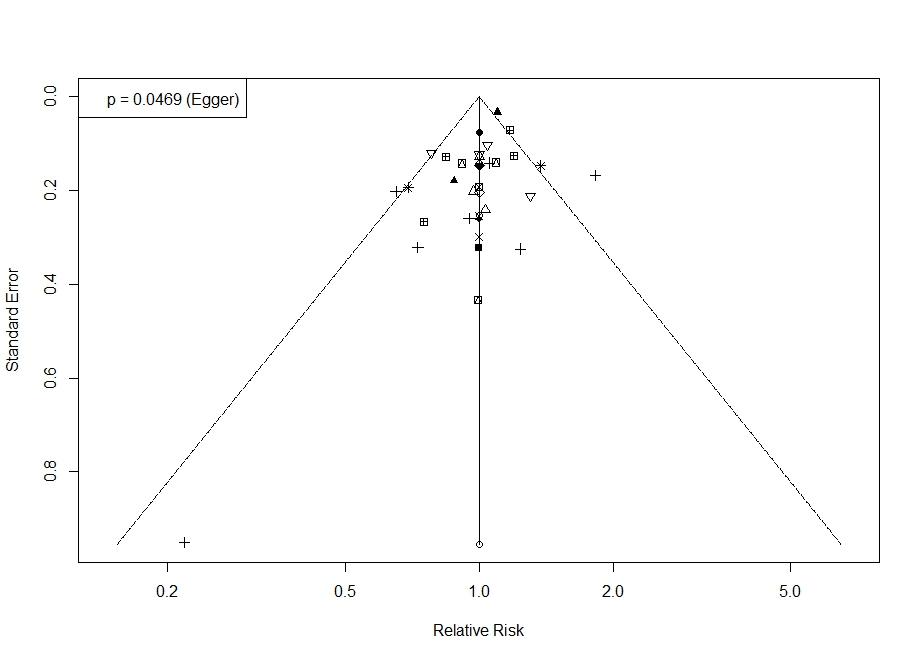
**

## **Supplementary Figure S7** Comparison-adjusted funnel plot depicting the effect size by standard error for each comparison contributing to the analysis of treatment success.

The comparison-adjusted funnel plot was constructed by first specifying the order of treatments from oldest to newest based on the assumption that newer treatments are favored in small trials. In this way, funnel plot asymmetry with missing small studies on the right side of the line of no effect suggest that small-study effects favor newer treatments.

The order specified is:

Placebo, Magnesium Hydroxide, Magnesium Oxide, Mineral oil, Lactulose, Sodium Picosulfate, PEG, Probiotics, Lactulose+Probiotics, Mineral oil+Probiotics, PEG+Lactulose, PEG+Probiotics, Prucalopride, Lubiprostone.

## **References**

S1. Abediny M, Ataiee P, Afkhamzadeh A, Seifmanesh M, Sedaghat B. The effect of probiotics on the treatment of functional constipation in children of 4–12 years of age. *J Isfahan Med Sch*. 2016;33(368):2448-2454.

S2. Bekkali NLH, Hoekman DR, Liem O, et al. Polyethylene Glycol 3350 With Electrolytes Versus Polyethylene Glycol 4000 for Constipation: A Randomized, Controlled Trial. *J Pediatr Gastroenterol Nutr*. 2018;66(1):10-15.

S3. Benninga MA, Hussain SZ, Sood MR, et al. Lubiprostone for Pediatric Functional Constipation: Randomized, Controlled, Double-Blind Study With Long-term Extension. *Clin Gastroenterol Hepatol*. 2022;20(3):602-610.e5.

S4. Candy DCA, Edwards D, Geraint M. Treatment of faecal impaction with polyethelene glycol plus electrolytes (PGE + E) followed by a double-blind comparison of PEG + E versus lactulose as maintenance therapy. *J Pediatr Gastroenterol Nutr*. 2006;43(1):65-70.

S5. Gan D, Chen J, Tang X, et al. Impact of a probiotic chewable tablet on stool habits and microbial profile in children with functional constipation: A randomized controlled clinical trial. *Front Microbiol*. 2022;13:985308.

S6. Hakimzadeh M, Mottaghi S, Ahmadi M, Javaherizadeh H. Effect of polyethylene glycol versus lactulose on abdominal pain in children occult constipation: a randomized controlled study. *Rev Gastroenterol Peru*. 2019;39(4):323-328. Effect of polyethylene glycol versus lactulose on abdominal pain in children occult constipation: a randomized controlled study.

S7. Hashemi M, Javaheri J, Habibi M, Naziri M. Comparing the effect of probiotics and polyethylene glycol in treatment of children with functional constipation. *J Arak Uni Med Sci*. 2015;18(6):78-85.

S8. Jadrešin O, Sila S, Trivic I, Mišak Z, Hojsak I, Kolacek S. Lack of Benefit of Lactobacillus reuteri DSM 17938 as an Addition to the Treatment of Functional Constipation. *J Pediatr Gastroenterol Nutr*. 2018;67(6):763-766.

S9. Jarzebicka D, Sieczkowska-Golub J, Kierkus J, et al. PEG 3350 Versus Lactulose for Treatment of Functional Constipation in Children: Randomized Study. *J Pediatr Gastroenterol Nutr*. 2019;68(3):318-324.

S10. Kasiri K, Sedehi M, Mortazavi S. Comparison of the effect of polyethylene glycol and simultaneous administration of polyethylene glycol with probiotics in the treatment of chronic functional constipation: a clinical trial. *J Shahrekord Univ Med Sci*. 2020;22(3):135-140.

S11. Khodadad A, Sabbaghian M. Role of synbiotics in the treatment of childhood constipation: a double-blind randomized placebo controlled trial. *Iran J Pediatr*. 2010;20(4):387-392.

S12. Kubota M, Ito K, Tomimoto K, et al. Lactobacillus reuteri DSM 17938 and Magnesium Oxide in Children with Functional Chronic Constipation: A Double-Blind and Randomized Clinical Trial. *Nutrients*. 2020;12(1):225.

S13. Lojanatorn P, Phrommas J, Tanpowpong P, Getsuwan S, Lertudomphonwanit C, Treepongkaruna S. Efficacy of Bacillus clausii in pediatric functional constipation: A pilot of a randomized, double-blind, placebo-controlled trial. *Indian Pediatr*. 2023;60(6):453-458.

S14. Mahdavi M, Esmaeili Dooki MR, Mehrabani S, Hajiahmadi M, Moghadamnia AA, Moslemi L. The effect of adding synbiotics to polyethylene glycol in childhood functional constipation: A randomized clinical trial study. *Int J Pediatr*. 2017;5(7):5357-5367.

S15. Modin L, Walsted AM, Dalby K, Jakobsen MS. Polyethylene Glycol Maintenance Treatment for Childhood Functional Constipation: A Randomized, Placebo-controlled Trial. *J Pediatr Gastroenterol Nutr*. 2018;67(6):732-737.

S16. Mugie SM, Korczowski B, Bodi P, et al. Prucalopride Is No More Effective Than Placebo for Children With Functional Constipation. *Gastroenterology*. 2014;147(6):1285-1295.e1.

S17. Saneian H, Tavakkol K, Adhamian P, Gholamrezaei A. Comparison of Lactobacillus Sporogenes plus mineral oil and mineral oil alone in the treatment of childhood functional constipation. *J Res Med Sci*. 2013;18(2):85-88.

S18. Savino F, Viola S, Erasmo M, Di Nardo G, Oliva S, Cucchiara S. Efficacy and tolerability of peg-only laxative on faecal impaction and chronic constipation in children. A controlled double blind randomized study vs a standard peg-electrolyte laxative. *BMC Pediatrics*. 2012;12178.

S19. Tabbers MM, Chmielewska A, Roseboom MG, et al. Fermented milk containing Bifidobacterium lactis DN-173 010 in childhood constipation: A randomized, double-blind, controlled trial. *Pediatrics*. 2011;127(6):e1392-e1399.

S20. Thomson MA, Jenkins HR, Bisset WM, et al. Polyethylene glycol 3350 plus electrolytes for chronic constipation in children: a double blind, placebo controlled, crossover study. *Arch Dis Child*. 2007;92(11):996-1000.

S21. Treepongkaruna S, Simakachorn N, Pienvichit P, et al. A randomised, double-blind study of polyethylene glycol 4000 and lactulose in the treatment of constipation in children. *BMC Pediatr*. 2014;14:153.

S22. Connolly P, Hughes IW, Ryan GW. Comparison of 'duphalac' and 'irritant' laxatives during and after treatment of chronic constipation: A preliminary study. *Curr Med Res Opin*. 1975;2(10):620-625.

S23. Perkin JM. Constipation in childhood: A controlled comparison between lactulose and standardized senna. *Curr Med Res Opin*. 1977;4(8):540-543.

S24. Bass P, Dennis S. The laxative effects of lactulose in normal and constipated subjects. *J Clin Gastroenterol*. 1981;3 (1):23-8.

S25. Sondheimer JM, Gervaise EP. Lubricant versus laxative in the treatment of chronic functional constipation of children: A comparative study. *J Pediatr Gastroenterol Nutr*. 1982;1(2):223-226.

S26. Gallet JP. Importal® (Lactilol). Results of a study of the treatment of constipation in children. *Revue de Pediatrie*. 1990;26(8):275-279.

S27. Martino AM, Pesce F, Rosati U. The effects of lactitol in the treatment of intestinal stasis in childhood. *Minerva Pediatr*. 1992;44(6):319-23.

S28. Pitzalis G, Deganello F, Mariani P, et al. Lactitole in chronic idiopathic constipation of childhood. *Pediatr Med Chir*. 1995;17(3):223-226.

S29. Muller M, Sirot Jaquenoud E. Treatment of chronic constipation in children with lactulose. *Ars Medici*. 1994;84(8):568-570+573.

S30. Dupont C, Ammar F, Leluyer B, Mathiex-Fortunet H, Garnier P. Polyethylene glycol (PEG) 4000 in constipated children (6 months-15 years): a dose determination study. *J Pediatr Gastroenterol Nutr*. 2000;31(2):S95.

S31. Dupont C, Leluyer B, Amar F, et al. A dose determination study of polyethylene glycol 4000 in constipated children: Factors influencing the maintenance dose. *J Pediatr Gastroenterol Nutr*. 2006;42(2):178-185.

S32. Gremse D, Hixon J. Comparison of polyethylene glycol 3350, NF powder and lactulose for treatment of chronic constipation in children. *J Pediatr Gastroenterol Nutr*. 2000;31(2):S131.

S33. Gremse DA, Hixon J, Crutchfield A. Comparison of polyethylene glycol 3350 and lactulose for treatment of chronic constipation in children. *Clin Pediatr*. 2002;41(4):225-229.

S34. Tozzi A, Pensabene L, Miele E, Di Meo M, Staiano A, Strisciuglio P. Polyethylene glycol vs. lactitole in the treatment of functional constipation in children: a comparative study. *J Pediatr Gastroenterol Nutr*. 2000;31(2):S178.

S35. Loening-Baucke V. Polyethylene glycol without electrolytes for children with constipation and encopresis. *J Pediatr Gastroenterol Nutr*. 2002;34(4):372-377.

S36. Sloots CE, Poen AC, Kerstens R, et al. Effects of prucalopride on colonic transit, anorectal function and bowel habits in patients with chronic constipation. *Aliment Pharmacol Ther*. 2002;16(4):759-67.

S37. Candy DCA, Edwards D. A double-blind randomised study to compare the safety and efficacy of Movicol (Trademark) and lactulose for maintenance therapy in childhood constipation (Abstract). *Proceedings of the 2nd european pediatric GI motility meeting, 23-26 apr 2003, bruges, belgium*. 2003;

S38. Voskuijl W, de Lorin F, Verwijs W, et al. PEG 3350 versus lactulose in the treatment of childhood constipation. A double blind, randomized controlled trial. *J Pediatr Gastroenterol Nutr*. 2003;36(4):528.

S39. Voskuijl W, de Lorijn F, Verwijs W, et al. PEG 3350 (Transipeg) versus lactulose in the treatment of childhood functional constipation: a double blind, randomised, controlled, multicentre trial. *Gut*. 2004;53(11):1590-1594.

S40. Kinservik MA, Friedhoff MM. The efficacy and safety of polyethylene glycol 3350 in the treatment of constipation in children. *Pediatric nursing*. 2004;30(3):232-237.

S41. Shevtsov SA. Clinical efficacy of duphalac in the treatment of functional constipations. *Eksperimental'naia i klinicheskaia gastroenterologiia = Experimental & clinical gastroenterology*. 2005;(6):58-60.

S42. Bekkali NLH, Bongers MEJ, Van Den Berg MM, Liem O, Benninga MA. The role of a probiotics mixture in the treatment of childhood constipation: A pilot study. *Nutr J*. 2007;617.

S43. Uhm JH. Comparison of polyethylene glycol 4000 and lactulose for treatment of chronic functional constipation in children. *Korean J Pediatr*. 2007;50(8):752‐756.

S44. Camilleri M, Kerstens R, Rykx A, Vandeplassche L. A placebo-controlled trial of prucalopride for severe chronic constipation. *N Engl J Med*. 2008;358(22):2344-54.

S45. Coccorullo P, Martinelli M, Miele E, Tramontano A, Greco L, Staiano A. Lactobacillus reuteri in infants with functional chronic constipation: A double-blind, randomized, placebo-controlled study. *Dig Liver Dis*. 2009;41S(S3):S227.

S46. Hyman PE, Ueno R. Lubiprostone for the treatment of functional constipation in children and adolescents. *J Pediatr Gastroenterol Nutr*. 2009;49:E34.

S47. Hyman PE, Di Lorenzo C, Prestridge LL, Youssef NN, Ueno R. Lubiprostone for the treatment of functional constipation in children. *J Pediatr Gastroenterol Nutr*. 2014;58(3):283-291.

S48. Quigley EM, Vandeplassche L, Kerstens R, Ausma J. Clinical trial: the efficacy, impact on quality of life, and safety and tolerability of prucalopride in severe chronic constipation--a 12-week, randomized, double-blind, placebo-controlled study. *Aliment Pharmacol Ther*. 2009;29(3):315-28.

S49. Tack J, Van Outryve M, Beyens G, Kerstens R, Vandeplassche L. Prucalopride (Resolor) in the treatment of severe chronic constipation in patients dissatisfied with laxatives. *Gut*. 2009;58(3):357-365.

S50. Bae SH, Son JS, Lee R. Effect of fluid intake on the outcome of constipation in children: PEG 4000 versus lactulose. *Pediatrics International*. 2010;52(4):594-597.

S51. Camilleri M, Van Outryve MJ, Beyens G, Kerstens R, Robinson P, Vandeplassche L. Clinical trial: The efficacy of open-label prucalopride treatment in patients with chronic constipation - Follow-up of patients from the pivotal studies. *Aliment Pharmacol Ther*. 2010;32(9):1113-1123.

S52. Farahmand F, Eftekhari K, Modarresi V, Najafi-Sani M, Khodadad A, Motamed F. Comparing oral route paraffin oil versus rectal route for disimpaction in children with chronic constipation; a randomized control trial. *Iran J Pediatr*. 2010;20(3):291-296.

S53. Lembo AJ, Kurtz CB, Macdougall JE, et al. Efficacy of linaclotide for patients with chronic constipation. *Gastroenterology*. 2010;138(3):886-95.e1.

S54. Lembo AJ, Schneier HA, Shiff SJ, et al. Two randomized trials of linaclotide for chronic constipation. *N Engl J Med*. 2011;365(6):527‐536.

S55. Tabbers M, Chmielewska AM, Roseboom M, Reitsma J, Szajewska H, Benninga MA. 908 Fermented Dairy Product Containing Bifidobacterium Lactis Dn-173 010 in Treatment of Childhood Constipation: A Randomized, Double-Blind, Placebo-Controlled Trial. *Gastroenterology*. 2010;138(5):S-129.

S56. Tabbers MM, de Milliano I, Roseboom MG, Benninga MA. Is Bifidobacterium breveeffective in the treatment of childhood constipation? Results from a pilot study. *Nutr J*. 2011;10(1):19.

S57. Gheibi S, Hadi Imanieh M, Haghighat M, et al. Comparison of the low dose polyethylene glycol with lactulose and magnesium hydroxide in constipated children. A multicentric randomized clinical trial. *Life Sci J*. 2012;9(4):5344-5350.

S58. Parzȩcka M, Jaskólska A. The role of probiotics in the treatment of functional constipation in children and adolescents - Own observations. *Gastroenterologia Polska*. 2012;19(4):162-165.

S59. Savino F, Viola S, Erasmo M, Di Nardo G, Oliva S, Cucchiara S. Efficacy and tolerability of peg-only laxative on faecal impaction and chronic constipation in children. A controlled double blind randomized study vs a standard peg-electrolyte laxative. *BMC Pediatrics*. 2012;12(1):178.

S60. Cinca R, Chera D, Gruss HJ, Halphen M. Randomised clinical trial: Macrogol/PEG 3350+electrolytes versus prucalopride in the treatment of chronic constipation - A comparison in a controlled environment. *Aliment Pharmacol Ther*. 2013;37(9):876-886.

S61. Treepongkaruna S, Simakachorn N, Pienvichit P, et al. Efficacy of a polyethylene glycol laxative (PEG, Macrogol 4000) versus lactulose for the treatment of chronic constipation in children. Results of a randomized, double-blind controlled study performed in Thailand. *Gastroenterology*. 2013;144(5):S547.

S62. Winter HS, Di Lorenzo C, Benninga MA, et al. Oral Prucalopride in Children With Functional Constipation. *J Pediatr Gastroenterol Nutr*. 2013;57(2)

S63. Benninga MA, Mugie SM, Korczowski B, et al. Efficacy and safety of prucalopride in children with functional constipation: Results of a multicenter, placebo-controlled, phase 3 trial. *Gastroenterology*. 2014;146(5):S-172.

S64. Dziechciarz P, Horvath A, Szajewska H. Polyethylene glycol 4000 for treatment of functional constipation in children: Randomized trial of two different doses. *J Pediatr Gastroenterol Nutr*. 2014;

S65. Magro DO, de Oliveira LMR, Bernasconi I, et al. Effect of yogurt containing polydextrose, Lactobacillus acidophilus NCFM and Bifidobacterium lactis HN019: a randomized, double-blind, controlled study in chronic constipation. *Nutr J*. 2014;13(1):75.

S66. Tack J, Stanghellini V, Dubois D, Joseph A, Vandeplassche L, Kerstens R. Effect of prucalopride on symptoms of chronic constipation. *Neurogastroenterol Motil*. 2014;26(1):21-27.

S67. Dziechciarz P, Horvath A, Szajewska H. Polyethylene glycol 4000 for treatment of functional constipation in children. *J Pediatr Gastroenterol Nutr*. 2015;60(1):65-68.

S68. Russo M, Giugliano F, Quitadamo P, Miele E, Staiano A. Efficacy of a mixture of probiotic agents, including bifidobacterium breve, infantis and longum, as complementary therapy for chronic functional constipation in childhood. *Dig Liver Dis*. 2015;47:e240.

S69. Russo M, Giugliano FP, Quitadamo P, Mancusi V, Miele E, Staiano A. Efficacy of a mixture of probiotic agents as complementary therapy for chronic functional constipation in childhood. *Ital J Pediatr*. 2017;43(1):24.

S70. Wegner A, Banaszkiewicz A, Kierkus J, et al. Effectiveness of lactobacillus reuteri in the treatment of functional constipation in children: a randomized, double-blind, placebo-controlled, multicenter trial. *United European Gastroenterol J*. 2015;3(5):A20‐.

S71. Wegner A, Banaszkiewicz A, Kierkus J, et al. The effectiveness of Lactobacillus reuteri DSM 17938 as an adjunct to macrogol in the treatment of functional constipation in children. A randomized, double-blind, placebo-controlled, multicentre trial. *Clin Res Hepatol Gastroenterol*. 2018;42(5):494-500.

S72. Basturk A, Artan R, Yilmaz A. Efficacy of synbiotic, probiotic, and prebiotic treatments for irritable bowel syndrome in children: A randomized controlled trial. *J Pediatr Gastroenterol Nutr*. 2016;62:421.

S73. Baştürk A, Artan R, Yılmaz A. Efficacy of synbiotic, probiotic, and prebiotic treatments for irritable bowel syndrome in children: A randomized controlled trial. *Turk J Gastroenterol*. 2016;27(5):439-443.

S74. Chao HC, Hsu YC, Chen SY, Chen CC. Impact of probiotics on constipation and intestinal microflora in children with functional constipation. *J Pediatr Gastroenterol Nutr*. 2016;63:S37.

S75. Jordan-Ely J, Dughetti LD, Dobson K, et al. Disimpaction for children with palpable faecalomas using polyethelene glycol and sodium picosulphate. *Journal of Gastroenterology and Hepatology (Australia)*. 2016;31:178-179.

S76. Jarzebicka D, Sieczkowska J, Kierkus J, et al. Multicenter study evaluating the efficacy of polyethylene glycol (PEG) 3350 (Dicopeg Junior) in comparison with lactulose for the treatment of functional constipation in children aged 6 months to 6 years. A prospective, randomized study -preliminary report. *J Pediatr Gastroenterol Nutr*. 2017;64:370‐371.

S77. Jarzebicka D, Sieczkowska-Golub J, Kierkus J, et al. Peg 3350 vs lactulose for treatment of functional constipation in children aged 6 months to 6 years a multicenter, prospective, randomized study. *J Pediatr Gastroenterol Nutr*. 2017;65:S109-S110.

S78. Miner PB, Jr., Koltun WD, Wiener GJ, et al. A Randomized Phase III Clinical Trial of Plecanatide, a Uroguanylin Analog, in Patients with Chronic Idiopathic Constipation. *Am J Gastroenterol*. 2017;112(4):613-621.

S79. Modin L, Jakobsen M. Polyethylene glycol maintenance treatment in childhood functional constipation: A doubleblind, randomized, placebo-controlled trail. *J Pediatr Gastroenterol Nutr*. 2017;64:374.

S80. Wojtyniak K, Horvath A, Dziechciarz P, H S. Lactobacillus casei rhamnosus Lcr35 in the management of functional constipation in children: a randomized trial. *J Pediatr Gastroenterol Nutr*. 2017;64(S1):414.

S81. Wojtyniak K, Horvath A, Dziechciarz P, Szajewska H. Lactobacillus casei rhamnosus Lcr35 in the management of functional constipation in children: a randomized trial. *J Pediatr*. 2017;184:101-105.e1.

S82. Acharyya B, Acharyya S, Bhattacharya C. Comparative analysis of PEG +E with stimulant laxative versus PEG+E alone for disimpaction regimen-A study from a tertiary centre in Eastern India. *J Pediatr Gastroenterol Nutr*. 2018;66:330-331.

S83. Benninga MA, Clifford RA, O'Gorman M, Losch-Beridon T, Mareya SM, Lorenzo CD. Long-term open-label safety extension study assessing oral lubiprostone in children with pediatric functional constipation aged 6-17 years. *Gastroenterology*. 2018;154(6):S-560.

S84. Benninga MA, Hussain SZ, Sood MR, et al. Efficacy and Safety of Lubiprostone in Children with Functional Constipation: A Multicenter, Randomized, Placebo-Controlled, Double-Blind Pivotal Study. *Gastroenterology*. 2018;154(6):S-559.

S85. Kubota M, Kuroki H, Kubota A, et al. Therapeutic efficacy of probiotic Lactobacillus reuteri DSM 17938 and concomitant changes of gut microbiota in infants with functional chronic constipation. *J Pediatr Gastroenterol Nutr*. 2018;66:441.

S86. Kumar K. A randomized study comparing the efficacy of Polyethylene Glycol 3350 versus Prucalopride in the management of chronic constipation. *Indian J Gastroenterol*. 2018;37:A47.

S87. Xinias I, Analitis A, Mavroudi A, et al. A Synbiotic Infant Formula with High Magnesium Content Improves Constipation and Quality of Life. *Pediatr Gastroenterol Hepatol Nutr*. 2018;21(1):28-33.

S88. Di Lorenzo C, Nurko S, Hyams JS, et al. Linaclotide safety and efficacy in children aged 6 to 17 years with functional constipation. *Am J Gastroenterol*. 2019;114:S645-S646.

S89. Hakimzadeh M, Mottaghi S, Ahmadi M, Javaherizadeh H. Effect of polyethylene glycol versus lactulose on abdominal pain in children occult constipation: a randomized controlled study. *Revista de gastroenterologia del Peru : organo oficial de la Sociedad de Gastroenterologia del Peru*. 2019;39(4):323-328.

S90. Mathew JL, Bhatnagar S. Polyethylene Glycol vs. Lactulose in Infants and Children with Functional Constipation. *Indian Pediatr*. 2019;56(5):415-419.

S91. Shatnawi MS, Alrwalah MM, Ghanma AM, Alqura'an ML, Zreiqat EN, Alzu'bi MM. Lactulose versus polyethylene glycol for disimpaction therapy in constipated children, a randomized controlled study. *Sudan J Paediatr*. 2019;19(1):31-36.

S92. Bae SH, Kim MR. Subtype classification of functional constipation in children: polyethylene glycol versus lactulose. *Pediatrics International*. 2020;62(7):816-819.

S93. Demir AM, Kuloglu Z, Kansu A. Treatment of childhood functional constipation: Comparison of senna, trimebutine and lactulose. *Turk J Pediatr Dis*. 2020;14(4):295-301.

S94. Olgac A, Sezer OB, Hosnut FO, Ozcay F. Lactobacillus reuteri dsm 17938 and quality of life associated with functional constipation. *Zdravniski Vestnik*. 2020;89(7-8):347-356.

S95. Cuffari C, Spalding W, Achenbach H, Thakur M, Gabriel A. Design of a phase 3, randomized, double-blind, placebo-controlled study to evaluate the efficacy and safety of prucalopride in pediatric patients with functional constipation. *J Pediatr Gastroenterol Nutr*. 2021;73(1 SUPPL 1):S107-S108.

S96. Hussain SZ, Labrum B, Mareya S, Stripling S, Clifford R. Safety of Lubiprostone in Pediatric Patients With Functional Constipation: A Nonrandomized, Open-Label Trial. *J Pediatr Gastroenterol Nutr*. 2021;73(5):572-578.

S97. Lee KJ, Ryoo E, Lee YM, et al. Effect of saccharomyces boulardii in combination with lactulose in children with functional constipation: Multicenter randomized controlled trial. *J Pediatr Gastroenterol Nutr*. 2021;72(SUPPL 1):490.

S98. Lee KJ, Ryoo E, Lee YM, et al. Saccharomyces boulardii and Lactulose for Childhood Functional Constipation: A Multicenter Randomized Controlled Trial. *J Neurogastroenterol Motil*. 2022;28(3):454-462.

S99. Nasri P, Saeidi S, Saneian H, et al. Comparative Evaluation between the LaxaPlus Barij(®) and Polyethylene Glycol (4000) in the Pediatric Functional Constipation in Children 2-15 Years Old. *J Res Pharm Pract*. 2021;10(4):180-184.

S100. Saneian H, Ghaedi S, Famouri F, et al. Comparing the effect of a herbal-based laxative (Goleghand®) and polyethylene glycol on functional constipation among children: A randomized controlled trial. *J Res Pharm Pract*. 2021;10(1):43.

S101. Tierney BT, Versalovic J, Fasano A, et al. Functional response to a microbial synbiotic in the gastrointestinal system of constipated children. 2022.

S102. Banaszkiewicz A, Szajewska H. Ineffectiveness of Lactobacillus GG as an adjunct to lactulose for the treatment of constipation in children: a double-blind, placebo-controlled randomized trial. *J Pediatr*. 2005;146(3):364-9.

S103. Dupont C, Leluyer B, Maamri N, et al. Double-blind randomized evaluation of clinical and biological tolerance of polyethylene glycol 4000 versus lactulose in constipated children. *J Pediatr Gastroenterol Nutr*. 2005;41(5):625-33.

S104. Urganci N, Akyildiz B, Polat TB. A comparative study: the efficacy of liquid paraffin and lactulose in management of chronic functional constipation. *Pediatr Int*. 2005;47(1):15-19.

S105. Loening-Baucke V, Pashankar DS. A randomized, prospective, comparison study of polyethylene glycol 3350 without electrolytes and milk of magnesia for children with constipation and fecal incontinence. *Pediatrics*. 2006;118(2):528-535.

S106. Bu LN, Chang MH, Ni YH, Chen HL, Cheng CC. Lactobacillus casei rhamnosus Lcr35 in children with chronic constipation. *Pediatr Int*. 2007;49(4):485-90.

S107. Farahmand F. A randomised trial of liquid paraffin versus lactulose in the treatment of chronic functional constipation in children. *Acta Medica Iranica*. 2007;45(3):183-188.

S108. Hannah H, Juffrie M, Soenarto SY. Effectiveness of synbiotics as laxative agent for constipation in children. *Paediatr Indones*. 2008;48(3):136-141.

S109. Nurko S, Youssef NN, Sabri M, et al. PEG3350 in the treatment of childhood constipation: a multicenter, double-blinded, placebo-controlled trial. *J Pediatr*. 2008;153(2):254-61, 261.e1.

S110. Karami H, Khademloo M, Niari P. Polyethylene glycol versus paraffin for the treatment of childhood functional constipation. *Iran J Pediatr*. 2009;19(3):255-261.

S111. Ratanamongkol P, Lertmaharit S, Jongpiputvanich S. Polyethylene glycol 4000 without electrolytes versus milk of magnesia for the treatment of Functional constipation in infants and young children: A randomized controlled trial. *Asian Biomedicine*. 2009;3(4):391-399.

S112. Coccorullo P, Strisciuglio C, Martinelli M, Miele E, Greco L, Staiano A. Lactobacillus reuteri (DSM 17938) in infants with functional chronic constipation: a double-blind, randomized, placebo-controlled study. *J Pediatr*. 2010;157(4):598-602.

S113. Guerra PV, Lima LN, Souza TC, et al. Pediatric functional constipation treatment with Bifidobacterium-containing yogurt: a crossover, double-blind, controlled trial. *World J Gastroenterol*. 2011;17(34):3916-3921.

S114. Gomes PB, Duarte MA, Melo Mdo C. Comparison of the effectiveness of polyethylene glycol 4000 without electrolytes and magnesium hydroxide in the treatment of chronic functional constipation in children. *J Pediatr (Rio J)*. 2011;87(1):24-8.

S115. Rafati M, Karami H, Salehifar E, Karimzadeh A. Clinical efficacy and safety of polyethylene glycol 3350 versus liquid paraffin in the treatment of pediatric functional constipation. *Daru*. 2011;19(2):154-158.

S116. Saneian H, Mostofizadeh N. Comparing the efficacy of polyethylene glycol (PEG), magnesium hydroxide and lactulose in treatment of functional constipation in children. *J Res Med Sci*. 2012;17(4):1-5.

S117. Wang Y, Wang B, Jiang X, et al. Polyethylene glycol 4000 treatment for children with constipation: A randomized comparative multicenter study. *Exp Ther Med*. 2012;3(5):853-856.

S118. Olgaç AMB, Sezer OB, Özçay F. Comparison of probiotic and lactulose treatments in children with functional constipation and determination of the effects of constipation treatment on quality of life. *Cocuk Sagligi ve Hastaliklari Dergisi*. 2013;56(1):1-7.

S119. Sadeghzadeh M, Rabieefar A, Khoshnevisasl P, Mousavinasab N, Eftekhari K. The effect of probiotics on childhood constipation: a randomized controlled double blind clinical trial. *Int J Pediatr*. 2014;2014:937212.

S120. Ala S, Haghighat M, Dehghani SM, Bazmamoun H. Response and recurrence rate after treatment with polyethylene glycol versus polyethylene glycol plus lactulose in children with chronic functional constipation: A randomized controlled trial. *J Compr Pediatr*. 2015;6(2)e26119.

S121. Baştürk A, Artan R, Atalay A, Yılmaz A. Investigation of the efficacy of synbiotics in the treatment of functional constipation in children: a randomized double-blind placebo-controlled study. *Turk J Gastroenterol*. 2017;28(5):388-393.

S122. Torabi Z, Amiraslani S, Diaz DN, Ahmadiafshar A, Eftekhari K. Comparison of paraffin versus polyethylene glycol (PEG) in children with chronic functional constipation. *Int J Pediatr*. 2017;5(10):5843-5850.

S123. Cao Y, Liu SM. Lactulose for the treatment of Chinese children with chronic constipation: A randomized controlled trial. *Medicine (Baltimore)*. 2018;97(52):e13794.

S124. Jose S, Ismael M. Effect of probiotics on constipation in children. *Int J Contemp Pediatr*. 2018;5(1):46-49.

S125. Cassettari VMG, Machado NC, Lourenção PLTdA, Carvalho MA, Ortolan EVP. Combinations of laxatives and green banana biomass on the treatment of functional constipation in children and adolescents: a randomized study. *J Pediatr (Rio J)*. 2019;95(1):27-33.

S126. Tjokronegoro SDP, Advani N, Firmansyah A. Effectiveness of probiotics in the management of functional constipation in children: A randomized, double-blind, placebo-controlled trial. *Int J Probiotics Prebiotics*. 2020;15:1-6.

S127. Dheivamani N, Thomas W, Bannerjii R, Mukherjee M, Mitra M. Efficacy of polyethylene glycol 3350 as compared to lactulose in treatment of ROME IV criteria-defined pediatric functional constipation: A randomized controlled trial. *Indian J Gastroenterol*. 2021;40(2):227-233.

S128. Worona-Dibner L, Vázquez-Frias R, Valdez-Chávez L, Verdiguel-Oyola M. Efficacy, safety, and acceptability of polyethylene glycol 3350 without electrolytes vs magnesium hydroxide in functional constipation in children from six months to eighteen years of age: A controlled clinical trial. *Rev Gastroenterol Mex (Engl Ed)*. 2023;88(2):107-117.

S129. Foroughi M, Ardakani AT, Taghizadeh M, Sharif MR. Comparing the effect of polyethylene glycol, psyllium seed husk powder, and probiotics on constipation in children. *Iran J Pediatrics*. 2022;32(5):e126565.

S130. Mansour HA, Ibrahim A, Mohamed A. Effectiveness of Polyethylene Glycol 3350 versus Lactulose in Management of Functional Constipation in Children. *Int J Pediatr Res*. 2022;8(1):089.
